# Halogenated *N*‑Benzylbenzisoselenazolones Efficiently Inhibit *Helicobacter pylori* Ureolysis *In Vitro*

**DOI:** 10.1021/acsmedchemlett.5c00057

**Published:** 2025-03-29

**Authors:** Marta Grabarek, Wojciech Tabor, Paweł Krzyżek, Agnieszka Grabowiecka, Łukasz Berlicki, Artur Mucha

**Affiliations:** † Department of Bioorganic Chemistry, Faculty of Chemistry, 49567 Wrocław University of Science and Technology, Wybrzeże Wyspiańskiego 27, 50-370 Wrocław, Poland; ‡ Department of Microbiology, Faculty of Medicine, Wrocław Medical University, Chałubińskiego 4, 50-368 Wrocław, Poland

**Keywords:** Organoselenium compounds, Bacterial ureases, Enzyme inhibitors, Covalent binding, Antimicrobial activity

## Abstract

Inspired by the recognized activity of Ebselen against urease, we optimized the structure of 1,2-benzisoselenazol-3­(2*H*)-one to provide potent inhibitors of ureolysis in *Helicobacter pylori* cells. To achieve this goal, we combined the elongation of the N-substituent of Ebselen from phenyl to benzyl with halogenation of the aromatic fragment. The modifications implemented provided compounds with activities that were several times better compared to that of the lead compound. In particular, 3-fluoro-4-trifluoromethyl and 2-chloro-5-fluoro derivatives of *N*-benzyl-1,2-benzisoselenazol-3­(2*H*)-one achieved a remarkable antiureolytic effect in live *H. pylori* cells (IC_50_ < 100 nM) that outperformed the data reported so far. This activity was reflected in the antiurease potential measured for the *Sporosarcina pasteurii* model enzyme, with the highest affinity observed for 2-chloro-5-fluoro and 2,4-dichloro derivatives (*K*_i_ < 0.6 nM). The best inhibitor demonstrated considerable antibacterial properties on a multidrug-resistant clinical *H. pylori* isolate in additive combination with clarithromycin (MIC = 0.073 μg/mL).

## Graphical abstract

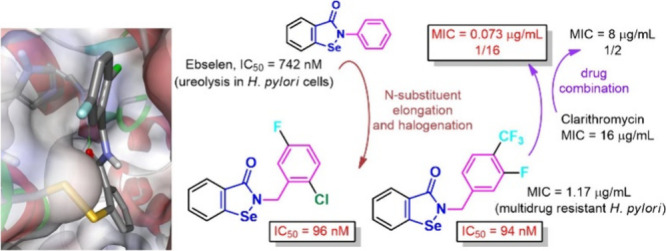

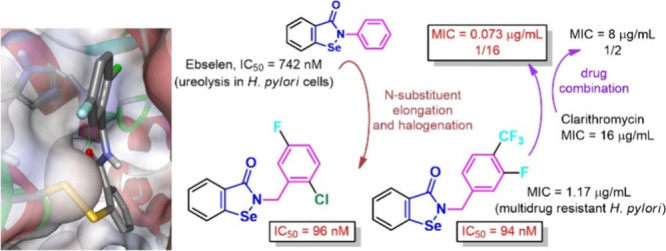

Approximately 43% of the world’s population appears to be affected by *Helicobacter pylori*,^1^ a Gram-negative helical-shaped bacterium discovered by Warren and Marshall more than 40 years ago.^2^ Although infection is often asymptomatic, *H. pylori* is considered an endemic human pathogen. Persistent stomach colonization causes gastric pathologies, such as chronic gastritis, followed by peptic and duodenal ulcers, with the risk of gastric cancers and gastric mucosa-associated lymphoid tissue lymphoma.^3^ Recent epidemiological studies have also correlated *H. pylori* infection with various pathologies that are not associated with the gastrointestinal tract, such as neurological, cardiovascular, dermatological, hematologic, allergic, and other diseases.^4,5^ As the progression of mucosal damage depends on the virulence of the bacterium, which is difficult to predict, and the infection is transmissible, the eradication of the pathogen is medically recommended. Common choices for first-line antibiotic treatment include standard bismuth quadruple therapy or triple therapy based on the proton pump inhibitor and clarithromycin.^6^ Generally, these established therapies do not target urease,^7,8^ cytoplasmic nickel-dependent hydrolase, which is the most abundant protein produced by *H. pylori*. Urease is considered the main virulence factor that allows toleration of the acidic environment of the gastroenteric tract,^9^ and serves as a biomarker for *H. pylori* infection.^10^ The enzyme processes urea that enters the cytoplasmic space through gene-regulated cell wall permeability under acidic conditions and is hydrolyzed to ammonia and carbon dioxide.^11,12^ The ammonia produced diffuses into the periplasm and low pH bacterial surroundings and buffers the environment around the cell surface.^13,14^ Since the mutant strain of *H. pylori* lacking urease is unable to colonize the host’s stomach,^15^ enzyme inhibition-based antivirulence approaches, including combinations with known antimicrobials, are considered promising alternatives to treat persistent gastric infections mediated by *H. pylori*.^16–18^

A countless number of low molecular weight chemical compounds have been evaluated for their ability to inhibit the activity of various urease enzymes, ureolytic extracts, and the growth of ureolytic bacterial strains, including *H. pylori*.^18–23^ Most of these inhibitors are noncovalent ligands that employ steric and electrostatic complementarity to the enzyme binding site, frequently assisted by nickel ion chelation. Covalent inhibitors, typically reactive with thiolate of the cysteine residue that regulates conformational changes critical to substrate accommodation during hydrolysis (Cys322 in *S. pasteurii* urease),^24^ are much less represented. Following the last-mentioned inhibition mechanism, we recently discovered various structures of high affinity as urease binders, namely organoselenium heterocycles,^25^ Michael acceptors^26^ and catechol-based phosphonates.^27^ In particular, Ebselen (**1**, *N*-phenyl-1,2-benzisoselenazol-3­(2*H*)-one, Scheme 1), recognized for its antioxidant, anti-inflammatory, and cytoprotective properties, and reactive with enzyme thiols,^28–30^ was characterized as a potent competitive and covalent inhibitor of *S. pasteurii* urease (*K*_i_ = 2.11 ± 0.18 nM).^25^ Following that original work, we verified several derivatives modified in the *N*-phenyl ring, including dihalogenated compounds, such as *N*-(4-chloro-2-fluorophenyl)-1,2-benzisoselenazol-3­(2*H*)-one (**2**, Scheme 1), which brought a striking level of inactivation of the model enzyme (*K*_i_ = 5.26 ± 0.41 pM).^31^ Three orders of magnitude improvement in inhibitory activity between **1** and **2** did not reflect a distinguishable change in control of ureolysis performed in *Proteus mirabilis* pathogenic cells; in both cases, this achieved excellent nanomolar parameters (IC_50_ = 29.2 ± 3.1 nM and IC_50_ = 24.6 ± 2.8 nM, respectively).^31^

**Scheme 1:**
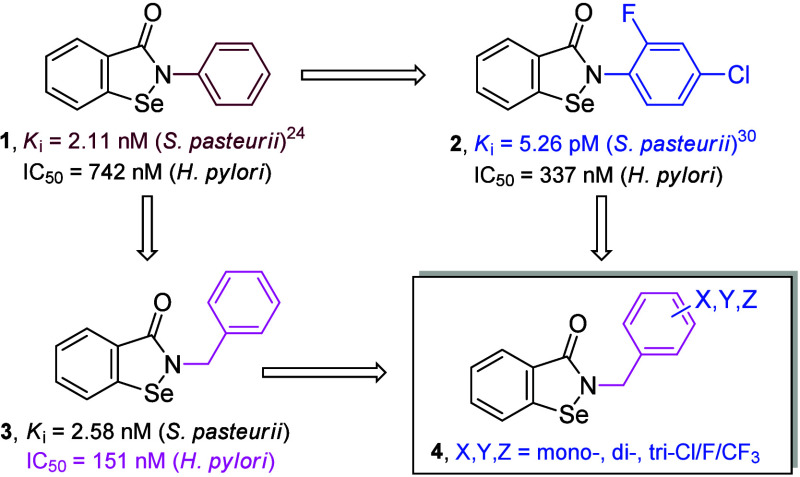
General Strategy of Structural Optimization of the N-Substituent of 1,2-Benzisoselenazol-3­(2*H*)-ones Starting from Ebselen (**1**), via a Dihalogenated (**2**) and N-Benzyl Derivative (**3**), to Compounds Containing Both Modifications (**4**), to Achieve an Improved Inhibition of *H. pylori* Ureolysis

In this work, we started with the evaluation of the potential of the two compounds mentioned (**1** and **2**) as inhibitors of ureolysis in live cells of *H. pylori* Tx30a (ATCC 51932). This specific strain was chosen for its lower sensitivity to environmental stress and limited transition to a coccoid form.^32^ The compounds showed a significant effect *in vitro*, with dihalogenated derivative **2** at a concentration twice lower than that of Ebselen (IC_50_ = 337 nM ± 24, **2** versus IC_50_ = 742 ± 41 nM, **1**, Scheme 1), but both were much less potent than they were against urea decomposition in *P. mirabilis*.

However, working in parallel with 1,2-benzisoselenazol-3­(2*H*)-ones other than N-aryl substituted, we recognized the N-benzyl derivative (**3**, Scheme 1) as a more perspective lead to target *H. pylori* ureolysis. Compound **3** inhibited the model urease to the same extent as Ebselen (*K*_i_ = 2.58 ± 0.18 nM), but it altered the ureolytic activity of pathogen cells at a concentration 5-fold lower than that of lead **1** (IC_50_ = 151 ± 5.7 nM, Scheme 1 and Table 1). To further explore the potential of this structural modification, we envisaged the synthesis of a series of 18 new differently halogenated *N*-benzyl-1,2-benzisoselenazol-3­(2*H*)-ones (**4a**–**r**, Schemes 1 and 2) and tested their activities against native *S. pasteurii* and ureolysis in *H. pylori* cells (Table 1).

**Scheme 2:**
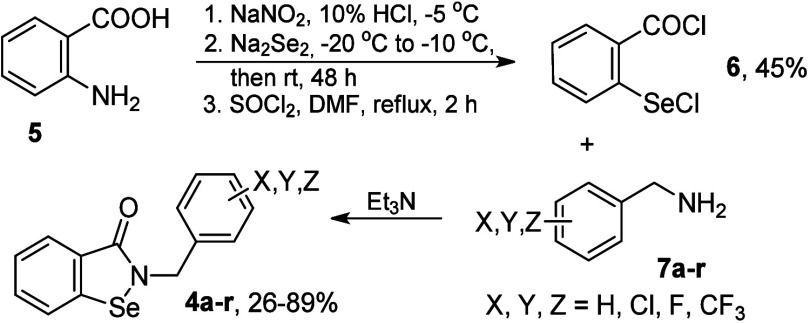
Synthesis of *N*-Benzyl-1,2-benzisoselenazol-3­(2*H*)-ones^a^

a See Table 1 for the pattern of substitution.

**Table 1:** Inhibitory Activity of Synthesized Compounds against the Urease of *S. pasteurii* and the Ureolysis of Live Cells of *H. pylori* (the Most Significant Data Indicated)

	Structure				
Entry	X	Y	Z	*K*_i_ [nM], *S. pasteurii* urease	IC_50_ [nM], *H. pylori* whole-cell ureolysis
**3**	H	H	H	2.58 ± 0.18	151 ± 5.7
**4a**	2-F	H	H	3.61 ± 0.28	171 ± 5.4
**4b**	3-F	H	H	12.2 ± 0.40	154 ± 8.4
**4c**	3-CF_3_	H	H	2.50 ± 0.24	112 ± 2.4
**4d**	4-F	H	H	25.4 ± 2.8	193 ± 8.1
**4e**	4-CF_3_	H	H	2.10 ± 0.19	170 ± 1.6
**4f**	2-Cl	4-Cl	H	**0.594 ± 0.032**	345 ± 16
**4g**	2-F	4-F	H	2.52 ± 0.19	287 ± 18
**4h**	2-Cl	5-Cl	H	9.04 ± 0.22	341 ± 23
**4i**	2-Cl	5-F	H	**0.543 ± 0.015**	**96.4 ± 1.7**
**4j**	2-F	6-F	H	1.16 ± 0.14	218 ± 14
**4k**	3-Cl	4-Cl	H	15.9 ± 0.65	315 ± 4.7
**4l**	3-F	4-F	H	3.35 ± 0.65	110 ± 4.5
**4m**	3-F	4-CF_3_	H	15.90 ± 1.9	**93.5 ± 5.3**
**4n**	3-CF_3_	4-Cl	H	18.6 ± 0.37	153 ± 5.4
**4o**	3-CF_3_	4-F	H	2.99 ± 0.088	107 ± 1.9
**4p**	3-F	5-F	H	1.30 ± 0.027	122 ± 4.1
**4q**	3-CF_3_	5-CF_3_	H	3.32 ± 0.29	208 ± 4.5
**4r**	2-F	3-F	4-F	2.94 ± 0.26	179 ± 3.3

The synthesis of the target compounds involved a well-established procedure of aminolysis of 2-(chloroseleno)­benzoyl chloride (**6**, Scheme 2).^33,34^ This reactive selenium compound was obtained by diazotization of anthranilic acid (**5**), which was followed by a reaction with disodium diselenide and chlorination of intermediate bis­(carboxyphenyl)­diselenide with thionyl chloride. Variously halogenated benzylamines, mono-, di-, or trisubstituted with fluorine, chlorine, or trifluoromethyl group (**7a**–**r**), were used as nucleophiles to form the selenazolone ring. Principally, the yields of aminolysis varied from good to excellent, while only the presence of the electron-withdrawing trifluoromethyl group, in particular, multiply substituted, significantly reduced the benzylamine nucleophilicity and reaction efficacy (see the Supporting Information for details). The products easily crystallized; thus, the nonproblematic workup involved acid and base extraction, drying, and evaporation of the organic phase, and then filtration of the crystallized pure product.

The native urease, purified from *S. pasteurii* CCM 2056, was consequently used as the model bacterial enzyme to measure the potency of *N*-benzylbenzisoselenazolones **4a**–**r**. This enzyme is the most frequently studied for the evaluation of new inhibitors, as its preparation, kinetics, and structure to discuss the relationship with activity in a broad context are well recognized.^8,23,35^ The synthesized compounds acted as potent slow-binding inhibitors, typical for this type of scaffold. Close structural similarities cause the affinity of *N*-benzylbenzisoselenazolones against the *S. pasteurii* urease expressed as *K*_i_ to not vary in a broad range. The difference between the most (**4f** and **4i**, *K*_i_ = 0.5–0.6 nM) and the least potent derivative (**4d**, **4m**, and **4n**, *K*_i_ = 15–25 nM) exceeded an order of magnitude (Table 1). Mono and difluorinated/chlorinated compounds were typically found to be more active than their trifluoromethylated counterparts. The majority of representatives could be classified as excellent inhibitors, with inhibition constants with a low nanomolar value (*K*_i_ < 10 nM). Certain compounds, such as **4f** (*K*_i_ = 0.594 ± 0.032 nM), **4i** (*K*_i_ = 0.543 ± 0.015), **4j** (*K*_i_ = 1.16 ± 0.14 nM), and **4p** (*K*_i_ = 1.30 ± 0.027 nM), outscored in affinity the lead compounds, Ebselen **1** (*K*_i_ = 2.11 nM ± 0.18) and nonhalogenated *N*-benzylbenzisoselenazolone **3** (*K*_i_ = 2.58 ± 0.18 nM). However, even the best inhibitors did not achieve the striking potency of previously reported dihalogenated Ebselens (exemplified by compound **2**, *K*_i_ = 5.26 ± 0.41 pM).

The ability of inhibitors **4a**–**r** to control urea decomposition in live cells of *H. pylori* Tx30a varied even in a narrower range and reached an impressive level of the IC_50_ value at a submicromolar concentration (IC_50_ = 93.5 – 345 nM, Table 1). This concentration was significantly lower than that of Ebselen **1** (IC_50_ = 742 ± 41 nM), and for the dominant majority of compounds than its dihalogenated derivative **2** (IC_50_ = 337 nM), which was so potent with the native enzyme of *S. pasteurii.* Six compounds showed better inhibitory strength than nonhalogenated *N*-benzylbenzisoselenazolone **3** (IC_50_ = 151 ± 5.7 nM). IC_50_ below 100 nM was measured for compound **4i** (IC_50_ = 96.4 ± 1.7 nM) and **4m** (IC_50_ = 93.5 ± 5.3 nM), making them the most potent found in the study and, to the best of our knowledge, antiureolytic agents active on *H. pylori* cells.^18^ No significant dependence of the antiureolytic activity on the type or pattern of substitution with halogen(s) was observed. The structure of **4i** was found to be optimal: the compound with a predominant affinity for the pure enzyme appeared to be one of the best individuals in the control of ureolysis in cells.

The molecular mechanism of inhibition of urease with benzisoselenazolones involves the opening of the five-membered heterocyclic ring and the formation of the S–Se covalent adduct. This alters the access of the substrate to catalytic nickel ions and the function of the mobile flap to adopt the closed conformation indispensable for catalysis. As illustrated by molecular modeling, the binding of **4i** provides a favorable complementarity to the active site of both enzymes (Figure 1). Microbial ureases may differ in supramolecular subunit organization but share a high similarity in catalytic site architecture and mechanism.^24,36^ Accordingly, the individual conformations of the hydrophobic aromatic fragments of inhibitor **4i** slightly varied, but the general mode of interaction remained well conserved (see the Supporting Information for another example, **4f**, Figures S1 and S2). Specifically, in the case of *S. pasteurii* urease, the halogenated ring of the flexible benzyl residue of **4i** primarily faced the positively charged guanidino group of Arg339 to form a cation-π interaction, while for the *H. pylori* enzyme, its twist made possible the corresponding contact with the imidazolium ion of His322 neighboring the reactive cysteine. In both adducts, a hydrogen bond was clearly visible between the oxygen atom of the C=O group of the ligand and NH of the guanidino group of Arg339/Arg338. On the contrary, no apparent halogen bonds were evidenced.

**Figure 1:**
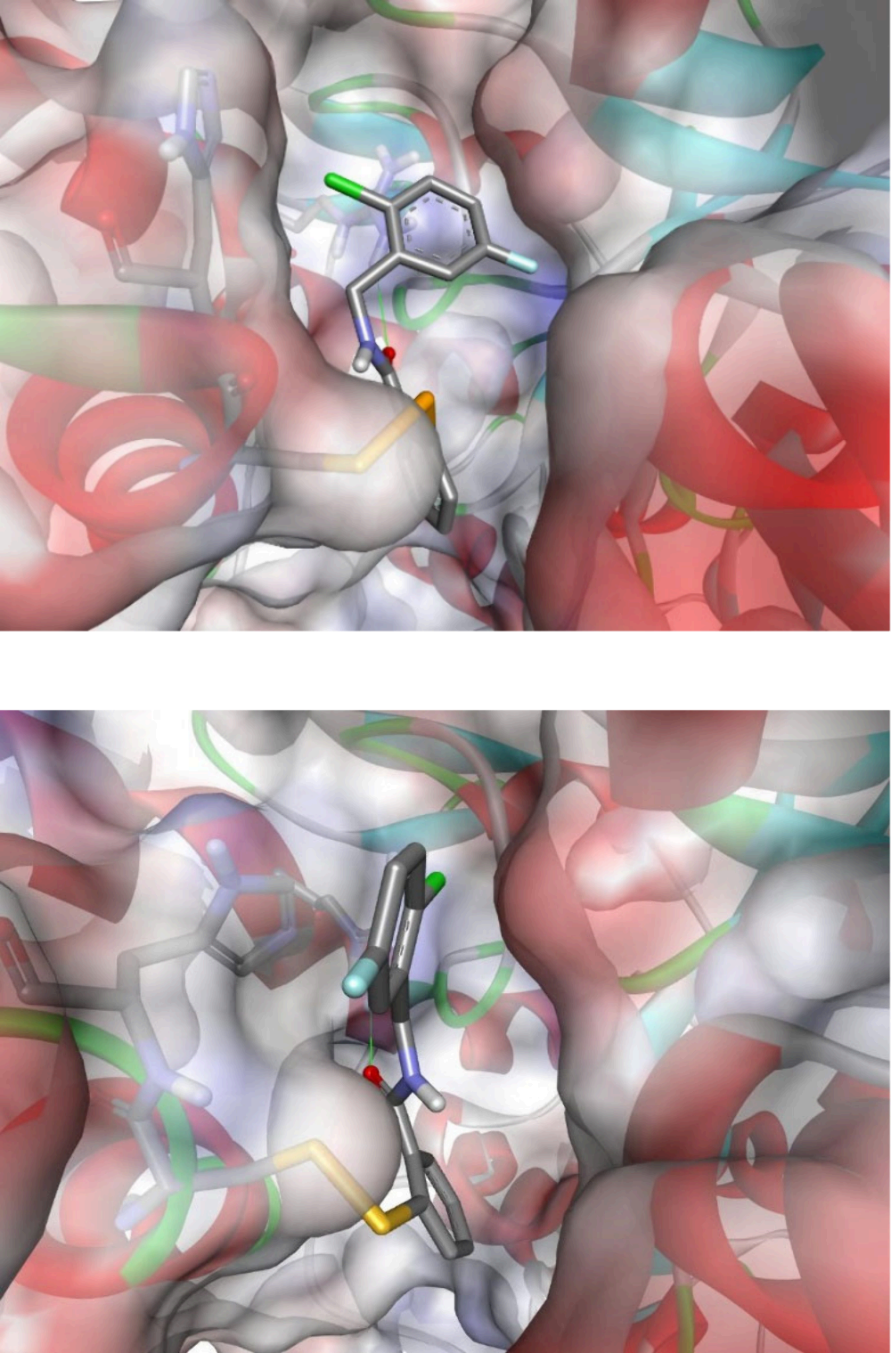
Modeled structures of ureases from *S. pasteurii* (top panel, PDB id 5G4H)^37^ and *H. pylori* (bottom panel, PDB id 1E9Y)^38^ with inhibitor **4i** bound to the Cys322/Cys321 residues of enzymes (numbering of the α subunit of (αβγ)_3_ or ((αβ)_3_)_4_ quaternary structures, respectively). The inhibitor is shown as sticks colored according to the atom type (gray, carbon; blue, nitrogen; white, hydrogen; red, oxygen; yellow, sulfur; orange, selenium; green, chlorine; light blue, fluorine). The proteins are shown as ribbons with the solvent-accessible surface colored according to the interpolated charge. The hydrogen bond is shown as a thin, solid green line.

The antimicrobial activity of the most potent inhibitor of *H. pylori* Tx30a whole cell ureolysis (**4m**) was further characterized on a clinical isolate *H. pylori* 2CML, and compared to Ebselen, **1**. The *H. pylori* 2CML strain was originally isolated from a primary infected (noneradicated) patient with gastritis and presents multidrug resistance (clarithromycin, metronidazole, and levofloxacin) and the ability to quickly adapt to unfavorable environmental conditions by forming antibiotic-tolerant coccoid forms and biofilm.^39^ The MIC value for both compounds, **1** and **4m**, measured alone, was 3.125 μM (0.857 and 1.17 μg/mL, respectively, Table 2). The combination of Ebselen with the three commercial antibiotics was additive and produced a 2-fold reduction in MIC values for both components (to provide FICI = 1), the same as for benzisoselenazolone **4m** and levofloxacin. The FIC index decreased substantially for the interaction of compound **4m** with clarithromycin and metronidazole. Simultaneous addition reduced the MIC value of both antibiotics twice, but in the case of the selenium inhibitor, it was 16 and eight times, respectively. Therefore, a very promising MIC = 0.0731 μg/mL (0.2 μM) was achieved for **4m** in the presence of clarithromycin, maintained at a high concentration.

**Table 2:** Antimicrobial Effects of Organoselenium Compound **4m** Compared to Ebselen, Evaluated Alone and in Combination with Antibiotics Clarithromycin, Metronidazole, and Levofloxacin, against Multidrug-Resistant Clinical Isolate *H. pylori* 2CML

	MIC [μg/mL]					
Antimicrobials	Alone	Combination	Fold change	FIC	FICI	Interpretation
Ebselen	0.857	0.429	×2↓	0.5	1	Additive
Clarithromycin	16	8	×2↓	0.5		
Ebselen	0.857	0.429	×2↓	0.5	1	Additive
Metronidazole	64	32	×2↓	0.5		
Ebselen	0.857	0.429	×2↓	0.5	1	Additive
Levofloxacin	16	8	×2↓	0.5		
**4m**	1.17	**0.0731**	**×16↓**	0.0625	0.562	Additive
Clarithromycin	16	8	×2↓	0.5		
**4m**	1.17	0.146	×8↓	0.125	0.625	Additive
Metronidazole	64	32	×2↓	0.5		
**4m**	1.17	0.585	×2↓	0.5	1	Additive
Levofloxacin	16	8	×2↓	0.5		

In summary, urease inhibitors have recently emerged as promising drug candidates for the treatment of *H. pylori* infections.^18,20^ Here, this assumption was supported by the data for halogenated N-benzylbenzisoselenozolones, which demonstrated unprecedented antiureolytic activity with live pathogen cells. Submicromolar potency with intact *H. pylori* cells has been uniquely reported, as for noncovalent hydroxamate-based inhibitors.^40^ Preliminary studies with a drug-resistant isolate also confirmed the high antibacterial potential of developed compounds, particularly in combination with clinically used antibiotics, which might reflect beneficial effects on their toxicity profile and limit side effects in patients. These characteristics associated with known advantages of Ebselen and its analogs, such as versatile synthesis, recognized multitarget and antimicrobial-specific mechanisms of action (e.g., inhibition of bacterial thioredoxin reductase, thus blocking the disulfide reduction system in multiple substrates that alters the biosynthesis of DNA and essential cellular proteins, influences cell redox status, and induces oxidative stress),^30,41^ low drug resistance appearance, and reasonable toxicity, predestine them for further investigations targeting persistent human infections.

## Supplementary Material



## Notes

Experimental methods for the synthesis of compounds with detailed characterization, methods for measurement of ureolysis inhibition and antimicrobial activity, and the molecular modeling protocol, with interaction mode of compound **4f** with ureases; ^1^H, ^13^C, ^19^F, and ^77^Se NMR spectra and analytical HPLC traces of the final compounds **3** and **4a**–**r** (PDF)

## Abbreviations

MIC: minimum inhibitory concentration
FICI: fractional inhibitory concentration (FIC) index
