# Supplementary material for: Halogenated N‑Benzylbenzisoselenazolones Efficiently Inhibit Helicobacter pylori Ureolysis In Vitro
Source: ACS Med Chem Lett. 2025 Mar 29;16(4):675–80. doi: 10.1021/acsmedchemlett.5c00057 (PMC11995230; doi:10.1021/acsmedchemlett.5c00057)
Supplement: Supplementary file 1 [file ml5c00057_si_001.pdf]

Supporting Information

Halogenated *N*-benzylbenzisoselenazolones efficiently inhibit *Helicobacter pylori* ureolysis *in vitro*

Marta Grabarek,[1] Wojciech Tabor,[1] Paweł Krzyżek,[2] Agnieszka Grabowiecka,[1] Łukasz Berlicki[1] and Artur Mucha[1]*

[1] Department of Bioorganic Chemistry, Faculty of Chemistry, Wrocław University of Science and Technology, Wybrzeże Wyspiańskiego 27, 50-370 Wrocław, Poland
[2] Department of Microbiology, Faculty of Medicine, Wrocław Medical University, Chałubińskiego 4, 50-368 Wrocław, Poland

Contents

1. Experimental. Chemistry. General methods S2

2. *N*-Benzyl-1,2-benzisoselenazol-3(2*H*)-ones S2

3. Biological studies S7

4. Molecular modeling S9

5. References S11

6. NMR spectra and HPLC analyses S12

1. Experimental. Chemistry. General methods

All reagents used were purchased from the following commercial suppliers: Merck Poland – Sigma–Aldrich, Avantor Performance Materials Poland, Chemland Poland, and Stanlab Poland; they were of analytical grade and were used without further purification. The melting points were determined on an Electrothermal IA 91100 digital melting point apparatus using the standard open capillary method. The ^{1}H, ^{13}C, ^{19}F and ^{77}Se NMR spectra were recorded in acetone-d_6 or DMSO-d_6 on a Bruker Avance II 600 or Jeol ECZ 400S spectrometer at frequencies 600.6 or 399.8 MHz (^{1}H), 151.0 or 100.5 MHz (^{13}C), 376.2 MHz (^{19}F), 76.2 MHz (^{77}Se), respectively, at 295 K. Chemical shifts were reported in parts per million (ppm, δ) downfield from tetramethylsilane. Residual solvent central signals were recorded as follows: acetone-d_6, δ_H = 2.05 ppm, δ_C = 206.13 and 29.84 ppm, DMSO-d_6, δ_H = 2.50 ppm, δ_C = 39.52 ppm. Coupling patterns were described as singlet (*s*), doublet (*d*), triplet (*t*), quartet (*q*), and multiplet (*m*). High-resolution mass spectra (HRMS) were recorded using an electron spray ionization (ESI) technique on a Waters LCT Premier XE spectrometer. Analytical reversed-phase high-performance liquid chromatography was performed using the UFLC Shimadzu system and Reprosil Saphir 100C18 column, 4.6 × 150 mm (0 min, 2% B → 2 min, 2% B → 20 min, 90% B → 25 min, 90% B → 30 min, 2% B), flow 0.9 mL/min. Solvent A: 0.1% TFA in water, solvent B: 0.1% TFA in acetonitrile. Chromatograms were recorded at wavelengths of 222 and 254 nm using background compensation. The final *N*-benzyl-1,2-benzisoselenazol-3(*2H*)-ones **3** and **4a-r** gave satisfactory NMR and HRMS spectra and were >95% pure.

2. *N*-Benzyl-1,2-benzisoselenazol-3(*2H*)-ones

The synthesis of the target compounds was performed according to the procedure described for aminolysis of 2-(chloroseleno)benzoyl chloride with minor modifications.[S1,S2]

Disodium diselenide. In a round-bottom three-neck flask equipped with an oil cap and a thermometer, sodium hydroxide (0.75 mol, 30.0 g) dissolved in methanol (300 ml), powdered selenium (0.25 mol, 19.7 g) and 100% hydrazine hydrate (64 mmol, 3.2 g) were mixed for 48 h at room temperature. **Diazonium salt of anthranilic acid.** In a beaker, anthranilic acid (0.25 mol, 34.3 g) was dissolved in a hot 10% aqueous hydrochloric acid solution (200 ml). The solution was then cooled in an ice bath and a cold sodium nitrate solution (0.28 mol, 18.6 g in 100 ml of water) was added in portions. **2,2'-Diselenobis(benzoic acid).** The cold diazonium salt solution was added dropped to the disodium diselenide solution cooled to -20°C, so that the temperature of the reaction mixture did not exceed -10°C. The reaction was continued at room temperature for 48 h. Then, the unreacted selenium was filtered off and the filtrate was heated to boiling with activated carbon. After filtration, the solution was acidified with concentrated hydrochloric acid. The precipitate was filtered under reduced pressure, washed with hot water and recrystallized from 1,4-dioxane to yield 23.0 g (46%) of a solid of cream color. ^{1}H NMR (400 MHz, DMSO-d_6) δ 7.99 (dd, *J* = 7.7, 1.6 Hz, 1H), 7.63 (dd, *J* = 8.0, 0.6 Hz, 1H), 7.45 (ddd, *J* = 8.0, 7.7, 1.6 Hz, 1H), 7.32 (m, 1H). ^{13}C NMR (100 MHz, DMSO-d_6) δ 169.10, 134.16, 133.99, 132.12, 130.02, 129.27, 127.09. HRMS (ESI) *m/z* calculated for $C_{14}H_{10}O_4Se_2$+Na^+ 424.8807, found 424.8795. **2-(Chloroseleno)benzoyl chloride.** 2,2'-Diselenobis(benzoic acid) (49 mmol, 19.5 g), thionyl chloride (0.49 mol, 58.0 g) and DMF (1 mL) were refluxed for 2 h. After being cooled to room temperature, the volatiles were evaporated *in vacuo*. The oil was dissolved in toluene and evaporation was repeated under reduced pressure. The crude product was recrystallized from hexane to yield 24.2 g (98%) of orange-yellow crystals.

1,2-Benzisoselenazol-3(*2H*)-ones. General procedure. 2-(Chloroseleno)benzoyl chloride (1 eq.) dissolved in anhydrous dichloromethane (1 ml for 1 mmol) was added to a solution of a benzylamine (1.0 eq.) and anhydrous triethylamine (3 eq.) in anhydrous dichloromethane (5 mL).

After stirring at room temperature for 48 h, a 5% aqueous sodium bicarbonate solution (20 ml) was added to the mixture and the product was extracted with methylene chloride (50 ml). The organic phase was then washed with a 5% aqueous sodium bisulfate solution (20 ml) and brine (20 ml). The solution was dried over anhydrous Na_2SO_4 and concentrated *in vacuo*. The crystalline residue was worked up with diethyl ether, filtered, and washed with ether.

***N*-Benzyl-1,2-benzisoselenazol-3(*2H*)-one (3).**[S3,S4] Pale yellow solid, yield 63%, mp 137-139°C. ^{1}H NMR (400 MHz, DMSO-*d*$_6$) δ 8.00 (d, *J* = 8.1 Hz, 1H), 7.85 (dd, *J* = 7.8, 1.5 Hz, 1H), 7.61 (ddd, *J* = 8.4, 7.2, 1.5 Hz, 1H), 7.43 (ddd, *J* = 8.0, 7.2, 1.0 Hz, 1H), 7.38 – 7.25 (m, 5H), 4.91 (s, 2H). ^{13}C NMR (101 MHz, DMSO-*d*$_6$) δ 166.45, 139.37, 138.38, 131.70, 128.64, 128.01, 127.87, 127.60, 127.57, 125.97, 125.94, 46.78. ^{77}Se NMR (76 MHz, DMSO-*d*$_6$) δ 858.78. HRMS (ESI) *m/z* calculated for $C_{14}H_{11}NOSe+H^+$ 290.0084, found 290.0089.

***N*-(2-Fluorobenzyl)-1,2-benzisoselenazol-3(*2H*)-one (4a).** White solid, yield 50%, mp 153-154°C. ^{1}H NMR (600 MHz, acetone-*d*$_6$) δ 7.98 (d, *J* = 8.0 Hz, 1H), 7.95 (dd, *J* = 7.6, 1.8 Hz, 1H), 7.64 (ddd, *J* = 8.3, 7.2, 1.5 Hz, 1H), 7.47 (ddd, *J* = 7.7, 7.7, 1.1 Hz, 1H), 7.42 (ddd, *J* = 7.6, 7.6, 2.0 Hz, 1H), 7.38 (dddd, *J* = 7.4, 7.4, 5.4, 1.8 Hz, 1H), 7.18 (m, 2H), 5.05 (s, 1H). ^{13}C NMR (151 MHz, acetone-*d*$_6$) δ 167.53, 161.67 (d, *J* = 245.8 Hz), 139.70, 132.79, 131.53 (d, *J* = 3.8 Hz), 130.88 (d, *J* = 8.2 Hz), 128.93, 128.43, 126.92, 126.09, 125.97, 125.47 (d, *J* = 3.8 Hz), 116.16 (d, *J* = 21.3 Hz), 41.91 (d, *J* = 4.9 Hz). ^{19}F NMR (376 MHz, acetone-*d*$_6$) δ -119.46 (m). ^{77}Se NMR (76 MHz, acetone-*d*$_6$) δ 879.51 (d, *J* = 17.2 Hz). HRMS (ESI) *m/z* calculated for $C_{14}H_{10}FNOSe+H^+$ 307.9990, found 307.9996.

***N*-(3-Fluorobenzyl)-1,2-benzisoselenazol-3(2H)-one (4b).** Pale yellow solid, yield 41%, mp 138-140°C. ^{1}H NMR (600 MHz, DMSO-*d*$_6$) δ 8.09 (d, *J* = 8.0 Hz, 1H), 7.85 (dd, *J* = 7.7, 1.2 Hz, 1H), 7.61 (ddd, *J* = 8.0, 8.0, 1.7 Hz, 1H), 7.43 (ddd, *J* = 7.4, 7.4, 1.0 Hz, 1H), 7.39 (m, 1H), 7.17 – 7.08 (m, 3H), 4.92 (s, 2H). ^{13}C NMR (101 MHz, DMSO-*d*$_6$) δ 166.61, 162.28 (d, *J* = 243.9 Hz), 141.48 (d, *J* = 7.2 Hz), 139.63, 131.70, 130.65 (d, *J* = 8.3 Hz), 127.83, 127.56, 126.21, 125.96, 123.94 (d, *J* = 2.9 Hz), 114.60 (d, *J* = 21.5 Hz), 114.29 (d, *J* = 20.7 Hz), 46.10. ^{19}F NMR (376 MHz, DMSO-*d*$_6$) δ -113.04 (m). ^{77}Se NMR (76 MHz, DMSO-*d*$_6$) δ 857.44. HRMS (ESI) *m/z* calculated for $C_{14}H_{10}FNOSe+H^+$ 307.9990, found 307.9996.

***N*-(3-(Trifluoromethyl)benzyl)-1,2-benzisoselenazol-3(*2H*)-one (4c).** White solid, yield 53%, mp 134-135°C. ^{1}H NMR (600 MHz, acetone-*d*$_6$) δ 7.99 (d, *J* = 8.0 Hz, 1H), 7.97 (dd, *J* = 7.8, 1.7 Hz, 1H), 7.76 (s, 1H), 7.71 – 7.58 (m, 4H), 7.49 (ddd, *J* = 7.8, 7.5, 1.0 Hz, 1H), 5.12 (s, 2H). ^{13}C NMR (101 MHz, acetone-*d*$_6$) δ 167.53, 140.62, 139.57, 132.72, 132.69, 130.93 (q, *J* = 32.0 Hz), 130.27, 128.80, 128.12, 126.78, 125.98, 125.40 (q, *J* = 4.0 Hz), 125.05 (q, *J* = 3.9 Hz), 125.04 (d, *J* = 271.7 Hz), 47.41. ^{19}F NMR (376 MHz, acetone-*d*$_6$) δ -62.96. ^{77}Se NMR (76 MHz, acetone-*d*$_6$) δ 883.95. HRMS (ESI) *m/z* calculated for $C_{15}H_{10}F_3NOSe+H^+$ 357.9958, found 357.9954.

***N*-(4-Fluorobenzyl)-1,2-benzisoselenazol-3(*2H*)-one (4d).** White solid, yield 53%, mp 153-155°C. ^{1}H NMR (600 MHz, acetone-*d*$_6$) δ 7.96 (m, 2H), 7.63 (ddd, *J* = 8.2, 7.2, 1.5 Hz, 1H), 7.47 (ddd, *J* = 8.2, 7.2, 1.0 Hz, 1H), 7.46 – 7.43 (m, 2H), 7.12 (dd, *J* = 8.9, 8.9 Hz, 2H), 4.98 (s, 2H). ^{13}C NMR (151 MHz, acetone-*d*$_6$) δ 167.50, 163.23 (d, *J* = 244.1 Hz), 139.69, 135.47 (d, *J* = 3.3 Hz), 132.75, 131.20 (d, *J* = 8.7 Hz), 128.94, 128.61, 126.90, 126.08, 116.17 (d, *J* = 21.8 Hz), 47.51. ^{19}F NMR (376 MHz, acetone-*d*$_6$) δ -116.11 (m). ^{77}Se NMR (76 MHz, acetone-*d*$_6$) δ 876.98. HRMS (ESI) *m/z* calculated for $C_{14}H_{10}FNOSe+H^+$ 307.9990, found 307.9993.

***N*-(4-(Trifluoromethyl)benzyl)-1,2-benzisoselenazol-3(*2H*)-one (4e).** White solid, yield 38%, mp 156-157°C. ^{1}H NMR (600 MHz, acetone-d_6) δ 7.99 (d, *J* = 8.2 Hz, 1H), 7.97 (dd, *J* = 7.9, 2.1 Hz, 1H), 7.71 (d, *J* = 8.1 Hz, 2H), 7.65 (ddd, *J* = 8.2, 7.2, 1.5 Hz, 1H), 7.60 (d, *J* = 8.1 Hz, 2H), 7.49 (ddd, *J* = 7.9, 7.2, 1.0 Hz, 1H), 5.11 (s, 2H). ^{13}C NMR (151 MHz, acetone-d_6) δ 167.72, 143.98, 139.75, 132.91, 130.08 (q, *J* = 32.2 Hz), 129.53, 129.02, 128.29, 126.99, 126.36 (q, *J* = 4.1 Hz), 126.16, 125.34 (d, *J* = 271.3 Hz), 47.63. ^{19}F NMR (376 MHz, acetone-d_6) δ -62.86. ^{77}Se NMR (76 MHz, acetone-d_6) δ 885.50. HRMS (ESI) *m/z* calculated for $C_{15}H_{10}F_3NOSe+H^+$ 357.9958, found 357.9957.

***N*-(2,4-Dichlorobenzyl)-1,2-benzisoselenazol-3(*2H*)-one (4f).** White solid, yield 82%, mp 196-198°C. ^{1}H NMR (600 MHz, DMSO-d_6) δ 8.03 (d, *J* = 8.1 Hz, 1H), 7.85 (dd, *J* = 7.8, 1.6 Hz, 1H), 7.68 (d, *J* = 2.2 Hz, 1H), 7.63 (ddd, *J* = 8.4, 7.1, 1.6 Hz, 1H), 7.47 – 7.41 (m, 2H), 7.27 (d, *J* = 8.4 Hz, 1H), 4.96 (s, 2H). ^{13}C NMR (101 MHz, DMSO-d_6) δ 166.85, 139.69, 134.85, 133.72, 133.35, 132.09, 131.25, 129.13, 127.91, 127.78, 127.59, 126.26, 126.13, 44.36. ^{77}Se NMR (76 MHz, DMSO-d_6) δ 868.93. HRMS (ESI) *m/z* calculated for $C_{14}H_9Cl_2NOSe+H^+$ 357.9305, found 357.9310.

***N*-(2,4-Difluorobenzyl)-1,2-benzisoselenazol-3(*2H*)-one (4g).** White solid, yield 89%, mp 187-188°C. ^{1}H NMR (600 MHz, acetone-d_6) δ 7.98 (d, *J* = 8.0 Hz, 1H), 7.94 (dd, *J* = 7.9, 1.5 Hz, 1H), 7.64 (ddd, *J* = 8.3, 7.2, 1.5 Hz, 1H), 7.52 – 7.46 (m, 2H), 7.07 (ddd, *J* = 10.3, 9.1, 2.5 Hz, 1H), 7.02 (dddd, *J* = 8.5, 8.5, 2.5, 1.0 Hz, 1H), 5.03 (s, 2H). ^{13}C NMR (151 MHz, acetone-d_6) δ 167.54, 163.56 (dd, *J* = 247.6, 11.8 Hz), 161.75 (dd, *J* = 248.3, 12.5 Hz), 139.67, 132.85, 132.83 (dd, *J* = 9.2, 5.5 Hz), 128.93, 128.36, 126.96, 126.10, 122.42 (dd, *J* = 15.3, 3.5 Hz), 112.41 (dd, *J* = 21.2, 3.8 Hz), 104.54 (t, *J* = 26.0 Hz), 41.45 (d, *J* = 4.2 Hz). ^{19}F NMR (376 MHz, acetone-d_6) δ -111.78 (m), -114.82 (m). ^{77}Se NMR (76 MHz, acetone-d_6) δ 876.17 (d, *J* = 17.9 Hz). HRMS (ESI) *m/z* calculated for $C_{14}H_9F_2NOSe+H^+$ 325.9896, found 325.9905.

***N*-(2,5-Dichlorobenzyl)-1,2-benzisoselenazol-3(*2H*)-one (4h).** White solid, yield 62%, mp 205-207°C. ^{1}H NMR (600 MHz, DMSO-d_6) δ 8.04 (d, *J* = 8.0 Hz, 1H), 7.86 (dd, *J* = 7.8, 1.6 Hz, 1H), 7.64 (ddd, *J* = 8.3, 7.1, 1.6 Hz, 1H), 7.55 (d, *J* = 8.5 Hz, 1H), 7.48 – 7.41 (m, 2H), 7.28 (d, *J* = 2.6 Hz, 1H), 4.97 (s, 2H). ^{13}C NMR (101 MHz, DMSO-d_6) δ 166.66, 139.60, 137.84, 132.01, 131.91, 131.23 (2C), 129.26, 129.11, 127.59, 127.38, 126.05 (2C), 44.40. ^{77}Se NMR (76 MHz, DMSO-d_6) δ 868.95. HRMS (ESI) *m/z* calculated for $C_{14}H_9Cl_2NOSe+H^+$ 357.9305, found 357.9310.

***N*-(2-Chloro-5-fluorobenzyl)-1,2-benzisoselenazol-3(*2H*)-one (4i).** White solid, yield 59%, mp 183-184°C. ^{1}H NMR (600 MHz, acetone-d_6) δ 8.02 (d, *J* = 8.0 Hz, 1H), 7.97 (dd, *J* = 7.8, 1.7 Hz, 1H), 7.67 (ddd, *J* = 8.4, 7.2, 1.5 Hz, 1H), 7.54 – 7.47 (m, 2H), 7.19 – 7.12 (m, 2H), 5.08 (s, 2H). ^{13}C NMR (151 MHz, acetone-d_6) δ 167.80, 162.46 (d, *J* = 244.8 Hz), 139.92, 138.99, 132.98, 132.04 (d, *J* = 9.0 Hz), 129.00, 128.08, 127.02, 126.20, 117.47 (d, *J* = 23.6 Hz), 117.10 (d, *J* = 22.9 Hz), 45.77. ^{19}F NMR (376 MHz, acetone-d_6) δ -115.74 (m). ^{77}Se NMR (76 MHz, acetone-d_6) δ 880.78. HRMS (ESI) *m/z* calculated for $C_{14}H_9ClFNOSe+H^+$ 341.9600, found 341.9590.

***N*-(2,6-Difluorobenzyl)-1,2-benzisoselenazol-3(*2H*)-one (4j).** White solid, yield 74%, mp 192-193°C. ^{1}H NMR (600 MHz, acetone-d_6) δ 7.95 (d, *J* = 8.1 Hz, 1H), 7.93 (dd, *J* = 7.7, 1.5 Hz, 1H), 7.62 (ddd, *J* = 8.2, 7.1, 1.4 Hz, 1H), 7.53 – 7.43 (m, 2H), 7.09 (dd, *J* = 8.1, 8.1 Hz, 2H), 5.12 (s, 2H). ^{13}C NMR (151 MHz, acetone-d_6) δ 167.07, 162.49 (dd, *J* = 249.0, 7.6 Hz), 139.36, 132.78, 131.90 (t, *J* = 10.4 Hz), 128.93, 128.42, 126.95, 126.02, 114.59 (t, *J* = 19.4 Hz), 112.47 (dd, *J* = 21.2, 4.5 Hz), 35.86 (t, *J* = 3.8 Hz). ^{19}F NMR (376 MHz, acetone-d_6) δ -114.81 (m).

^{77}Se NMR (76 MHz, acetone-*d*$_6$) δ 872.54 (t, *J* = 21.9 Hz). HRMS (ESI) *m/z* calculated for $C_{14}H_9F_2NOSe+H^+$ 325.9896, found 325.9905.

***N*-(3,4-Dichlorobenzyl)-1,2-benzisoselenazol-3(*2H*)-one (4k).** Pale yellow solid, yield 74%, mp 154-156°C. ^{1}H NMR (600 MHz, DMSO-*d*$_6$) δ 8.04 (d, *J* = 7.9 Hz, 1H), 7.85 (dd, *J* = 7.8, 1.6 Hz, 1H), 7.62 (ddd, *J* = 8.4, 7.1, 1.6 Hz, 1H), 7.61 (d, *J* = 8.3 Hz, 1H), 7.59 (d, *J* = 2.1 Hz, 1H), 7.44 (ddd, *J* = 7.6, 7.1, 0.9 Hz, 1H), 7.29 (dd, *J* = 8.3, 2.1 Hz, 1H), 4.92 (s, 2H). ^{13}C NMR (101 MHz, DMSO-*d*$_6$) δ 166.65, 139.77, 139.56, 131.81, 131.07, 130.83, 130.03, 129.86, 128.26, 127.58 (2C), 126.08, 126.00, 45.43. ^{77}Se NMR (76 MHz, DMSO-*d*$_6$) δ 863.65. HRMS (ESI) *m/z* calculated for $C_{14}H_9Cl_2NOSe+H^+$ 357.9305, found 357.9310.

***N*-(3,4-Difluorobenzyl)-1,2-benzisoselenazol-3(*2H*)-one (4l).** White solid, yield 68%, mp 151-152°C. ^{1}H NMR (600 MHz, acetone-*d*$_6$) δ 7.98 (d, *J* = 7.9 Hz, 1H), 7.96 (dd, *J* = 7.9, 1.7 Hz, 1H), 7.65 (ddd, *J* = 8.3, 7.2, 1.7 Hz, 1H), 7.48 (ddd, *J* = 7.7, 7.4, 1.0 Hz, 1H), 7.38 (ddd, *J* = 11.5, 7.7, 2.2 Hz, 1H), 7.31 (ddd, *J* = 10.5, 8.3, 8.3 Hz, 1H), 7.25 (m, 1H), 4.99 (s, 2H). ^{13}C NMR (151 MHz, acetone-*d*$_6$) δ 167.65, 150.92 (dd, *J* = 246.1, 12.9 Hz), 150.49 (dd, *J* = 245.5, 12.5 Hz), 139.72, 137.03 (dd, *J* = 5.6, 4.0 Hz), 132.88, 128.99, 128.35, 126.97, 126.13, 125.72 (dd, *J* = 6.6, 3.1 Hz), 118.33 (d, *J* = 17.3 Hz), 118.05 (d, *J* = 18.0 Hz), 47.15. ^{19}F NMR (376 MHz, acetone-*d*$_6$) δ -139.56 (m), -141.43 (m). ^{77}Se NMR (76 MHz, acetone-*d*$_6$) δ 883.71. HRMS (ESI) *m/z* calculated for $C_{14}H_9F_2NOSe+H^+$ 325.9896, found 325.9904.

***N*-(3-Fluoro-4-(trifluoromethyl)benzyl)-1,2-benzisoselenazol-3(*2H*)-one (4m).** Yellow solid, yield 35%, mp 155-157°C. ^{1}H NMR (600 MHz, acetone-*d*$_6$) δ 8.01 (d, *J* = 8.2 Hz, 1H), 7.97 (dd, *J* = 7.7, 1.7 Hz, 1H), 7.74 (dd, *J* = 7.8, 7.8 Hz, 1H), 7.67 (ddd, *J* = 8.2, 7.2, 1.5 Hz, 1H), 7.49 (ddd, *J* = 7.7, 7.2, 1.0 Hz, 1H), 7.43 – 7.39 (m, 2H), 5.12 (s, 2H). ^{13}C NMR (101 MHz, acetone-*d*$_6$) δ 167.70, 160.29 (d, *J* = 254.4 Hz), 147.26 (d, *J* = 7.7 Hz), 139.67, 132.82, 128.84, 128.19 (d, *J* = 4.2 Hz), 127.85, 126.84, 126.03, 124.70 (d, *J* = 3.4 Hz), 123.56 (q, *J* = 271.0 Hz), 116.90 (d, *J* = 21.2 Hz), 46.96. ^{19}F NMR (376 MHz, acetone-*d*$_6$) δ -61.63 (d, *J* = 12.3 Hz), -116.30 (m). ^{77}Se NMR (76 MHz, acetone-*d*$_6$) δ 892.63. HRMS (ESI) *m/z* calculated for $C_{15}H_9F_4NOSe+H^+$ 375.9864, found 375.9859.

***N*-(4-Chloro-3-(trifluoromethyl)benzyl)-1,2-benzisoselenazol-3(*2H*)-one (4n).** White solid, yield 57%, mp 141-143°C. ^{1}H NMR (600 MHz, acetone-*d*$_6$) δ 8.00 (d, *J* = 8.1 Hz, 1H), 7.96 (dd, *J* = 7.8, 1.5 Hz, 1H), 7.88 (d, *J* = 2.3 Hz, 1H), 7.70 – 7.62 (m, 3H), 7.48 (ddd, *J* = 8.7, 7.3, 1.0 Hz, 1H), 5.10 (s, 2H). ^{13}C NMR (151 MHz, acetone-*d*$_6$) δ 167.80, 139.79, 139.46, 134.34, 132.96, 132.80, 131.47, 129.01, 128.53 (q, *J* = 30.9 Hz), 128.32 (q, *J* = 5.2 Hz), 128.19, 127.02, 126.20, 123.93 (q, *J* = 272.6 Hz), 47.05. ^{19}F NMR (376 MHz, acetone-*d*$_6$) δ -62.91. ^{77}Se NMR (76 MHz, acetone-*d*$_6$) δ 887.20. HRMS (ESI) *m/z* calculated for $C_{15}H_9ClF_3NOSe+H^+$ 391.9568, found 391.9570.

***N*-(4-Fluoro-3-(trifluoromethyl)benzyl)-1,2-benzisoselenazol-3(*2H*)-one (4o).** White solid, yield 39%, mp 154-156°C. ^{1}H NMR (600 MHz, acetone-*d*$_6$) δ 8.01 (d, *J* = 8.0 Hz, 1H), 7.97 (dd, *J* = 7.9, 1.7 Hz, 1H), 7.74 (dd, *J* = 7.9 Hz, 1H), 7.67 (ddd, *J* = 8.3, 7.2, 1.5 Hz, 1H), 7.49 (t, *J* = 7.5 Hz, 1H), 7.44 – 7.38 (m, 2H), 5.12 (s, 2H). ^{13}C NMR (101 MHz, acetone-*d*$_6$) δ 167.65, 160.31 (d, *J* = 254.5 Hz), 147.30 (d, *J* = 7.8 Hz), 139.64, 132.83, 128.85, 128.20 (d, *J* = 4.3 Hz), 127.87, 126.85, 126.03, 124.70 (d, *J* = 3.4 Hz), 123.58 (q, *J* = 271.2 Hz), 116.90 (d, *J* = 21.2 Hz), 46.96. ^{19}F NMR (376 MHz, acetone-*d*$_6$) δ -61.64 (d, *J* = 12.3 Hz, 3F), -116.31 (m, 1F). ^{77}Se NMR (76 MHz, acetone-*d*$_6$) δ 892.08. HRMS (ESI) *m/z* calculated for $C_{15}H_9F_4NOSe+H^+$ 375.9864, found 375.9859.

***N*-(3,5-Difluorobenzyl)-1,2-benzisoselenazol-3(*2H*)-one (4p).** White solid, yield 44%, mp 149-150°C. ^{1}H NMR (600 MHz, acetone-d_6) δ 8.00 (dd, *J* = 8.1, 1.0 Hz, 1H), 7.97 (dd, *J* = 7.8, 1.5 Hz, 1H), 7.66 (ddd, *J* = 8.1, 7.2, 1.5 Hz, 1H), 7.49 (ddd, *J* = 8.1, 7.2, 1.0 Hz, 1H), 7.05 (dd, *J* = 8.4, 2.4 Hz, 2H), 6.95 (dddd, *J* = 9.3, 9.3, 2.4, 2.4 Hz, 1H), 5.04 (s, 2H). ^{13}C NMR (151 MHz, acetone-d_6) δ 167.78, 164.02 (dd, *J* = 247.4, 12.5 Hz), 144.02 (t, *J* = 9.0 Hz), 139.78, 132.97, 129.04, 128.17, 127.01, 126.18, 111.77 (dd, *J* = 20.4, 5.2 Hz), 103.58 (t, *J* = 25.9 Hz), 47.30 (q, *J* = 2.7 Hz). ^{19}F NMR (376 MHz, acetone-d_6) δ -110.85 (m). ^{77}Se NMR (76 MHz, acetone-d_6) δ 889.75. HRMS (ESI) *m/z* calculated for $C_{14}H_9F_2NOSe+H^+$ 325.9896, found 325.9905.

***N*-(3,5-Bis(trifluoromethyl)benzyl)-1,2-benzisoselenazol-3(*2H*)-one (4q).** White solid, yield 31%, mp 218-220°C. ^{1}H NMR (600 MHz, acetone-d_6) δ 8.08 (s, 2H), 8.00 (d, *J* = 8.1 Hz, 1H), 7.98 (s, 1H), 7.97 (dd, *J* = 7.8, 1.5 Hz, 1H), 7.67 (ddd, *J* = 8.1, 7.2, 1.5 Hz, 1H), 7.50 (ddd, *J* = 7.9, 7.2, 1.0 Hz, 1H), 5.23 (s, 2H). ^{13}C NMR (151 MHz, acetone-d_6) δ 168.01, 142.91, 139.89, 133.08, 132.25 (q, *J* = 33.3 Hz), 129.67 (q, *J* = 4.2 Hz), 129.06, 128.04, 127.10, 124.42 (d, *J* = 272.6 Hz), 122.23 (p, *J* = 4.2 Hz), 47.24. ^{19}F NMR (376 MHz, acetone-d_6) δ -63.15. ^{77}Se NMR (76 MHz, acetone-d_6) δ 879.91. HRMS (ESI) *m/z* calculated for $C_{16}H_9F_6NOSe+H^+$ 425.9832, found 425.9841.

***N*-(2,3,4-Trifluorobenzyl)-1,2-benzisoselenazol-3(*2H*)-one (4r).** Yellow solid, yield 26%, mp 178-180°C. ^{1}H NMR (600 MHz, acetone-d_6) δ 8.00 (d, *J* = 8.1 Hz, 1H), 7.94 (dd, *J* = 7.8, 2.1 Hz, 1H), 7.65 (ddd, *J* = 8.3, 7.1, 1.5 Hz, 1H), 7.48 (ddd, *J* = 8.0, 7.3, 1.1 Hz, 1H), 7.27 (m, 1H), 7.19 (m, 1H), 5.07 (s, 2H). ^{13}C NMR (151 MHz, acetone-d_6) δ 167.65, 151.45 (ddd, *J* = 248.3, 9.9, 2.9 Hz), 150.19 (ddd, *J* = 249.0, 10.1, 3.3 Hz), 140.47 (dt, *J* = 249.4, 15.4 Hz), 139.70, 132.97, 128.97, 128.14, 127.03, 126.17, 125.44 (dt, *J* = 9.0, 4.5 Hz), 124.24 (dd, *J* = 12.0, 3.6 Hz), 113.37 (dd, *J* = 17.7, 3.8 Hz), 41.34 (t, *J* = 3.5 Hz). ^{19}F NMR (376 MHz, acetone-d_6) δ -137.06 (m), -140.06 (m), -162.99 (m). ^{77}Se NMR (76 MHz, acetone-d_6) δ 880.88 (d, *J* = 16.6 Hz). HRMS (ESI) *m/z* calculated for $C_{14}H_8F_3NOSe+H^+$ 343.9801, found 343.9797.

3. Biological studies

Microbial strains

Commercially available strains of *Sporosarcina pasteurii* CCM 2056 and *Helicobacter pylori* Tx30a (ATCC 51932) were used to study ureolytic activity in the presence of organoselenium compounds. *Helicobacter pylori* 2CLM belongs to the collection of the Department of Microbiology, Wrocław Medical University, Poland.

Purification of *S. pasteurii* urease

The native urease from the cells of *S. pasteurii* was induced and purified in a five-step chromatographic procedure, as previously described.[S5] The protein concentration was determined using the Bradford assay and the kinetic parameters were: $K_M = 14.09 \pm 1.03$ mM, $v_{max} = 1.49 \pm 0.08$ µM/s, as calculated with the use of the GraphPad Prism 5 software.

Enzymatic activity assays

Purified urease activity with the addition of the organoselenium compounds studied was measured using cresol red as an indicator of pH changes caused by ammonia produced during ureolysis.[S6] The reaction was carried out in 3 mM phosphate buffer containing 30 mM urea, 0.01 % w/v cresol red, purified urease (at a concentration that led to a change in A_{570} of approximately 1.5 in 90 min) and studied compounds at various concentrations. The reaction was followed by using a TECAN Sunrise spectrometer, and the resulting progress curves were further analyzed to determine the activity and binding mechanism. The reaction was followed in a system without preincubation of the enzyme with the benzisoselenazolone derivatives (addition of urease initiated the measurement).

K_i value calculations

As the progress curves of the inhibited reactions with no enzyme-inhibitor preincubation were nonlinear and the initial velocity was independent of the concentration of the studied compounds, they were determined to be slow-binding inhibitors that work in accordance with the single-step binding mechanism. The progress curves were fitted to the following equation to calculate the apparent reaction rate constants (k_{app}):

$$P = v_s * t + \frac{v_0 - v_s}{k_{app}} * \left(1 - e^{-k_{app} * t}\right)$$

P – product concentration; t – time; v_0 – initial state velocity; v_s – steady state velocity.
The k_{app} values were then plotted against the inhibitor concentration [I], and the resulting linear function was used to calculate the dissociation rate of the EI complex (K_i):

$$k_{app} = k_{-3} + \frac{k_3}{1 + \frac{[S]}{K_M}} * [I]$$

$$K_i = \frac{k_{-3}}{k_3}$$

Whole cell ureolysis assays

To measure the inhibitory activity of the compounds studied against whole cells of ureolytic pathogens, the cresol red reaction was carried out using *H. pylori* cells that were washed twice with 10 mM PBS and used as a biocatalyst.[S6] The reaction mixture contained 10 mM PBS, 10

mM urea, bacterial cells, and urease inhibitors. The results were then used to calculate the IC_{50} values of the organoselenium compounds against the whole cell ureolysis of *H. pylori*.

Combination of drugs against *H. pylori* 2CML

A multidrug-resistant clinical isolate *H. pylori* 2CML from the collection of the Department of Microbiology, Wrocław Medical University)[S7] was used to assess the antimicrobial activity of compounds **1** and **4m**, in combination with Clarithromycin, Metronidazole, and Levofloxacin (Sigma-Aldrich). The experiments were carried out on flat-bottom 12-well microtiter plates (Bionovo, Legnica, Poland), arranged in a 48-well panel to use the checkboard method.[S8] Concentration gradients of benzisoselenazolones **1** or **4m** (from 2x MIC to 1/16x MIC) versus the selected antibiotic were placed in external wells along the x- and y-axes, respectively. The remaining wells contained varying concentrations of both compounds. Each well consisted of 1 mL of Brain Heart Infusion (Oxoid, Le Pont de Claix, France) broth with 5% foetal calf serum (Gibco, Paisley, Scotland, UK), a bacterial suspension of 10^7 CFU/mL and the desired concentration of the antimicrobial(s) tested. Following the setup, the plates were incubated for 3 days at 37 °C under microaerophilic conditions (GENbox microaer kits, BioMerrieux, Marcy l'Etoile, France), shaking at 100 rpm.

The minimum inhibitory concentration (MIC) was determined as the lowest concentration in which no microbial growth was observed.

The interactions between antimicrobials were interpreted by calculation of the fractional inhibitory concentration (FIC) index (FICI), defined as FICI = $FIC_A + FIC_B$ (FIC_A = MIC of substance A in combination / MIC of substance A alone, FIC_B = MIC of substance B in combination / MIC of substance B alone). FICI ≤0.5 was considered as synergism; >0.5 but ≤1 as additivity, while >1 and as neutral.

4. Molecular modeling

The crystal structures of *S. pasteurii* urease with catechol bound to Cys322 (1.50 Å resolution, PDB id 5G4H)[S10] or *H. pylori* urease complexed with acetohydroxamic acid (3.00 Å resolution, PDB id 1E9Y)[S11] were used as the starting point for the calculations. Calculations were performed using CHARMM (v. 45b2).[S12] The structure was prepared using Discovery Studio Viewer 2022 (Dassault Systemes Biovia Corp) in the following steps: (a) hydrogen atoms were automatically added assuming a pH of 7.0, (b) the protonation of amino acid residues that form active sites was manually checked and adjusted, and (c) the partial charges of all atoms were assigned using the Momany-Rone algorithm. Minimization of the inhibitor-enzyme covalent complex was performed using the CHARMM force field with the conjugate gradient minimization. Minimization was performed up to a total energy change of 0.0 or an average gradient of 0.1. Residues that did not form the active site cleft were frozen. No implicit solvent model was applied. The nonbond radius was set to 14 Å.

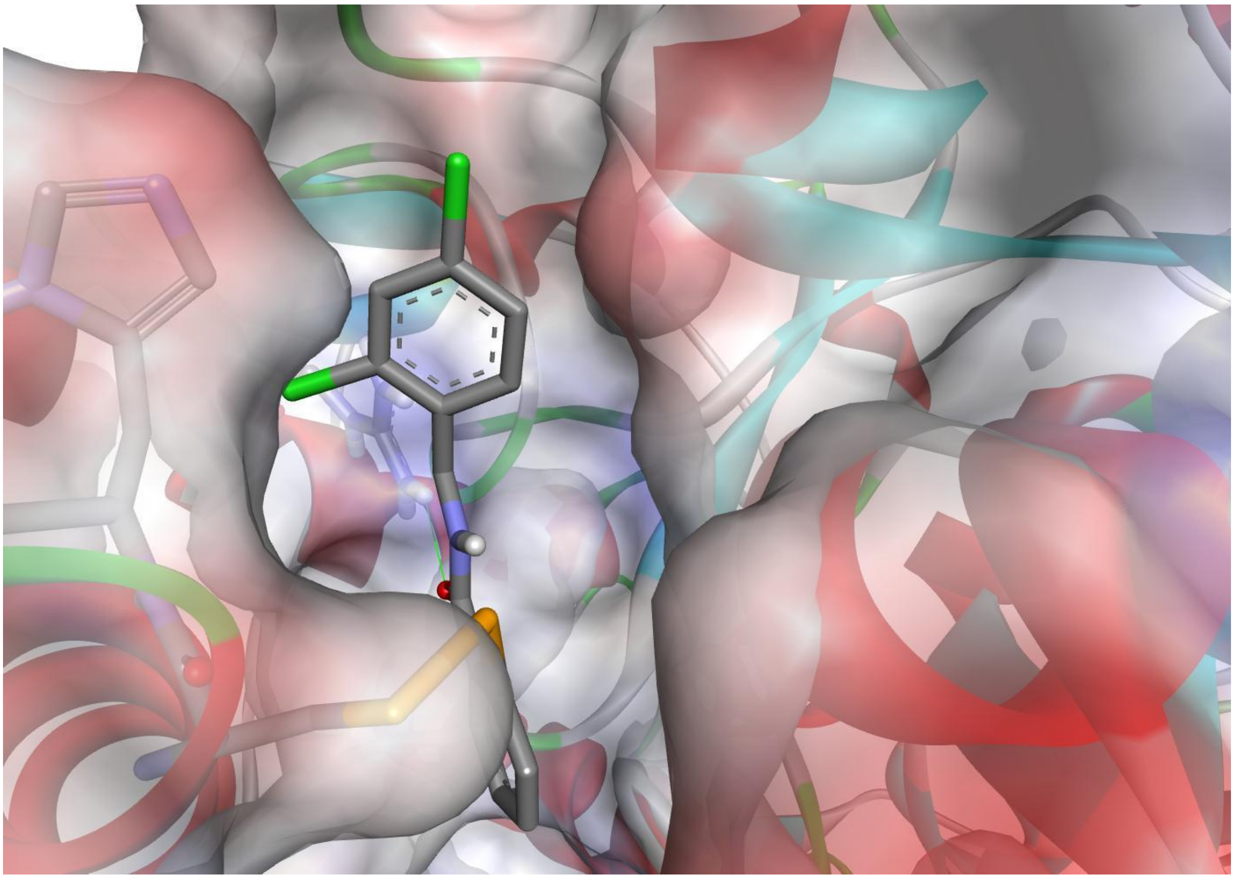

Figure S1. Modeled structure of *S. pasteurii* urease (PDB id 5G4H)[S10] with inhibitor **4f** bound to the Cys322 residue of the enzyme. The inhibitor is shown as sticks colored according to the atom type (gray, carbon; blue, nitrogen; white, hydrogen; red, oxygen; yellow, sulfur; orange, selenium; green, chlorine). The protein is shown as a ribbon, with the solvent accessible surface colored according to the interpolated charge. The hydrogen bond is shown as a thin solid green line.

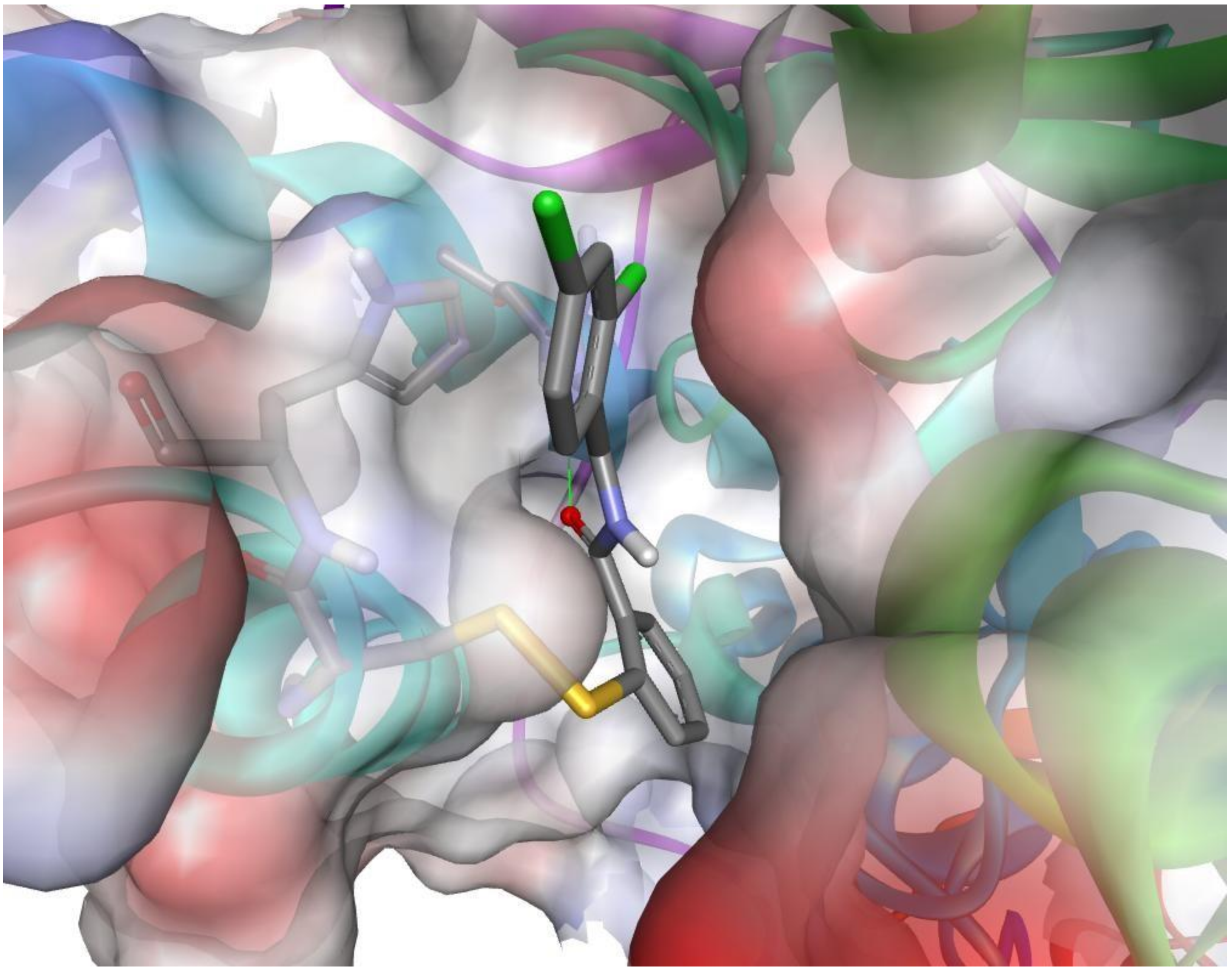

Figure S2. Modeled structure of *H. pylori* urease (PDB id 1E9Y)[S11] with inhibitor **4f** bound to the Cys321 residue of the enzyme. The inhibitor is shown as sticks colored according to the atom type (gray, carbon; blue, nitrogen; white, hydrogen; red, oxygen; yellow, sulfur; orange, selenium; green, chlorine). The protein is shown as a ribbon, with the solvent accessible surface colored according to the interpolated charge. The hydrogen bond is shown as a thin solid green line.

5. References

(S1) Młochowski, J.; Kloc, K.; Syper, L.; Inglot, A. D.; Piasecki, E. Aromatic and Azaaromatic Diselenides, Benzisoselenazolones and Related Compounds as Immunomodulators Active in Humans: Synthesis and Properties. *Liebigs Ann. Chem.* **1993**, (12), 1239–1244.

(S2) Pietka-Ottlik, M.; Wójtowicz-Młochowska, H.; Kołodziejczyk, K.; Piasecki, E.; Młochowski, J. New Organoselenium Compounds Active Against Pathogenic Bacteria, Fungi and Viruses. *Chem. Pharm. Bull.* **2008**, *56* (10), 1423–1427.

(S3) Balkrishna, S. J.; Bhakuni, B. S.; Chopra, D.; Kumar, S. Cu-Catalyzed Efficient Synthetic Methodology for Ebselen and Related Se−N Heterocycles. *Org. Lett.* **2010**, *12* (23), 5394–5397.

(S4) Węglarz-Tomczak, E.; Burda-Grabowska, M.; Giurg, M. Mucha, A. Identification of Methionine Aminopeptidase 2 as a Molecular Target of the Organoselenium Drug Ebselen and Its Derivatives/Analogues: Synthesis, Inhibitory Activity and Molecular Modeling Study. Bioorg. Med. Chem. Lett. 2016, *26* (21), 5254–5259.

(S5) Macegoniuk, K.; Dziełak, A.; Mucha, A.; Berlicki, Ł. Bis(aminomethyl)phosphinic Acid, a Highly Promising Scaffold for the Development of Bacterial Urease Inhibitors. *ACS Med. Chem. Lett.* **2014**, *6*, 146–150.

(S6) Maślanka, M.; Tabor, W.; Krzyżek, P.; Grabowiecka, A.; Berlicki, Ł.; Mucha, A. Inhibitory Activity of Catecholic Phosphonic and Phosphinic Acids Against *Helicobacter pylori* Ureolysis. Eur. J. Med. Chem. **2023**, *257*, 115528.

(S7) Krzyżek, P.; Migdał, P.; Grande, R.; Gościniak, G. Biofilm Formation of *Helicobacter pylori* in Both Static and Microfluidic Conditions Is Associated With Resistance to Clarithromycin. *Front. Cell. Infect. Microbiol.* **2022**, *12*, 868905.

(S8) Krzyżek, P.; Migdał, P.; Paluch, E.; Karwańska, M.; Wieliczko, A.; Gościniak, G. Myricetin as an Antivirulence Compound Interfering with a Morphological Transformation into Coccoid Forms and Potentiating Activity of Antibiotics against *Helicobacter pylori*. *Int. J. Mol. Sci.* **2021**, *22* (5), 2695.

(S9) Krzyżek, P.; Paluch, E.; Gościniak, G. Synergistic Therapies as a Promising Option for the Treatment of Antibiotic-Resistant *Helicobacter pylori*. *Antibiotics* **2020**, *9* (10), 658.

(S10) Mazzei, L.; Cianci, M.; Musiani, F.; Lente, G.; Palombo, M.; Ciurli, S. Inactivation of Urease by Catechol: Kinetics and Structure. *J. Inorg. Biochem.* **2017**, *166*, 182–189.

(S11) Ha, N. C.; Oh, S. T.; Sung, J.; Cha, K. A.; Lee, M. H.; Oh, B. H. Supramolecular Assembly and Acid Resistance of *Helicobacter pylori* Urease. *Nat. Struct. Mol. Biol.* **2001**, *8* (6), 505–509.

(S12) Hwang, W.; Austin, S. L.; Blondel, A.; Boittier, E. D.; Boresch, S.; Buck, M.; Buckner, J.; Caflisch, A.; Chang, H. T.; Cheng, X.; Choi, Y. K.; Chu, J. W.; Crowley, M. F.; Cui, Q.; Damjanovic, A.; Deng, Y.; Devereux, M.; Ding, X.; Feig, M. F.; Gao, J.; Glowacki, D. R.; Gonzales, 2nd, J. E.; Hamaneh, M. B.; Harder, E. D.; Hayes, R. L.; Huang, J.; Huang, Y.; Hudson, P. S.; Im, W.; Islam, S. M.; Jiang, W.; Jones, M. R.; Käser, S.; Kearns, F. L.; Kern, N. R.; Klauda, J. B.; Lazaridis, T.; Lee, J.; Lemkul, J. A.; Liu, X.; Luo, Y.; MacKerell, Jr, A. D.; Major, D. T.; Meuwly, M.; Nam, K.; Nilsson, L.; Ovchinnikov, V.; Paci, E.; Park, S.; Pastor, R. W.; Pittman, A. R.; Post, C. B.; Prasad, S.; Pu, J.; Qi, Y.; Rathinavelan, T.; Roe, D. R.; Roux, B.; Rowley, C. N.; Shen, J.; Simmonett, A. C.; Sodt, A. J.; Töpfer, K.; Upadhyay, M.; van der Vaart, A.; Vazquez-Salazar, L. I.; Venable, R. M.; Warrensford, L. C.; Woodcock, H. L.; Wu, Y.; Brooks, 3rd, C. L.; Brooks, B. R.; Karplus, M. CHARMM at 45: Enhancements in Accessibility, Functionality, and Speed. *J. Phys. Chem. B.* **2024**, *128* (41), 9976–10042.

6. NMR spectra and HPLC analyses

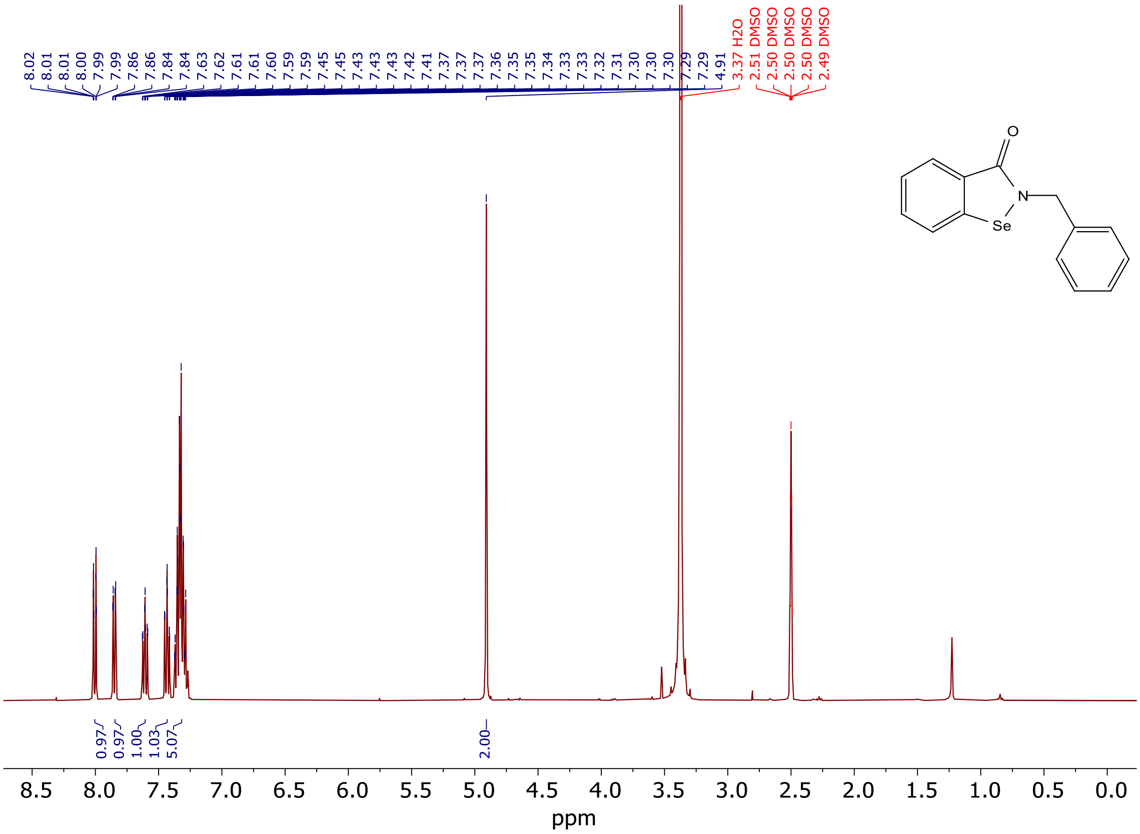

Figure S3. ^{1}H NMR spectrum of compound **3**.

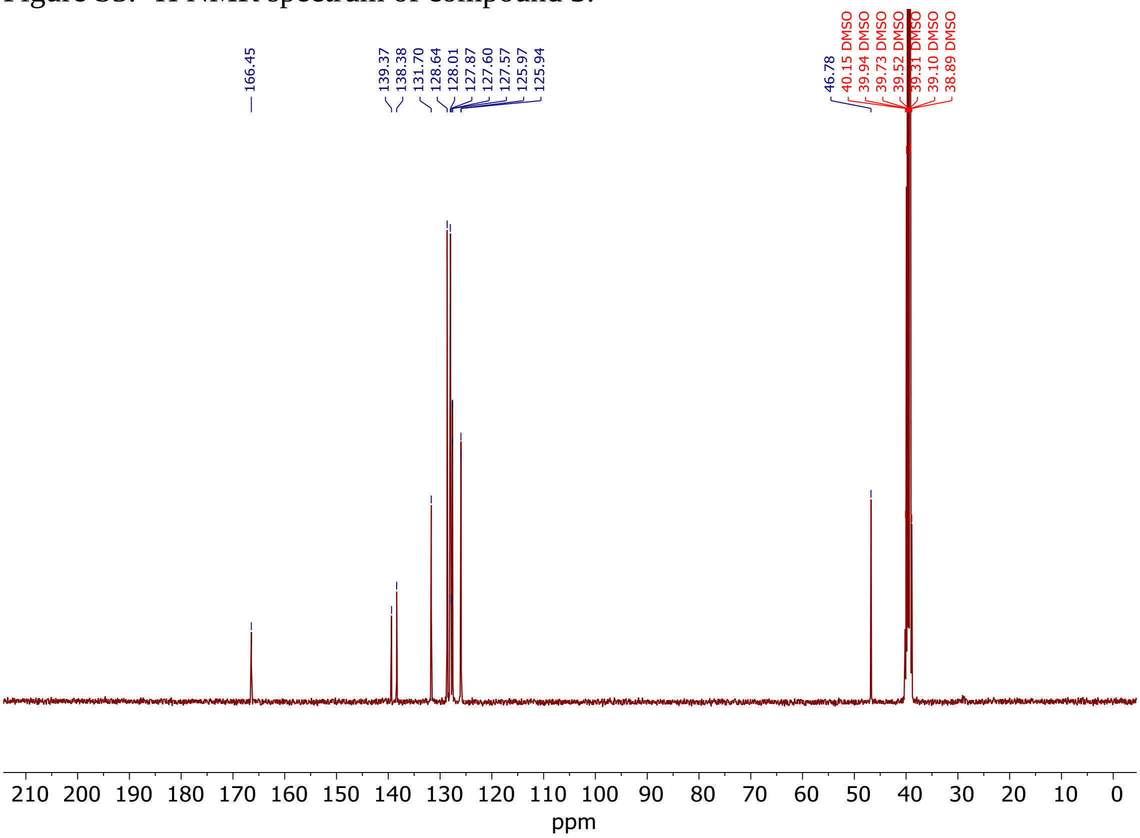

Figure S4. ^{13}C NMR spectrum of compound **3**.

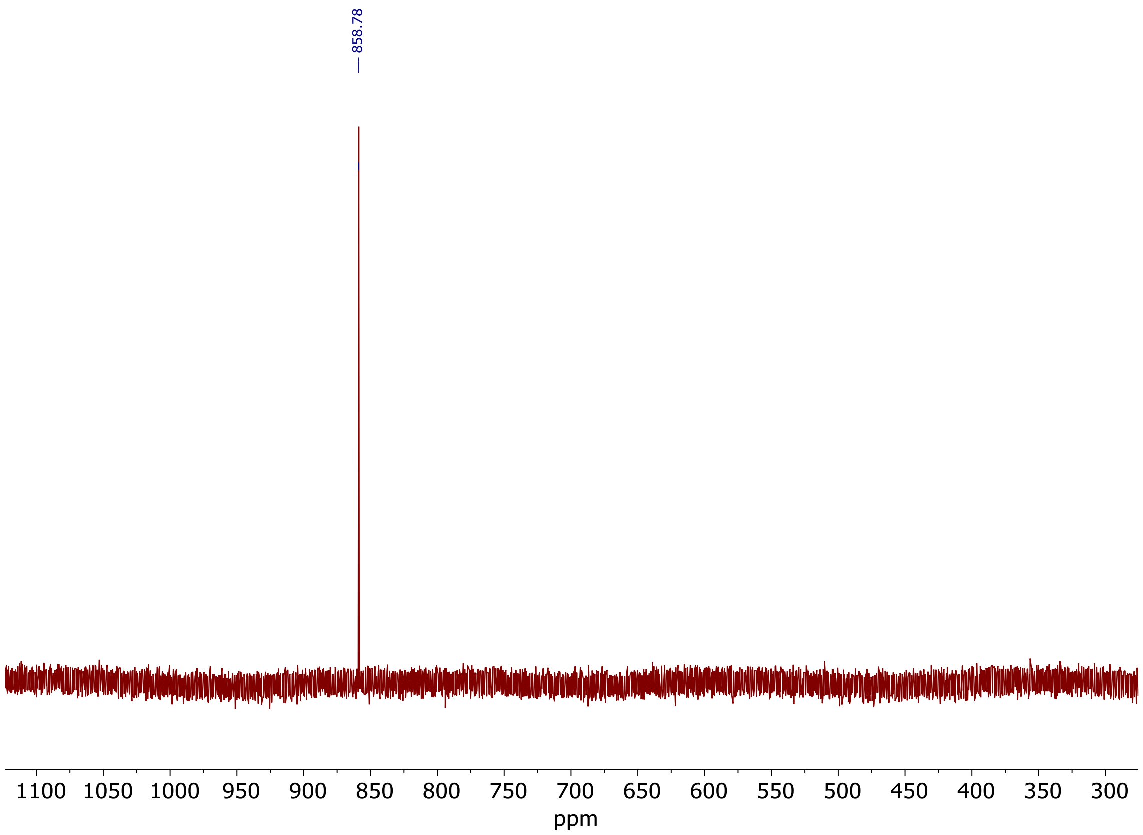

Figure S5. ^{77}Se NMR spectrum of compound **3**.

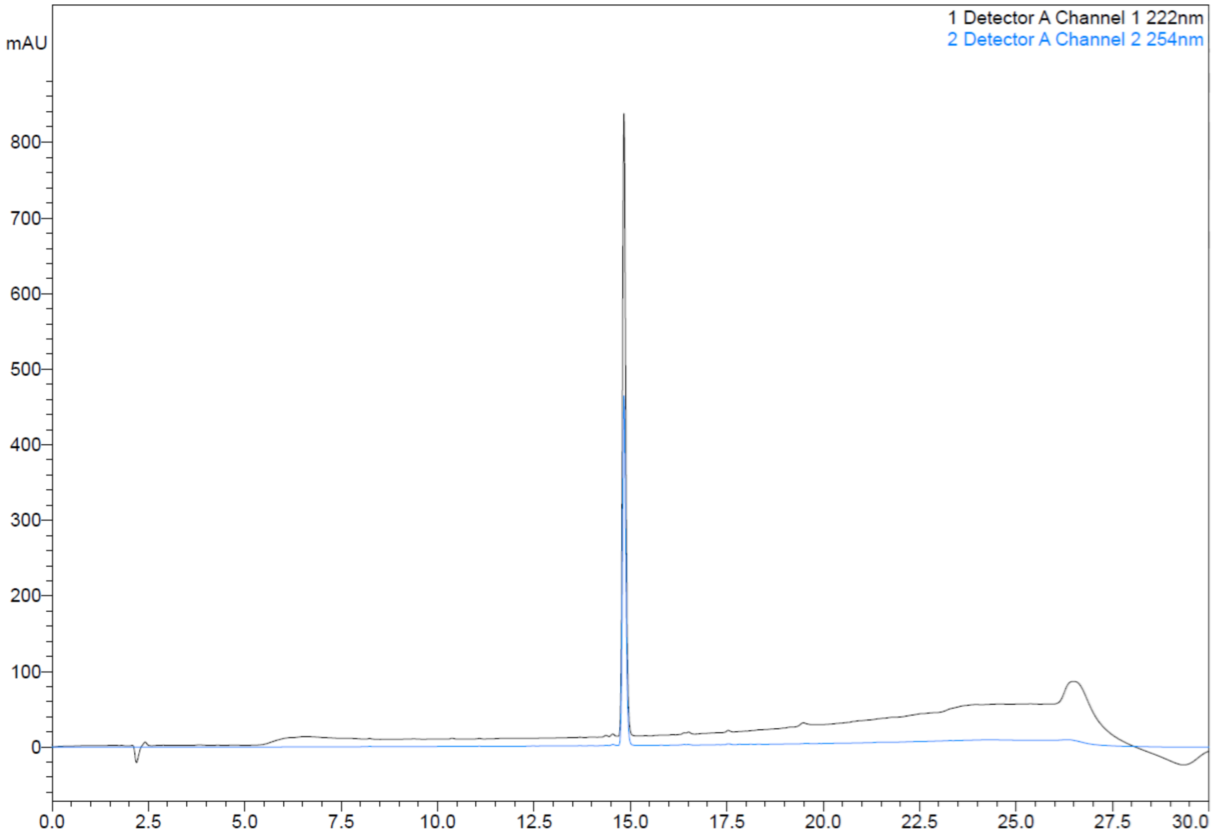

Figure S6. Analytical HPLC analysis of compound **3**.

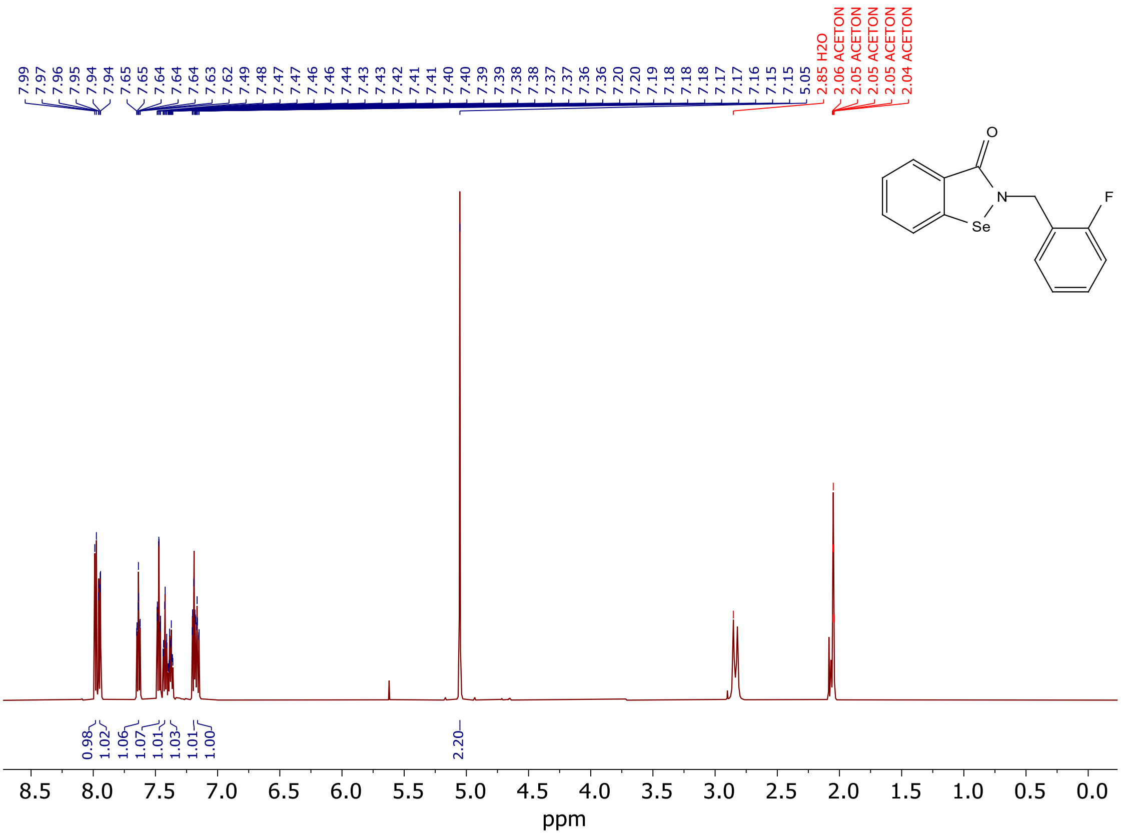

Figure S7. ^{1}H NMR spectrum of compound **4a**.

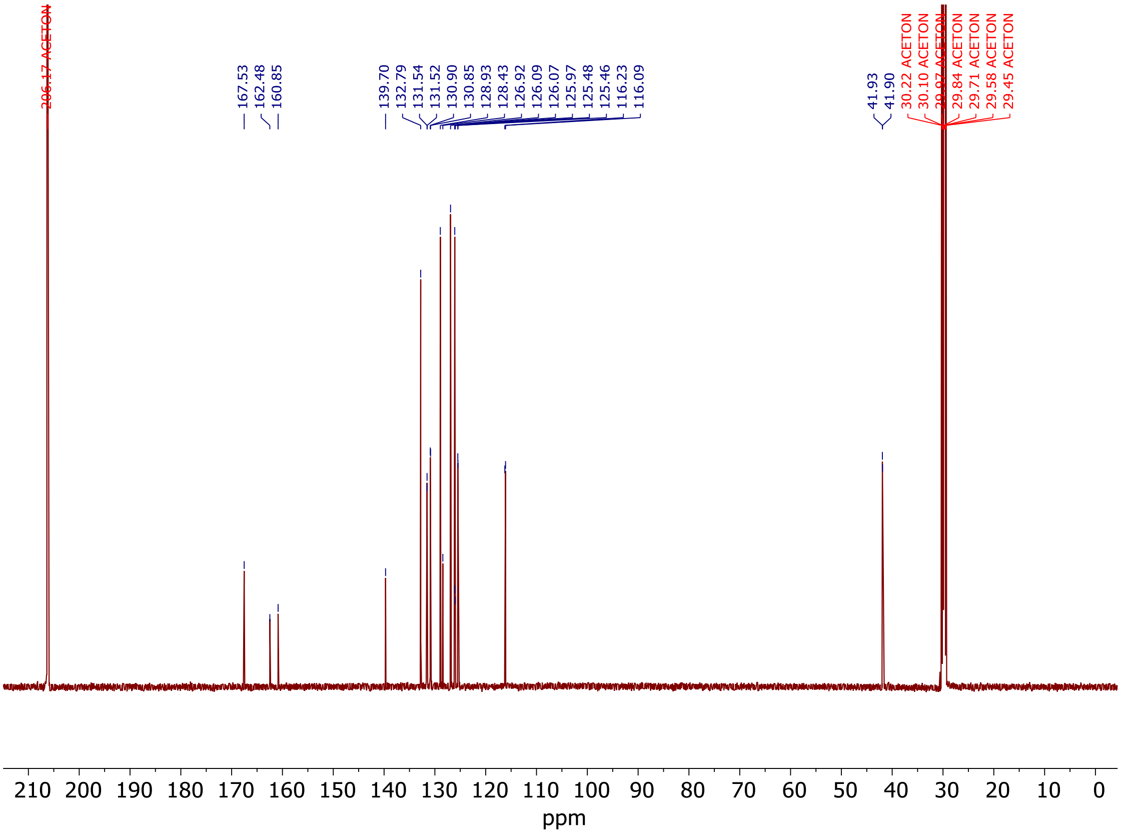

Figure S8. ^{13}C NMR spectrum of compound **4a**.

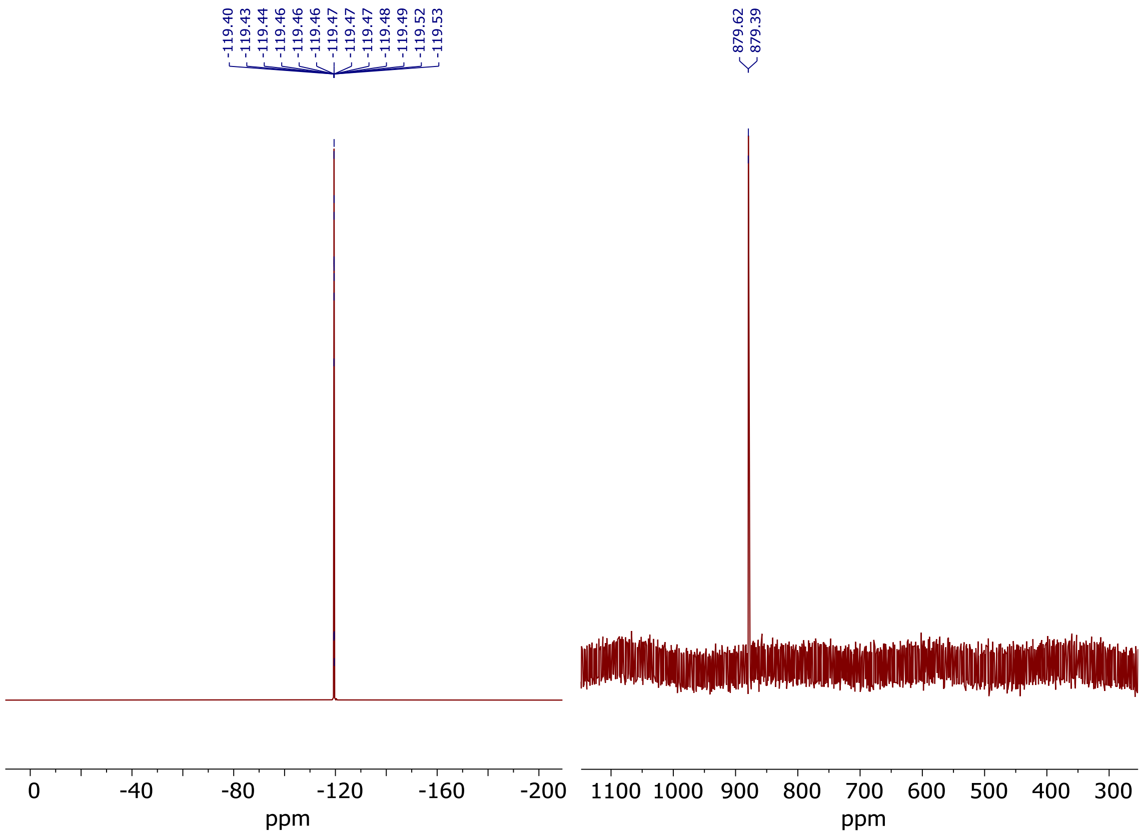

Figure S9. ^{19}F and ^{77}Se NMR spectra of compound **4a**.

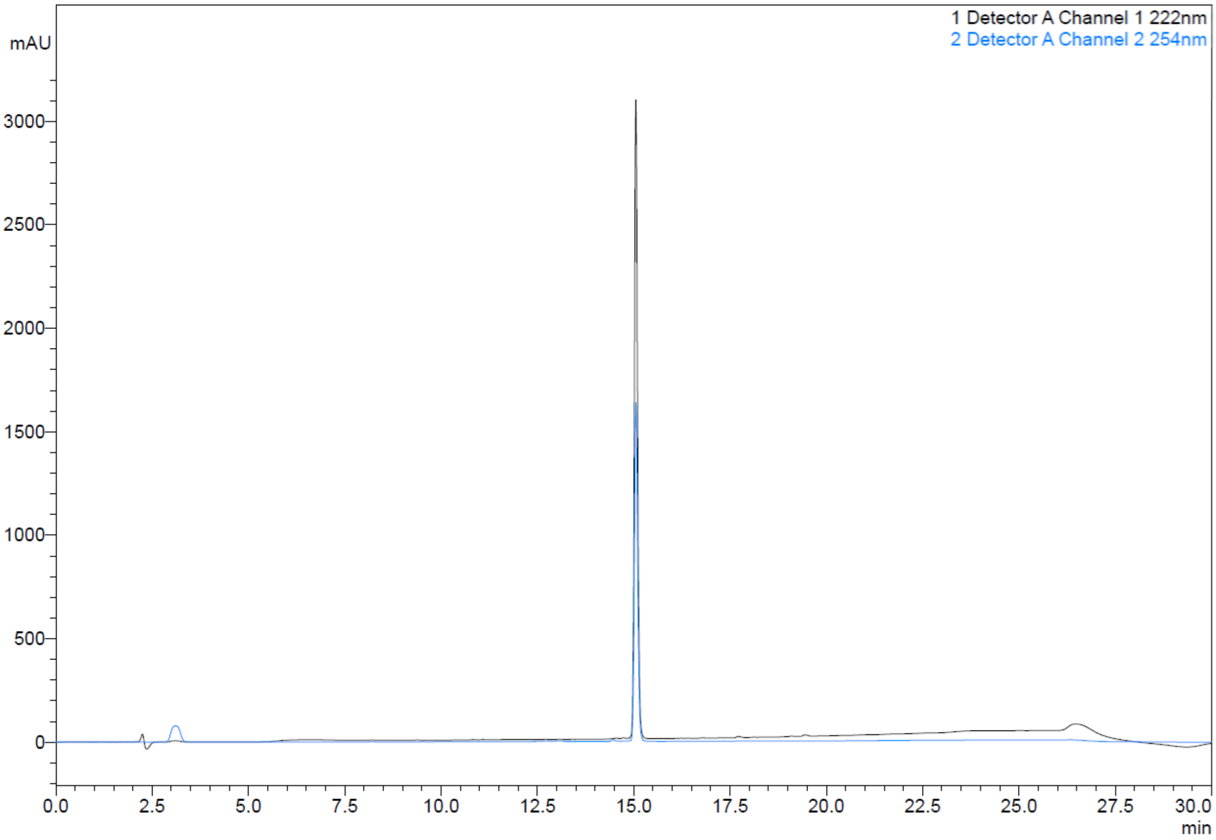

Figure S10. Analytical HPLC analysis of compound **4a**.

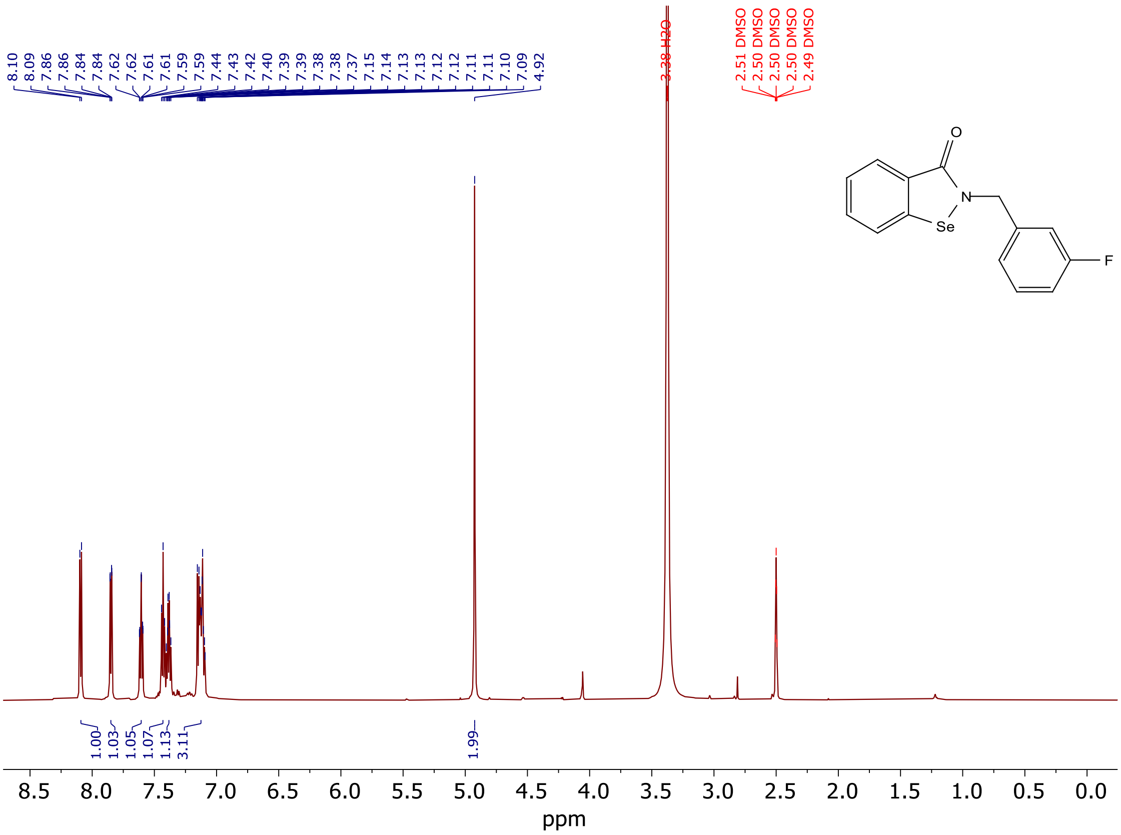

Figure S11. ^{1}H NMR spectrum of compound **4b**.

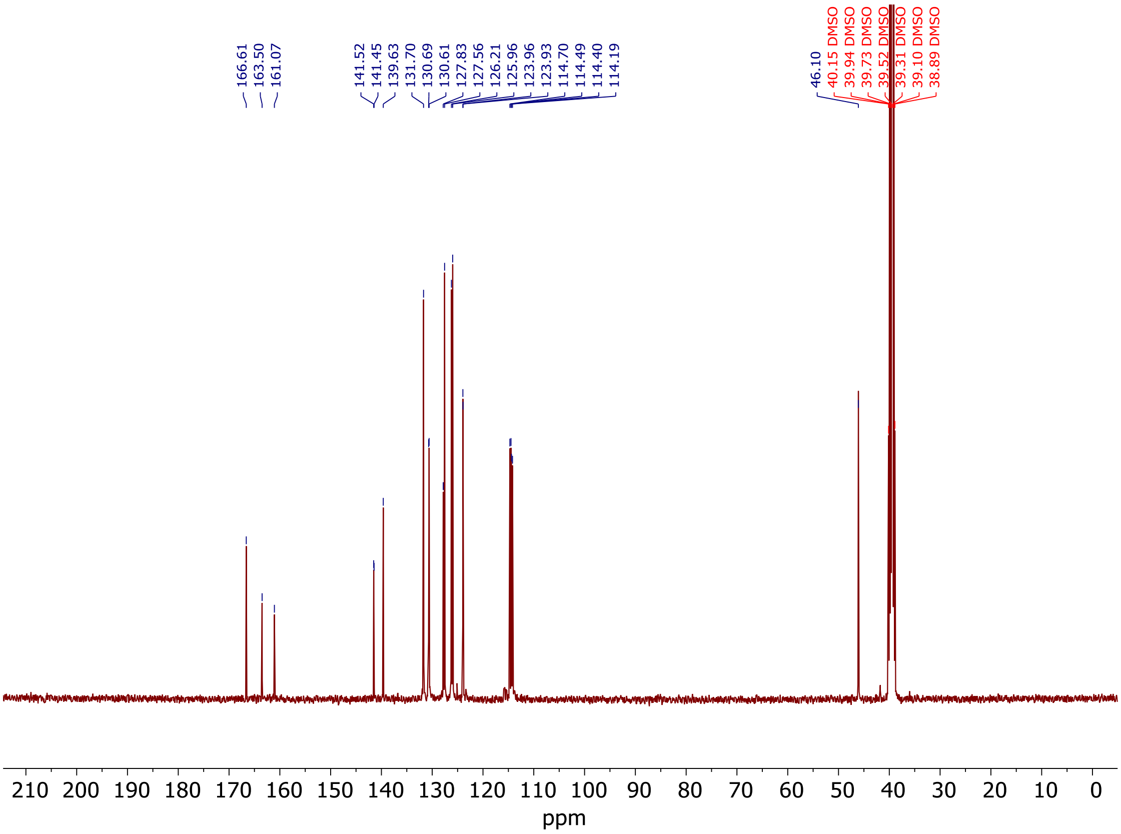

Figure S12. ^{13}C NMR spectrum of compound **4b**.

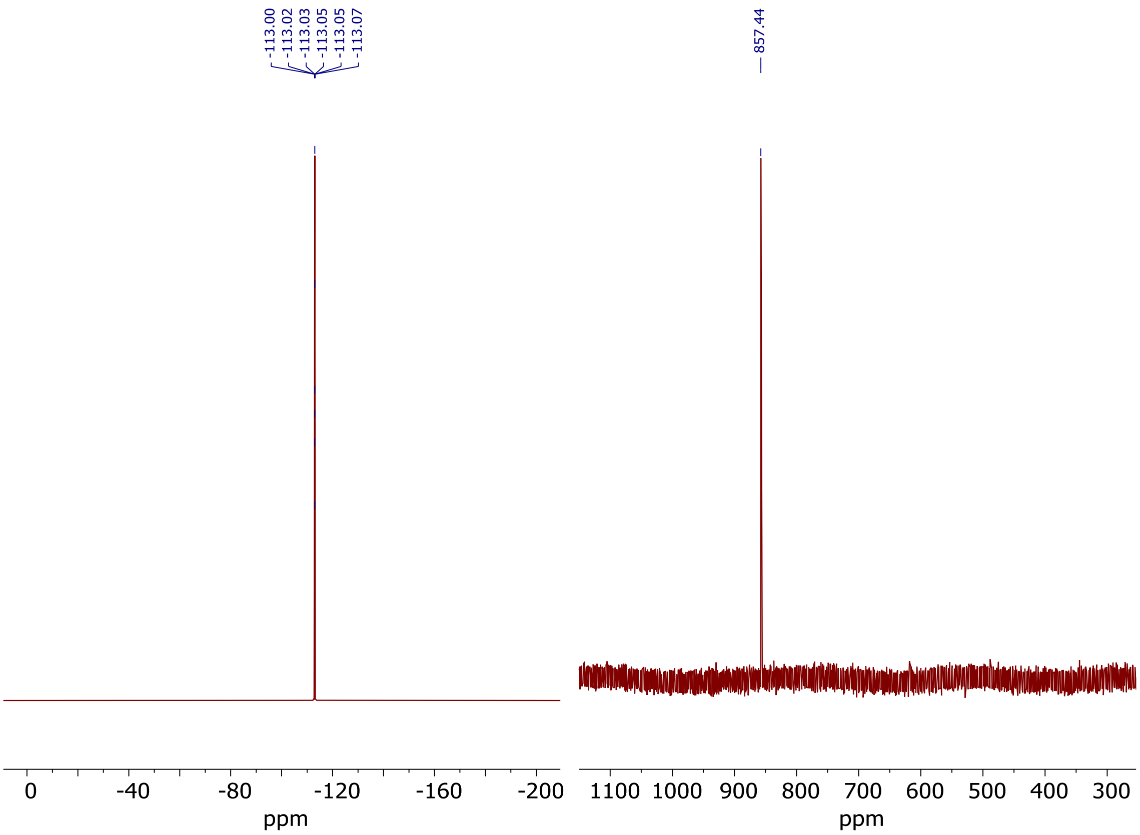

Figure S13. ^{19}F and ^{77}Se NMR spectra of compound **4b**.

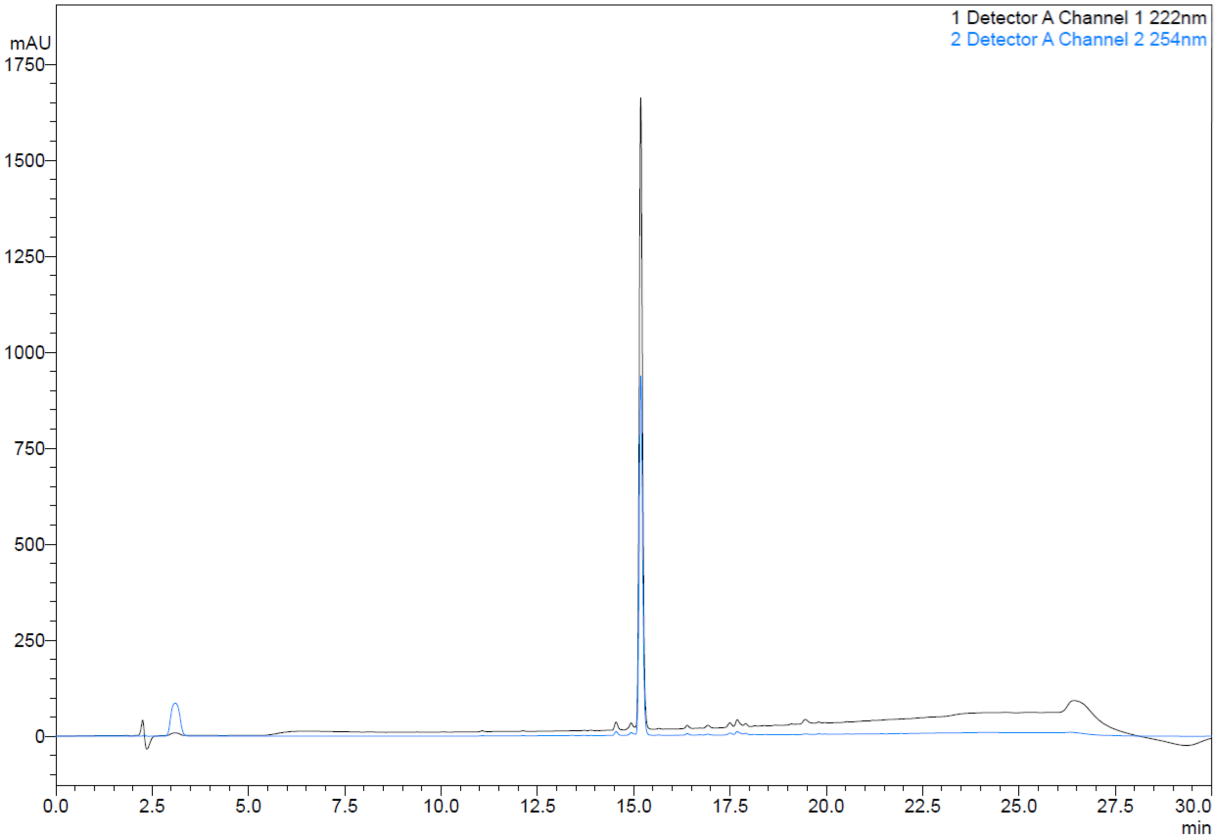

Figure S14. Analytical HPLC analysis of compound **4b**.

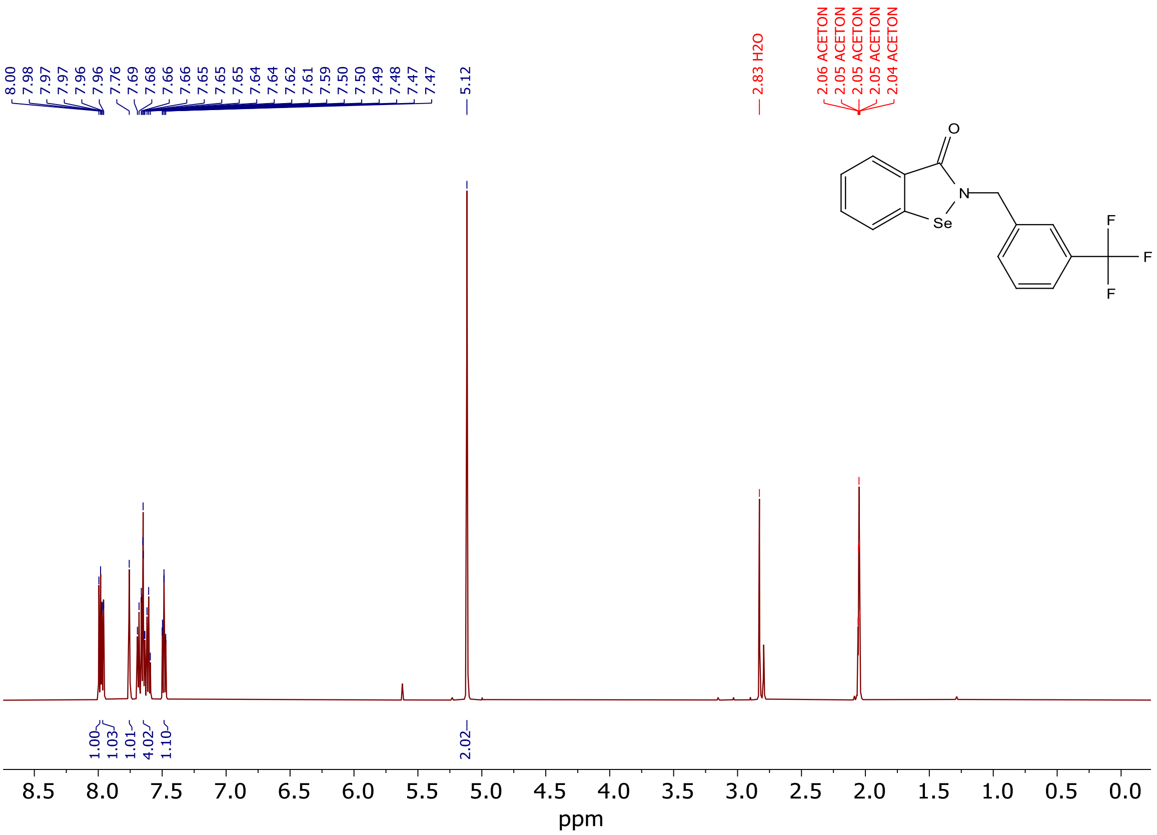

Figure S15. ^{1}H NMR spectrum of compound **4c**.

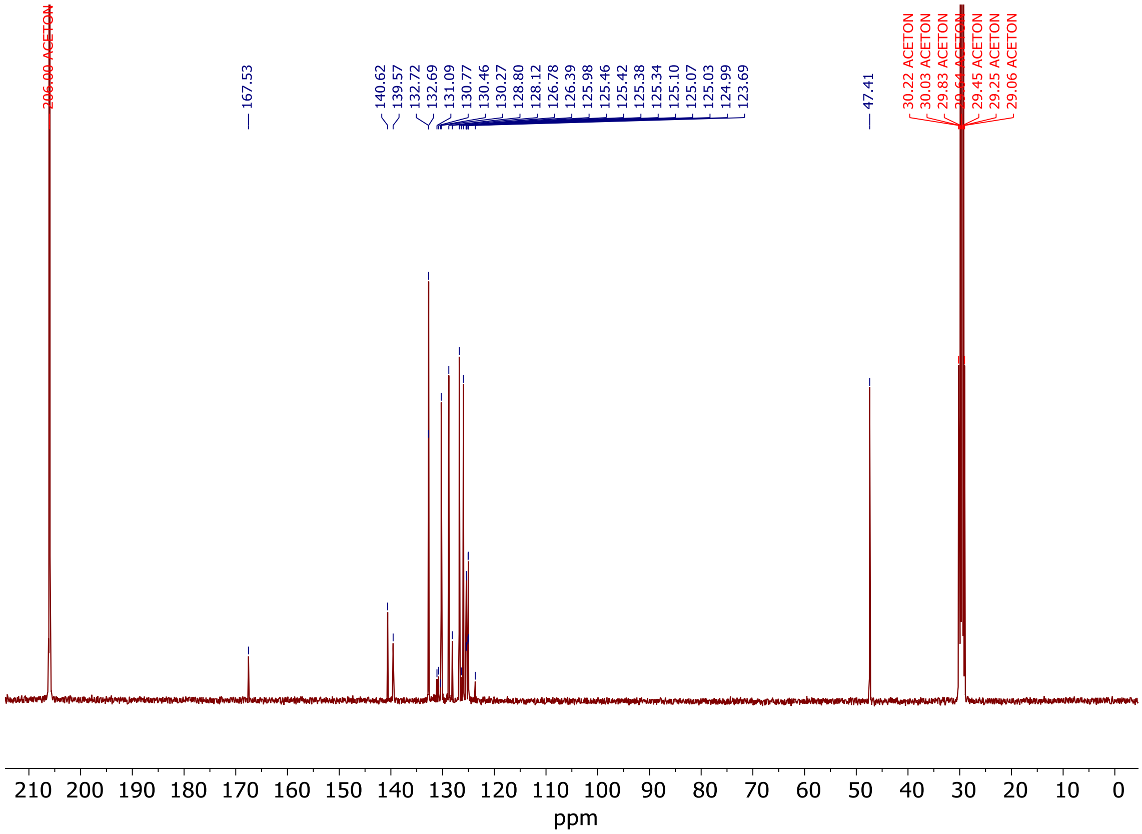

Figure S16. ^{13}C NMR spectrum of compound **4c**.

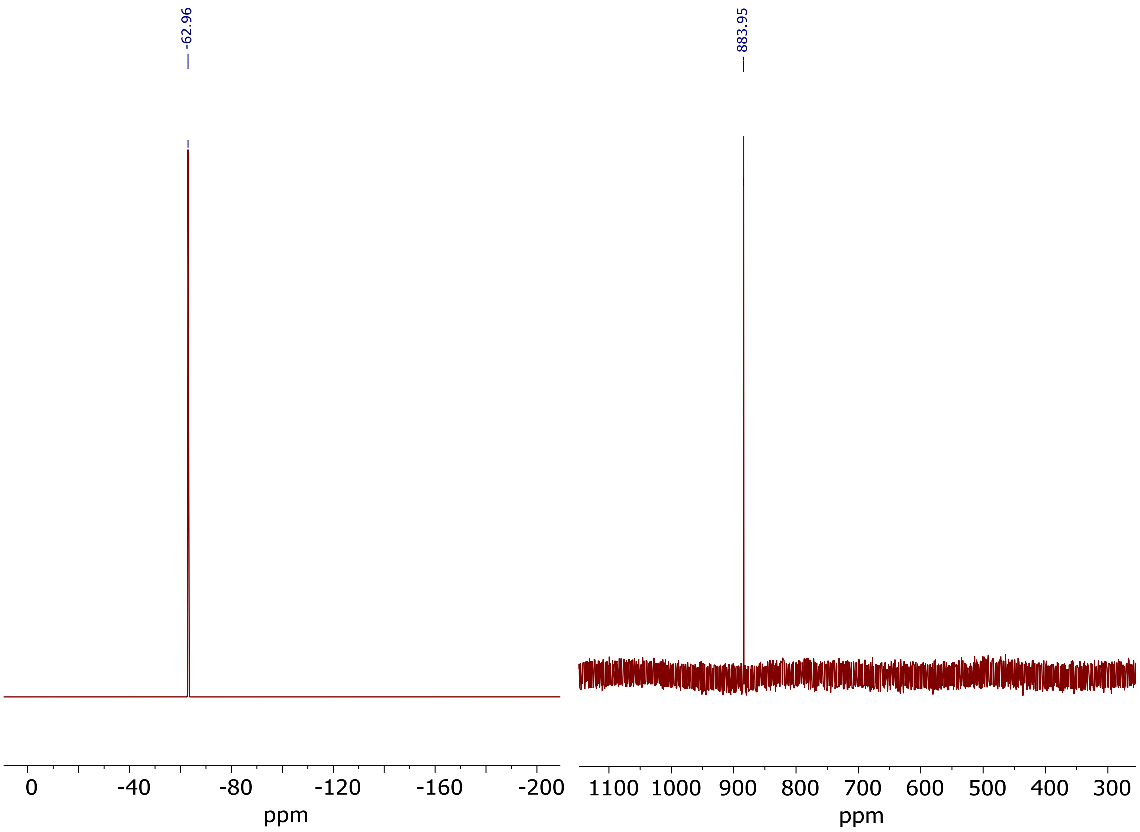

Figure S17. ^{19}F and ^{77}Se NMR spectra of compound **4c**.

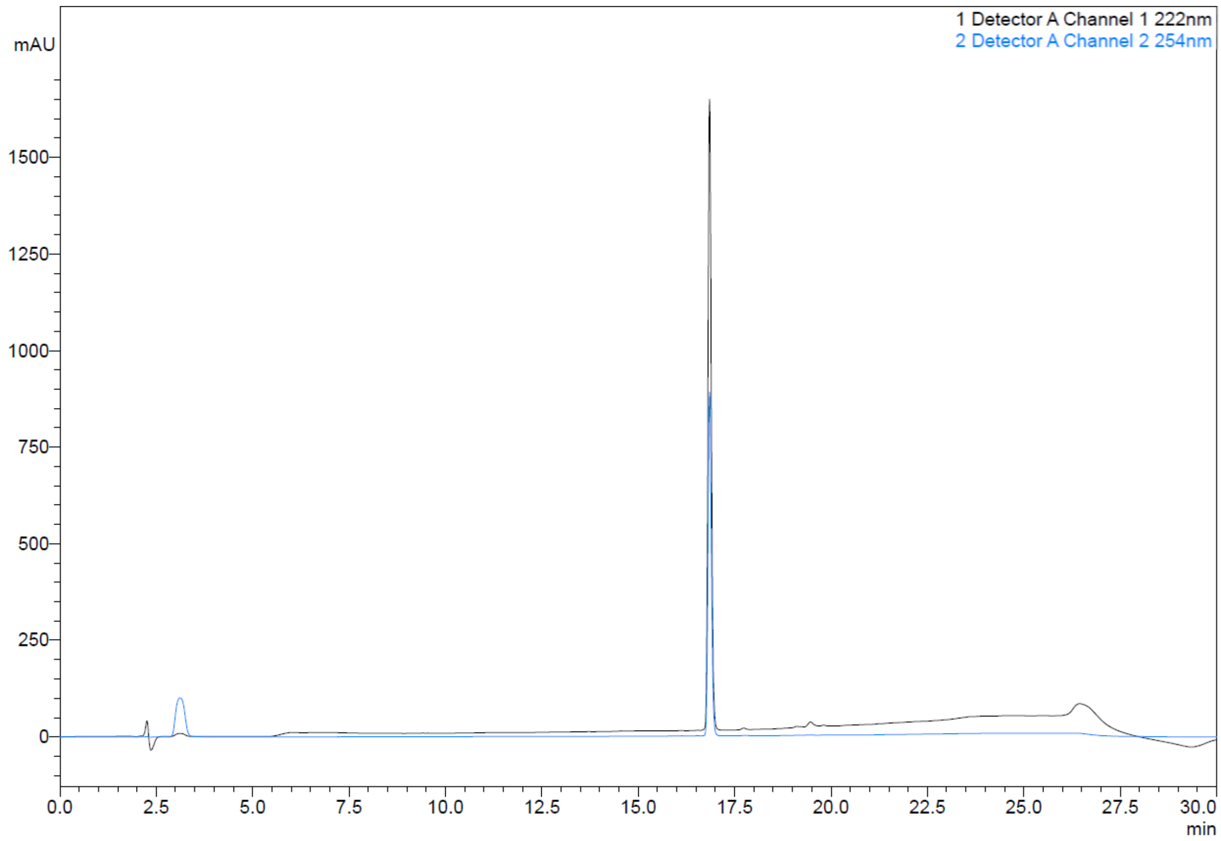

Figure S18. Analytical HPLC analysis of compound **4c**.

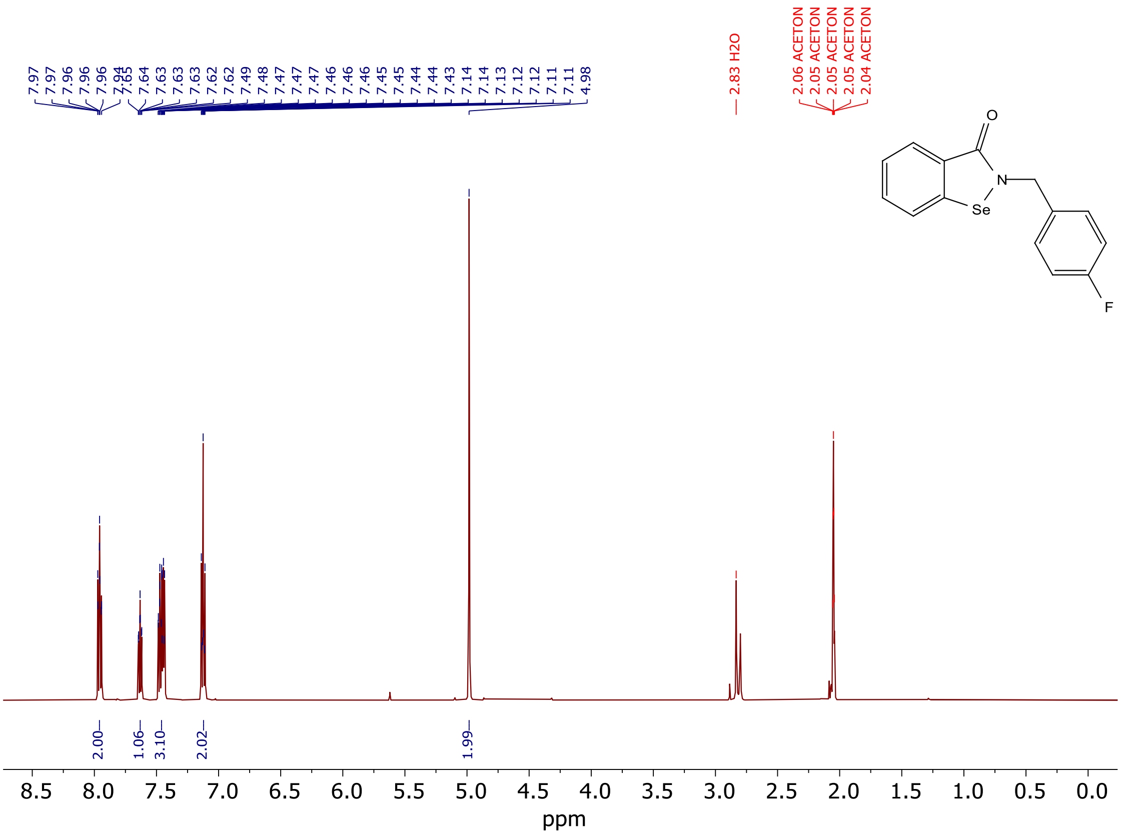

Figure S19. ^{1}H NMR spectrum of compound **4d**.

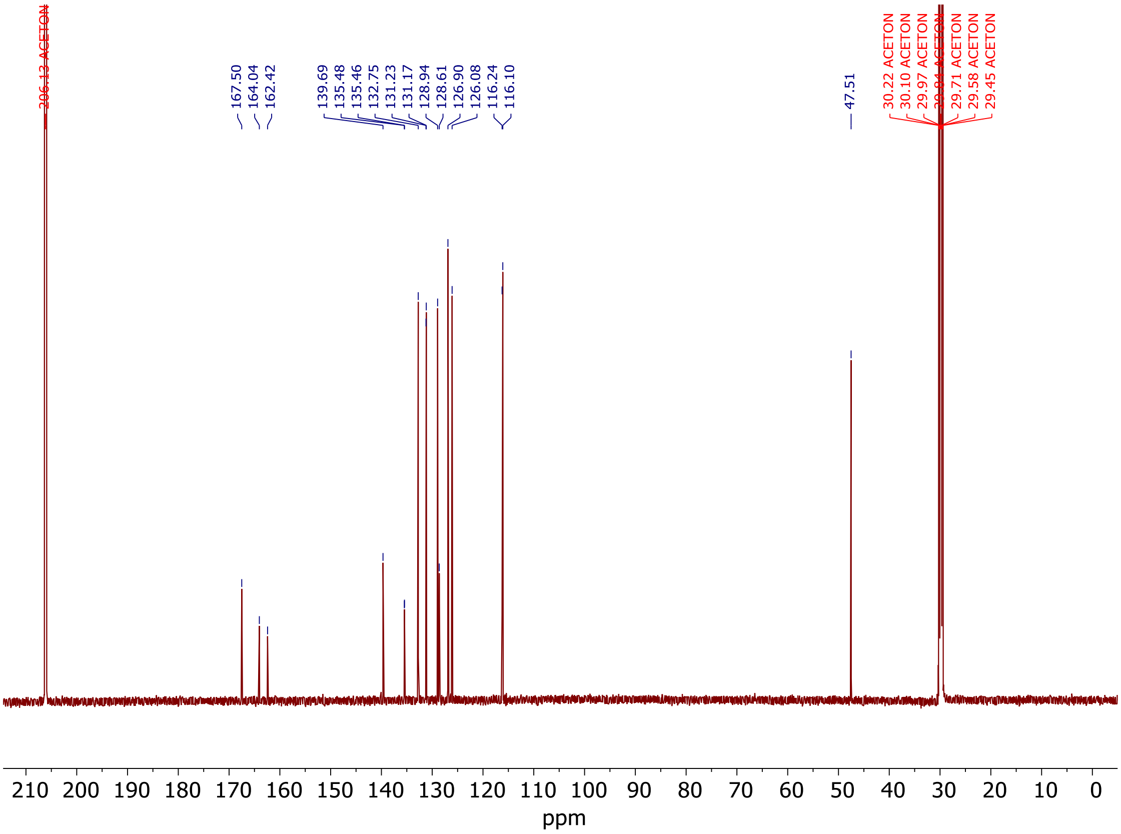

Figure S20. ^{13}C NMR spectrum of compound **4d**.

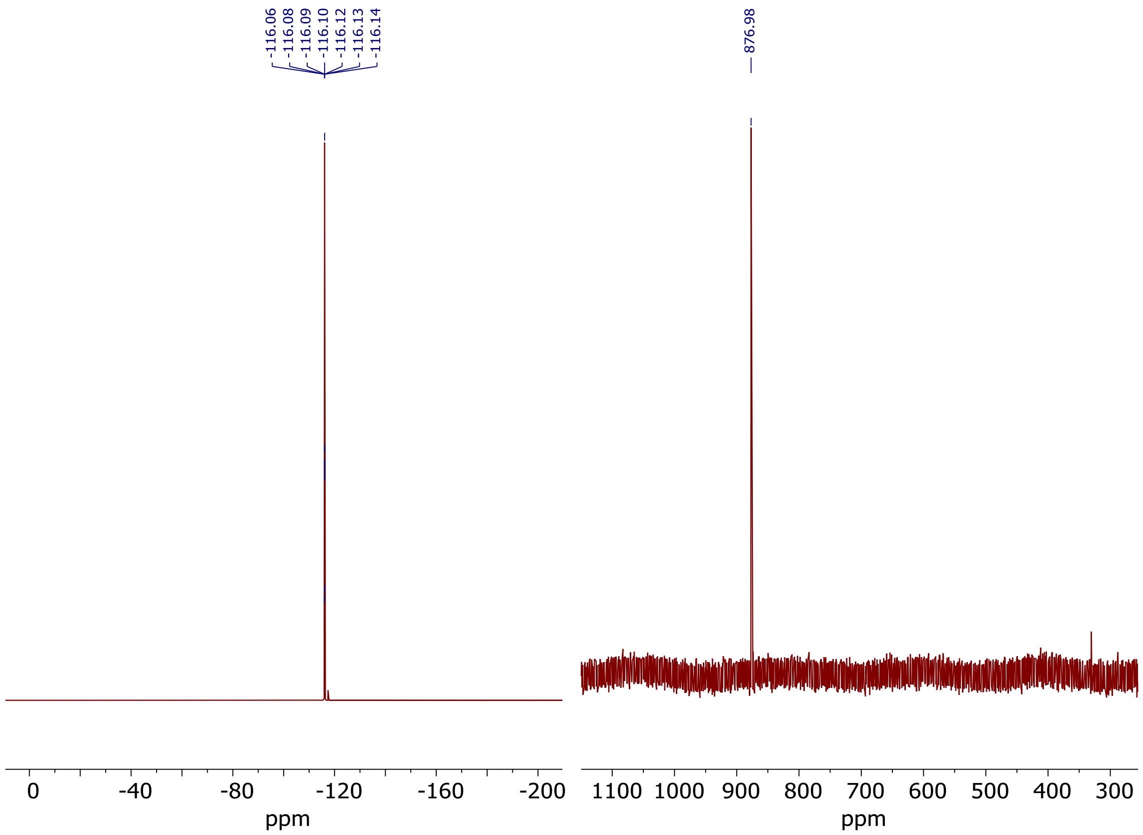

Figure S21. ^{19}F and ^{77}Se NMR spectra of compound **4d**.

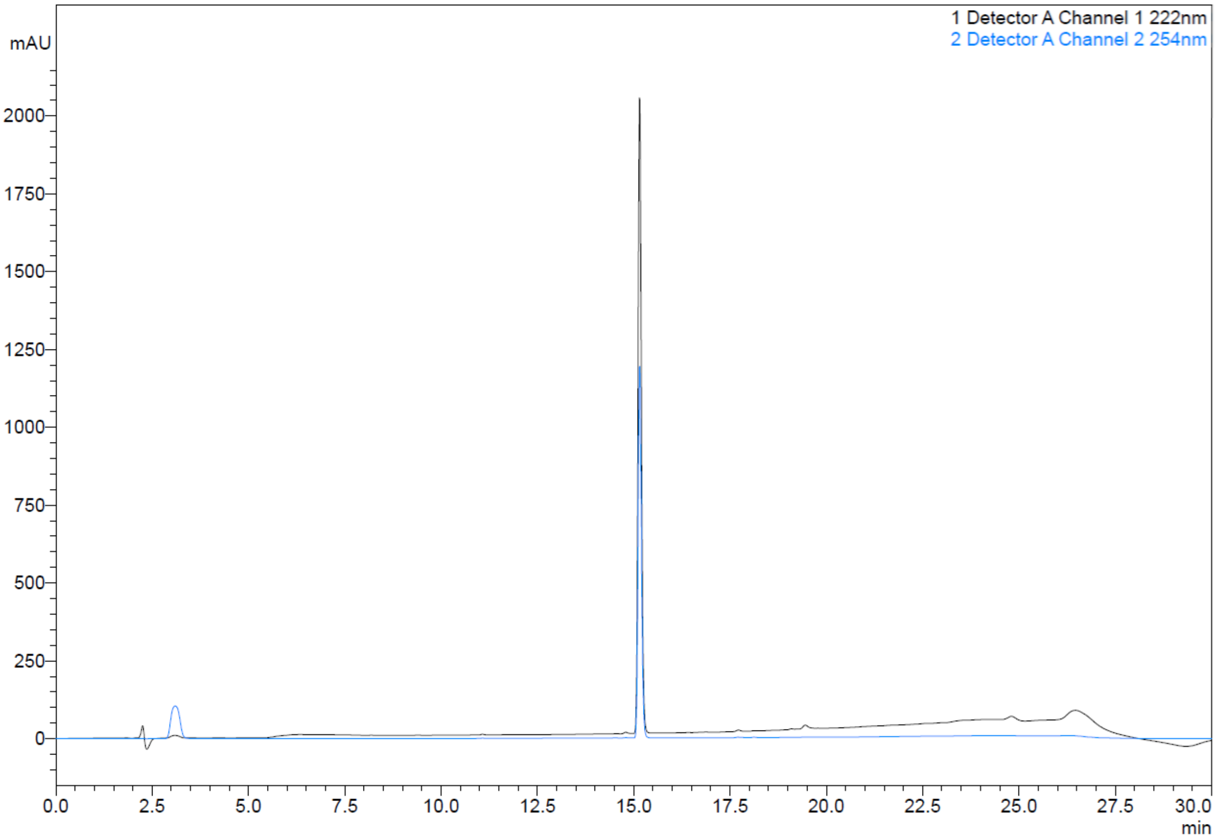

Figure S22. Analytical HPLC analysis of compound **4d**.

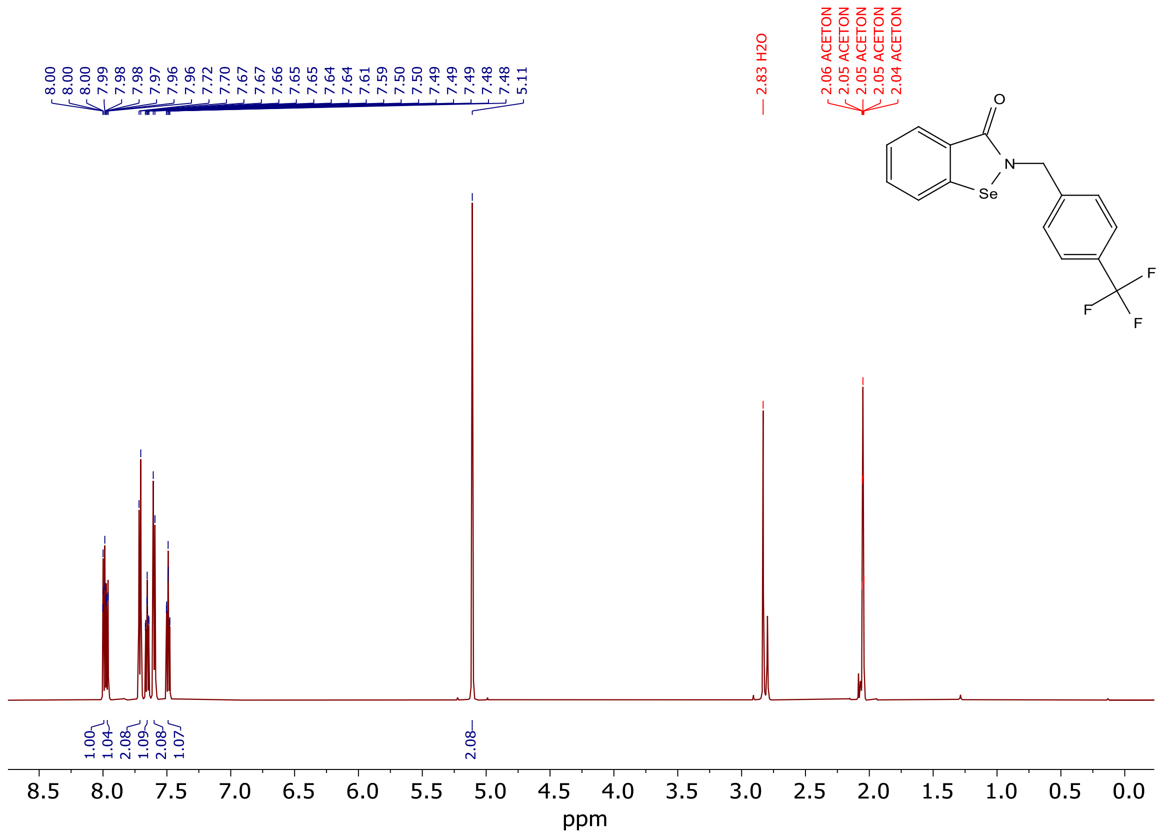

Figure S23. ^{1}H NMR spectrum of compound **4e**.

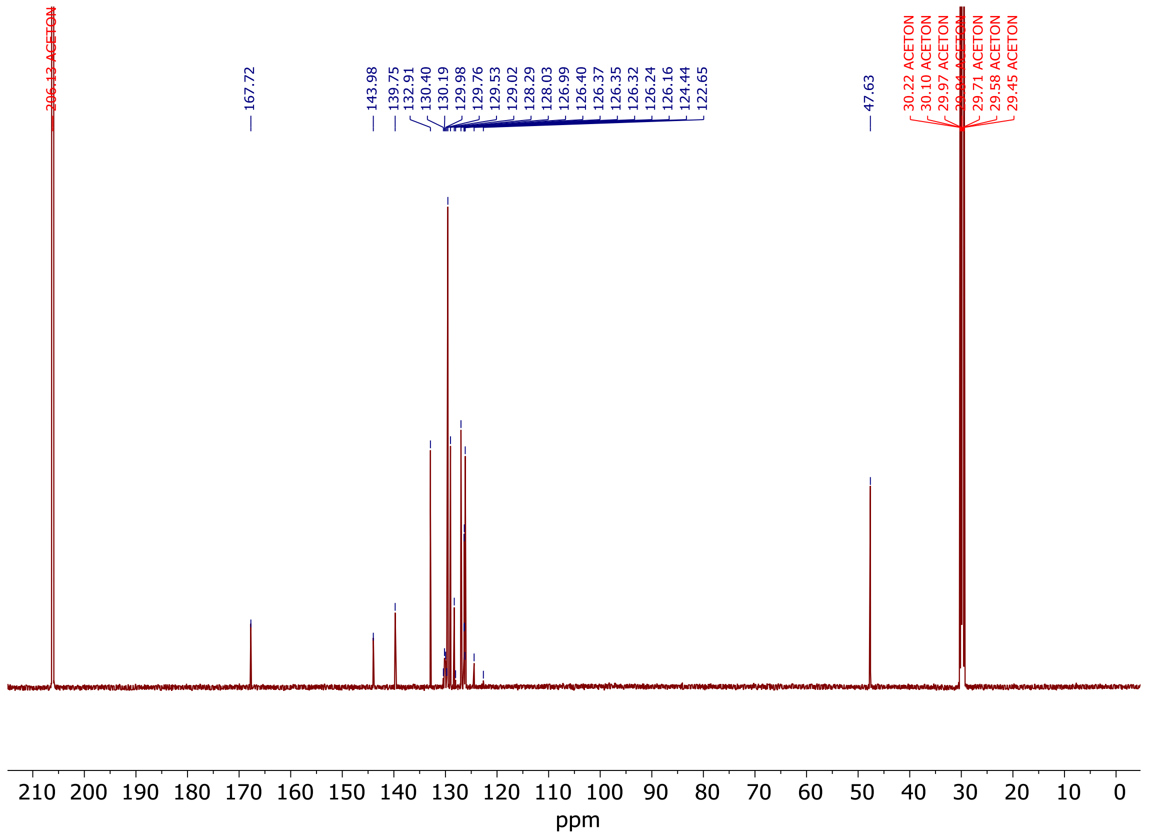

Figure S24. ^{13}C NMR spectrum of compound **4e**.

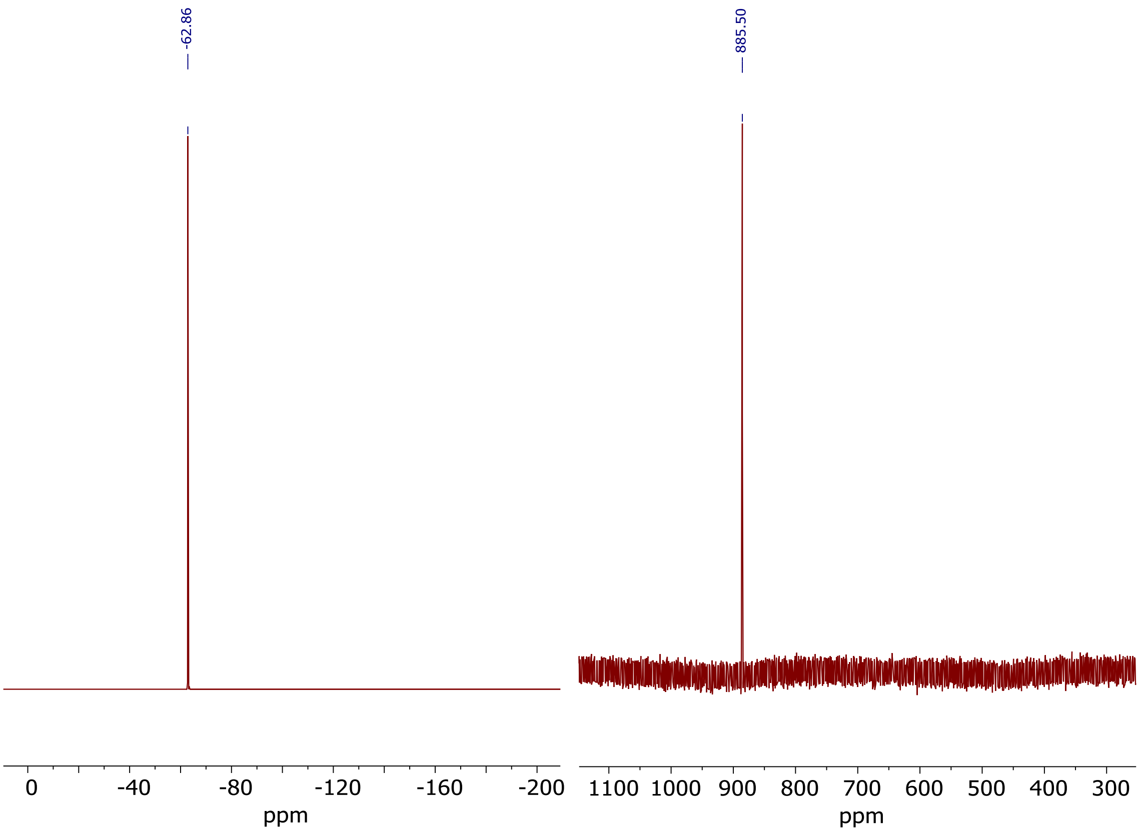

Figure S25. ^{19}F and ^{77}Se NMR spectra of compound **4e**.

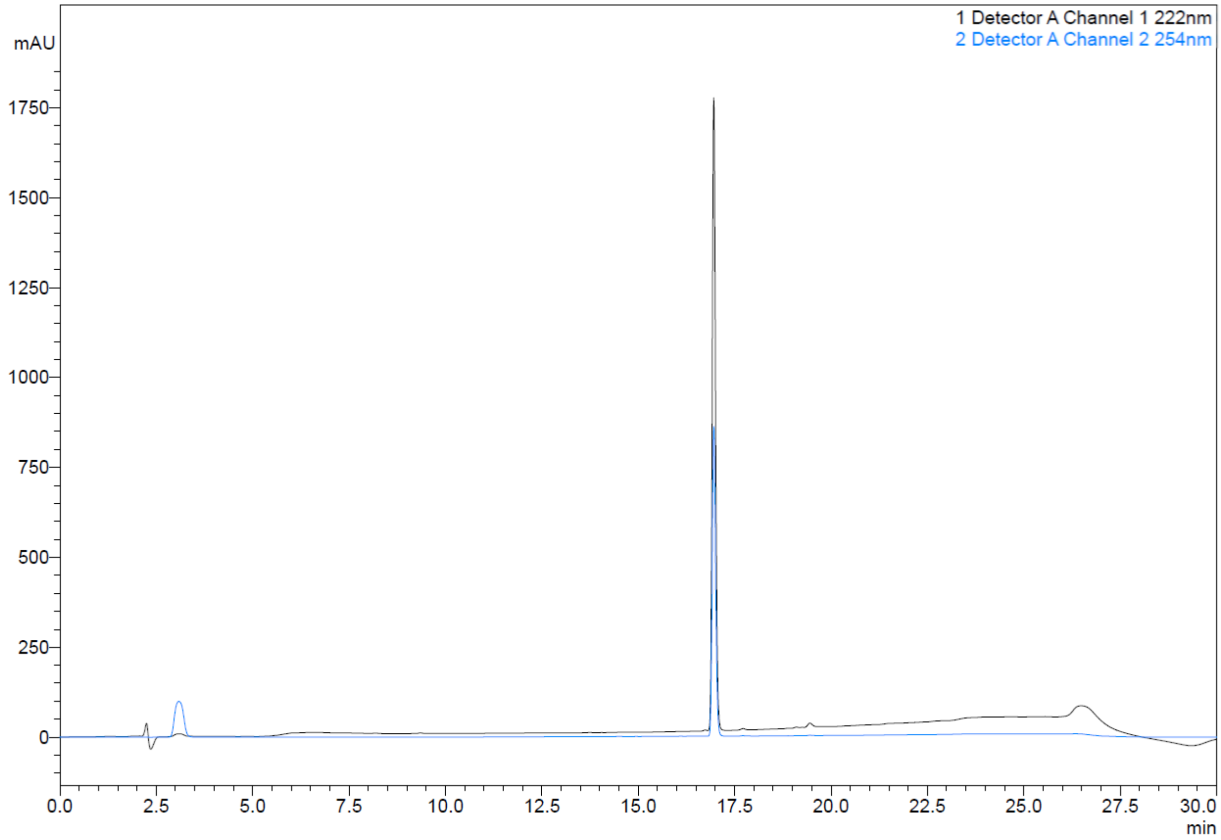

Figure S26. Analytical HPLC analysis of compound **4e**.

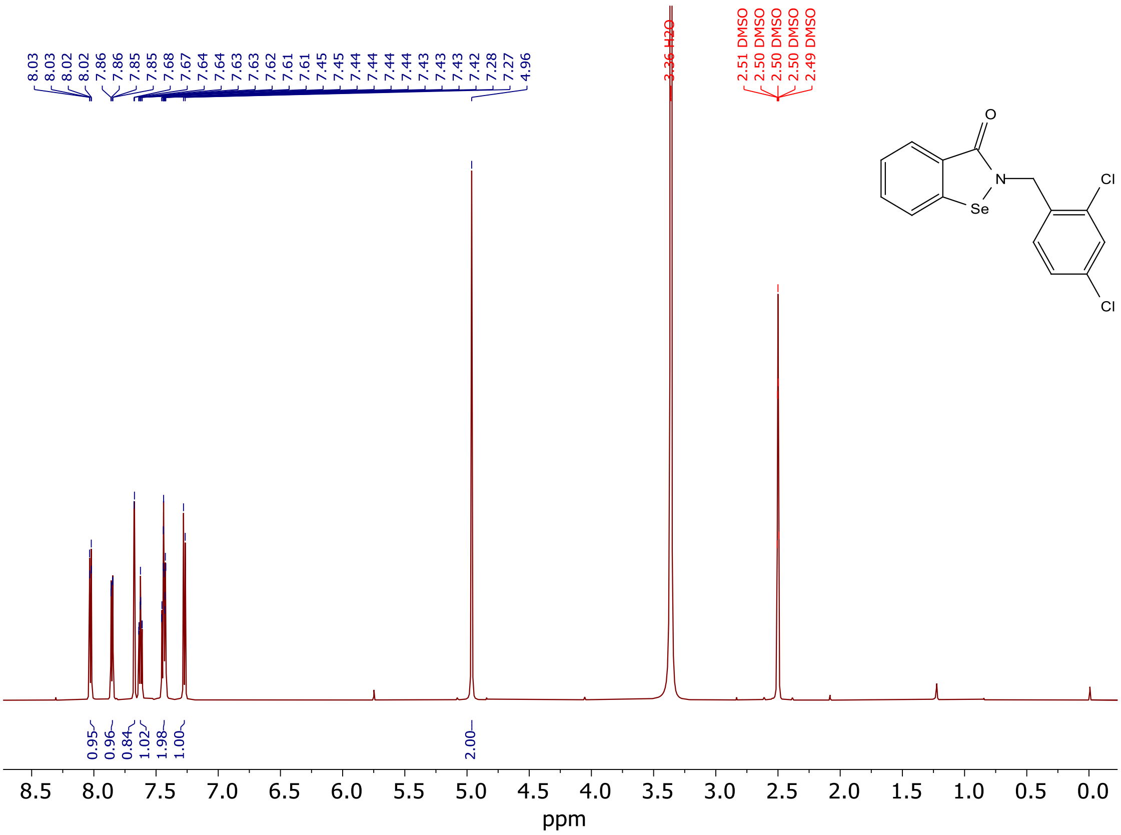

Figure S27. ^{1}H NMR spectrum of compound **4f**.

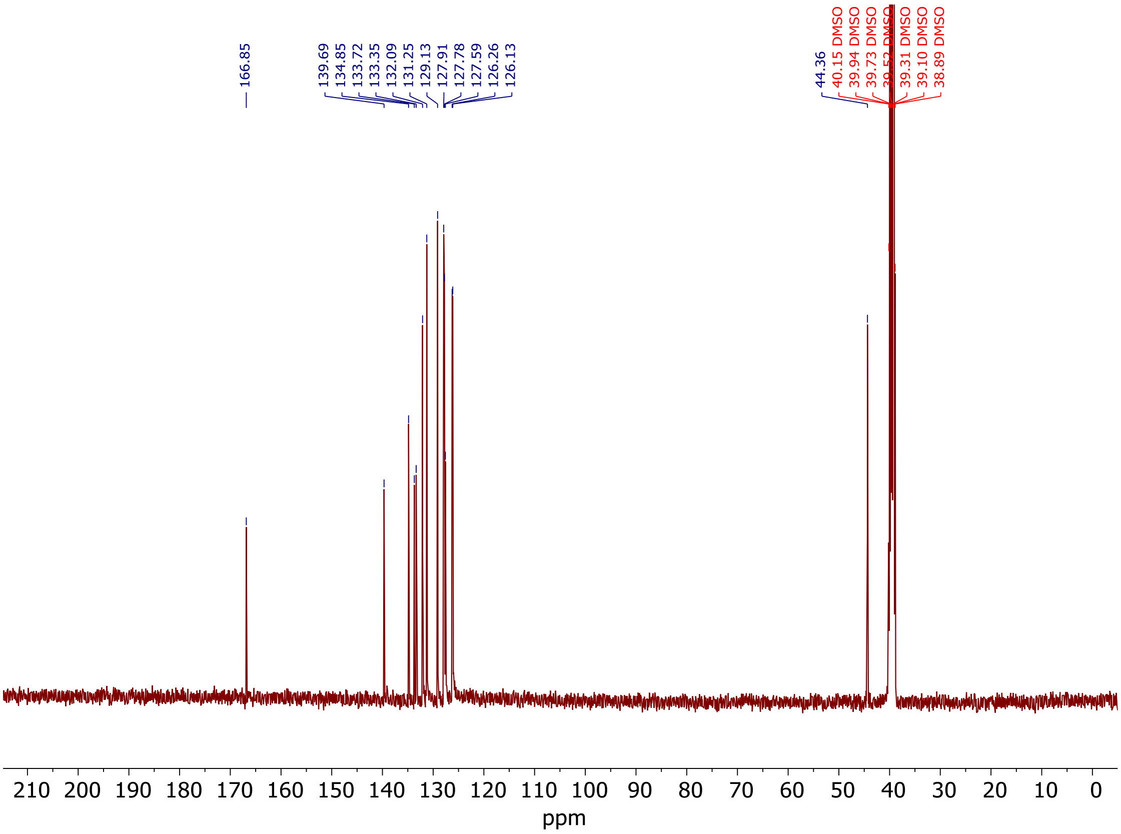

Figure S28. ^{13}C NMR spectrum of compound **4f**.

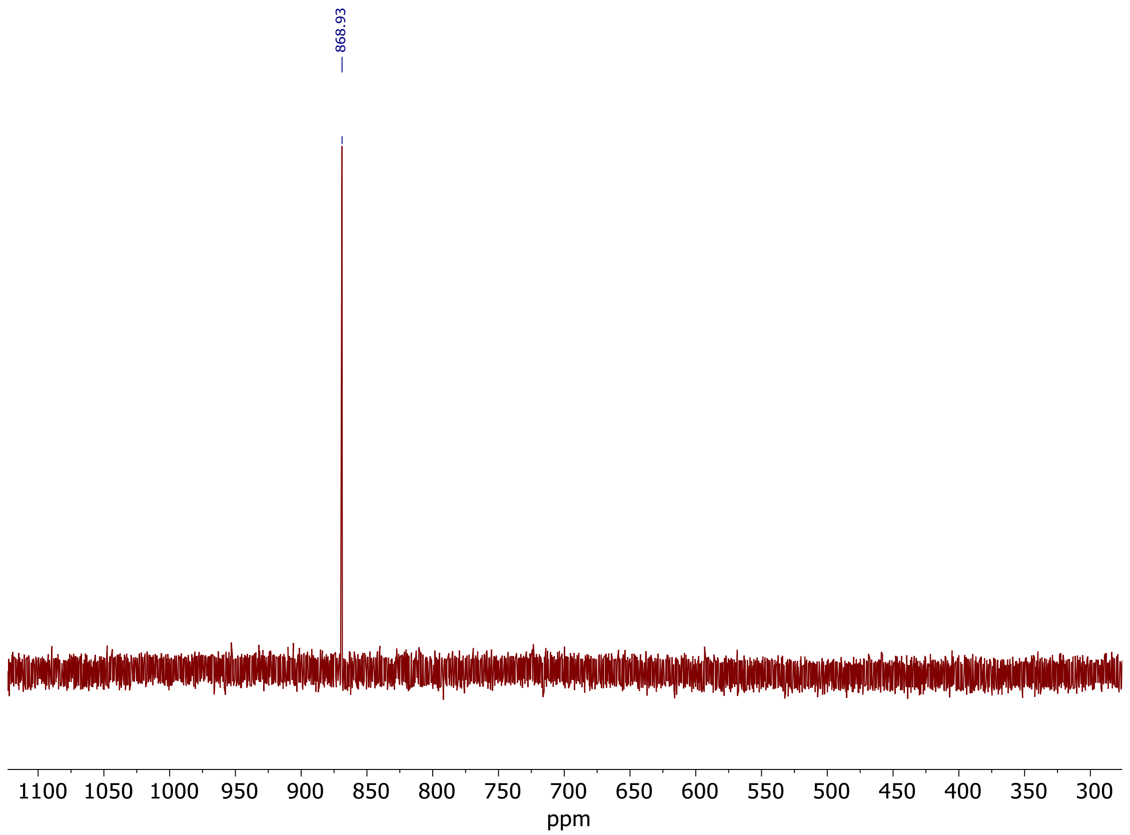

Figure S29. ^{77}Se NMR spectrum of compound **4f**.

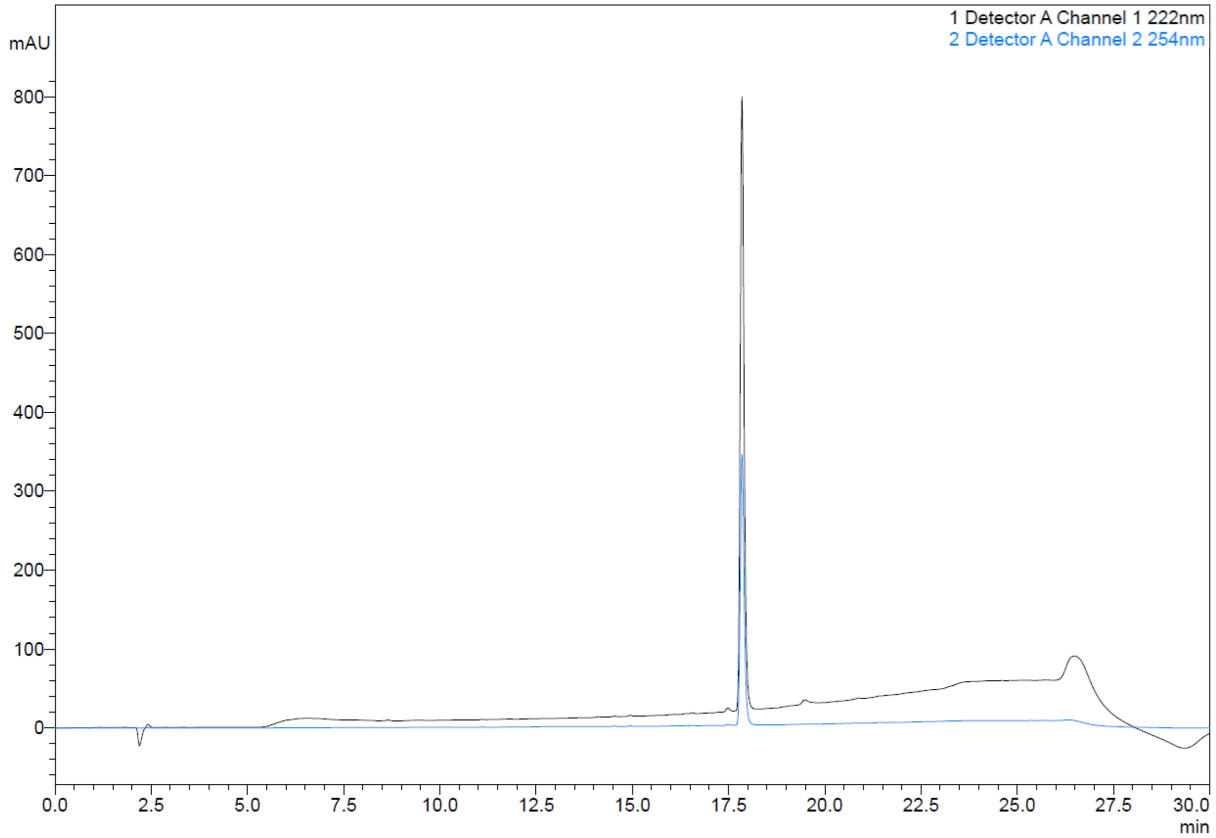

Figure S30. Analytical HPLC analysis of compound **4f**.

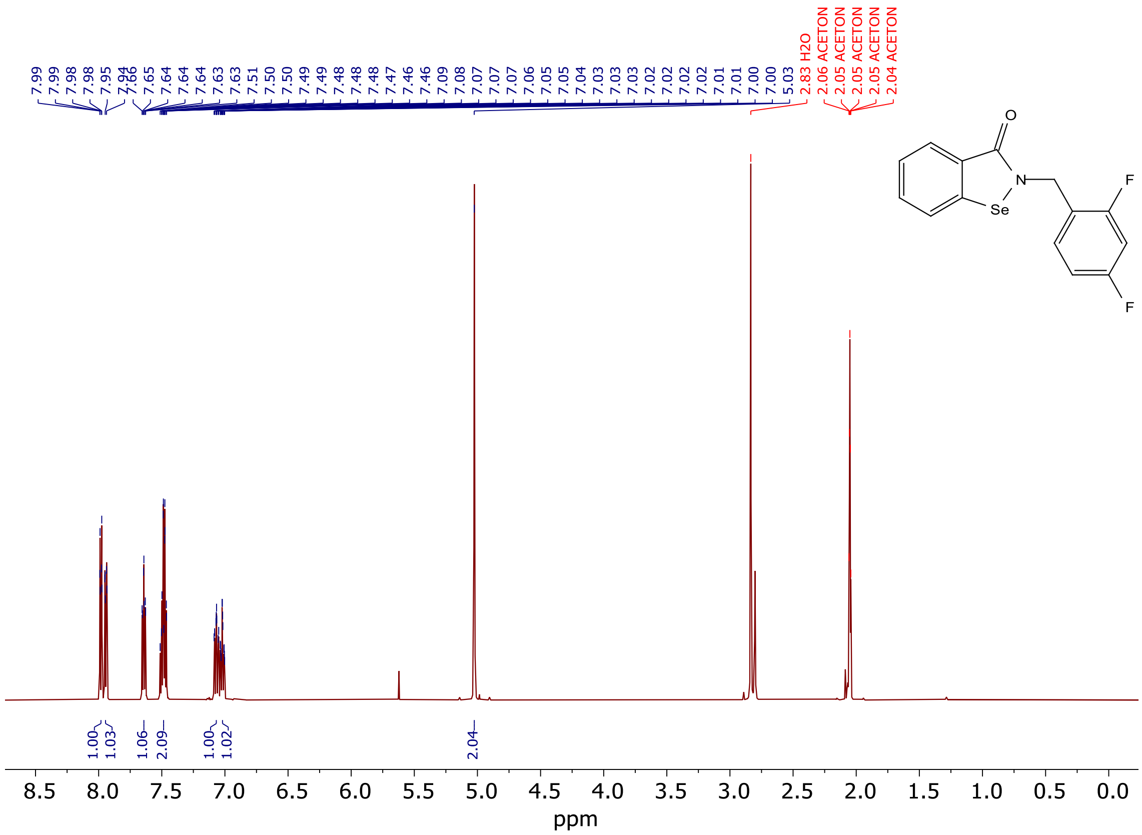

Figure S31. ^{1}H NMR spectrum of compound **4g**.

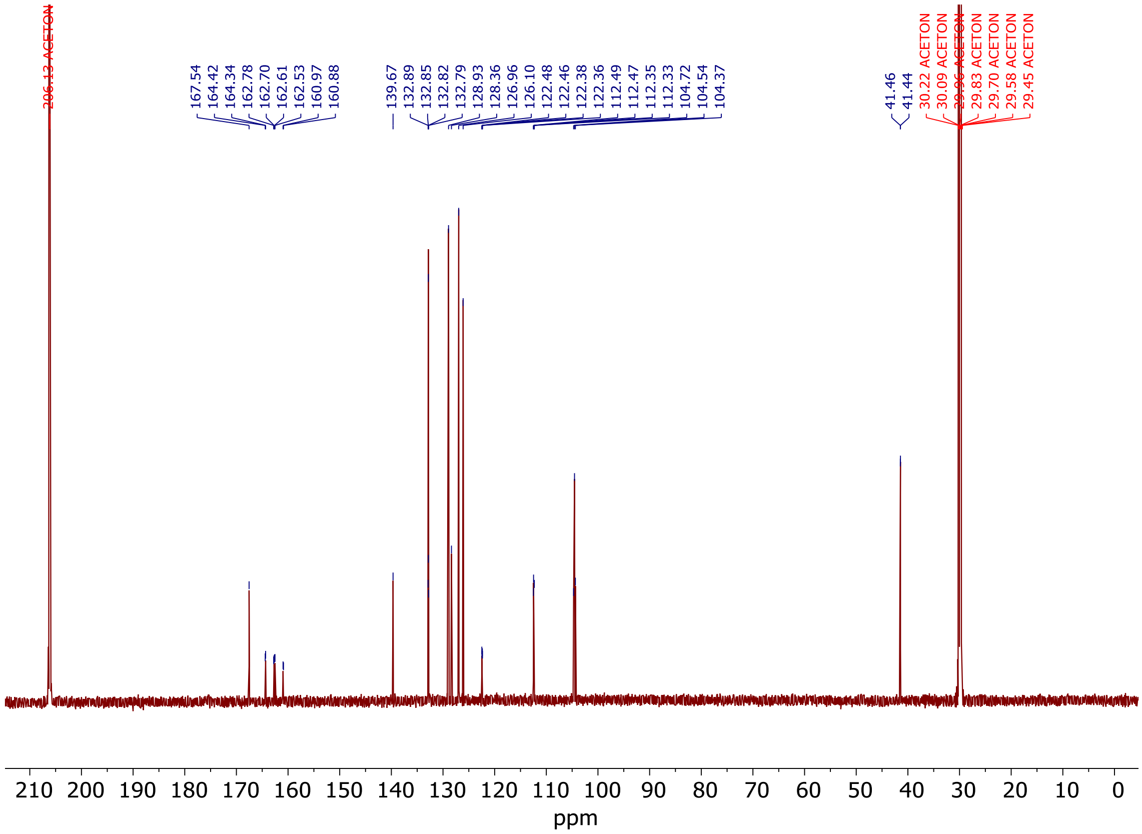

Figure S32. ^{13}C NMR spectrum of compound **4g**.

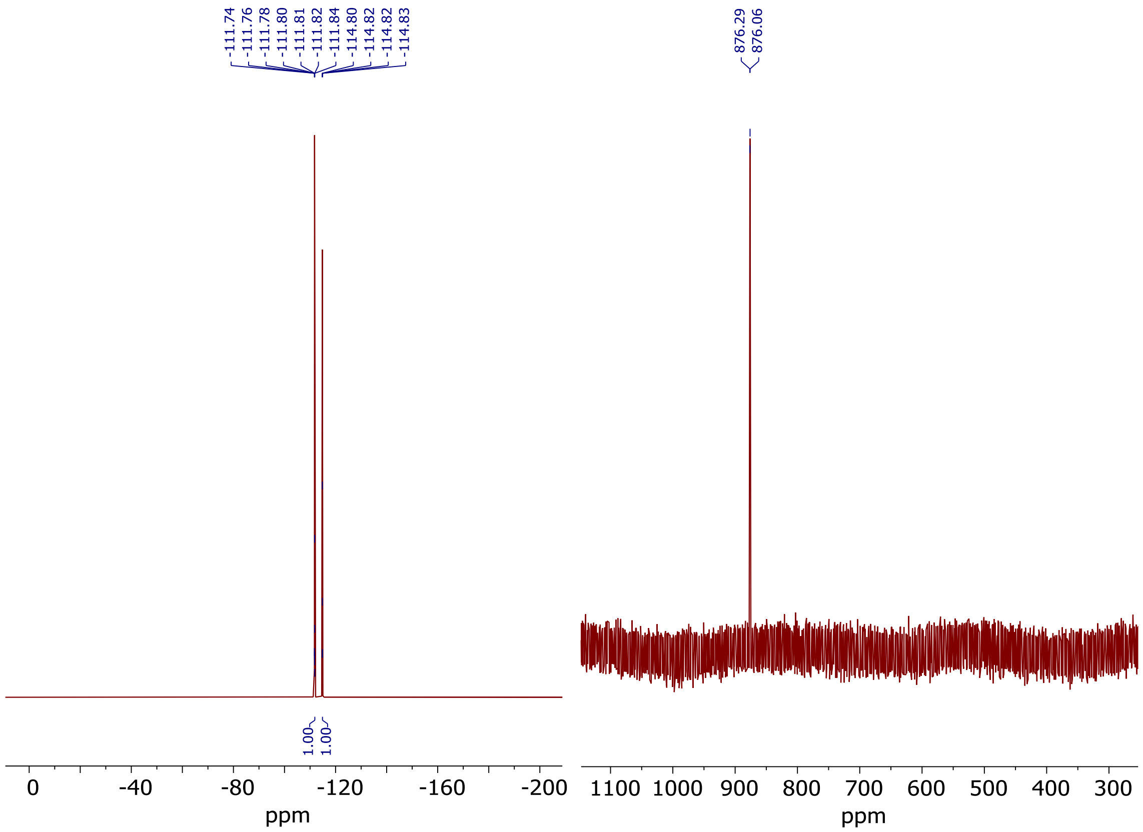

Figure S33. ^{19}F and ^{77}Se NMR spectra of compound **4g**.

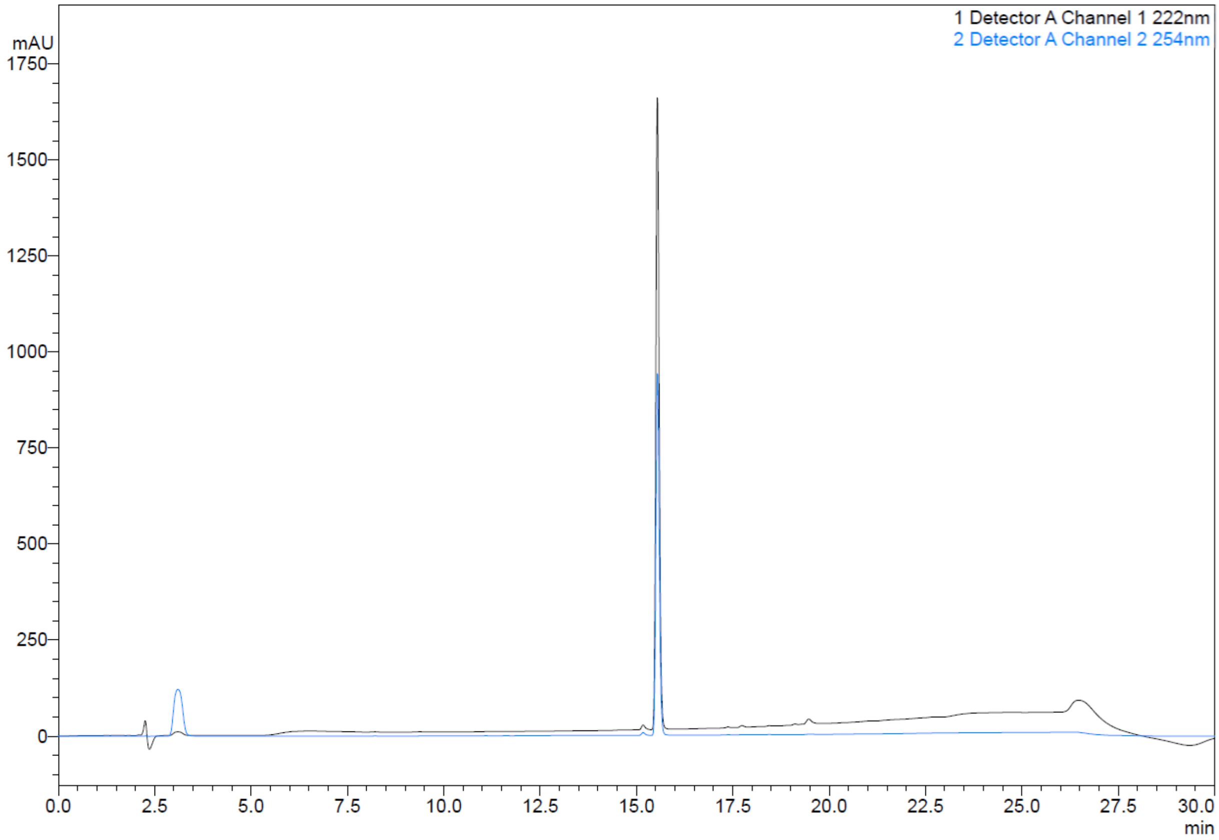

Figure S34. Analytical HPLC analysis of compound **4g**.

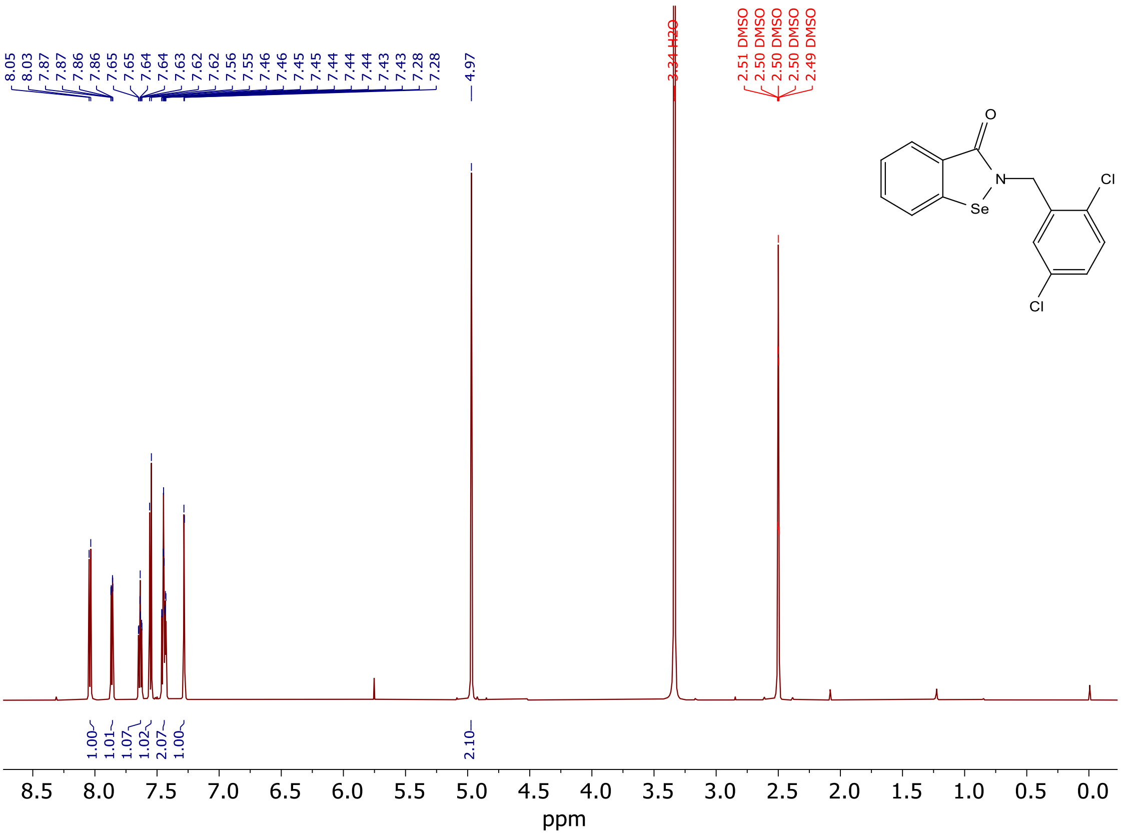

Figure S35. ^{1}H NMR spectrum of compound **4h**.

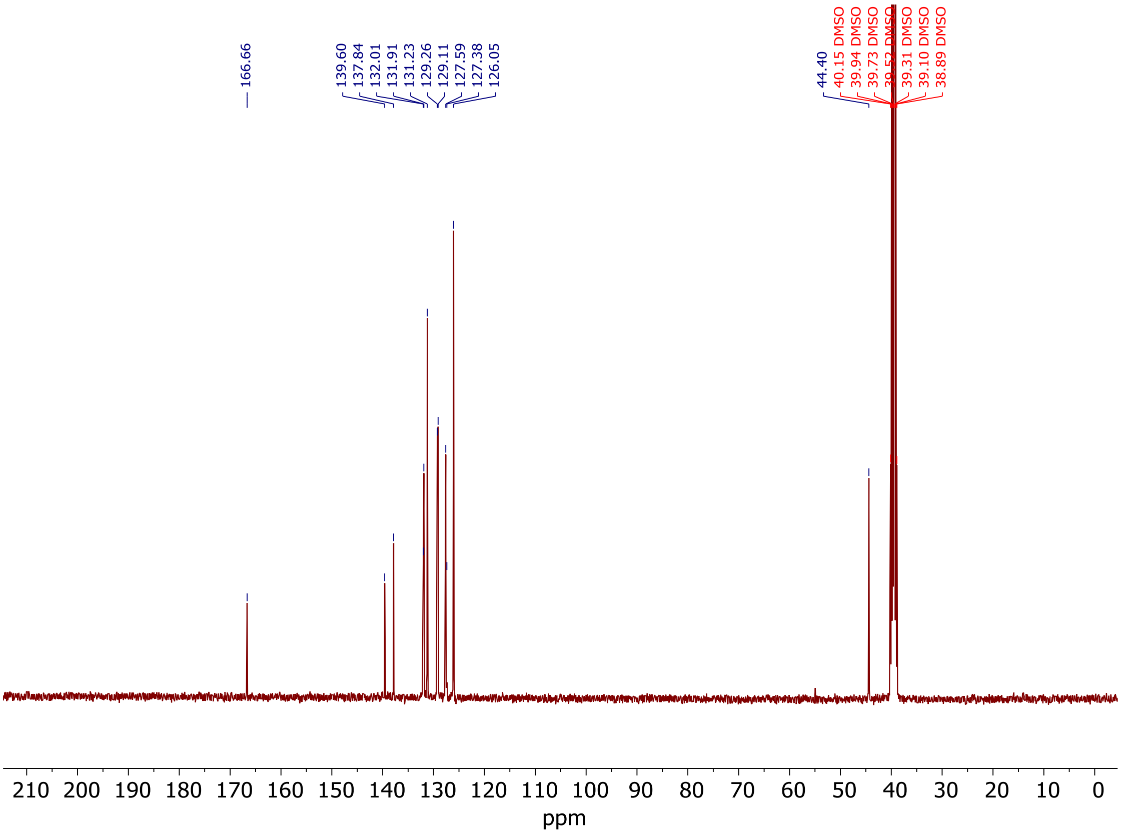

Figure S36. ^{13}C NMR spectrum of compound **4h**.

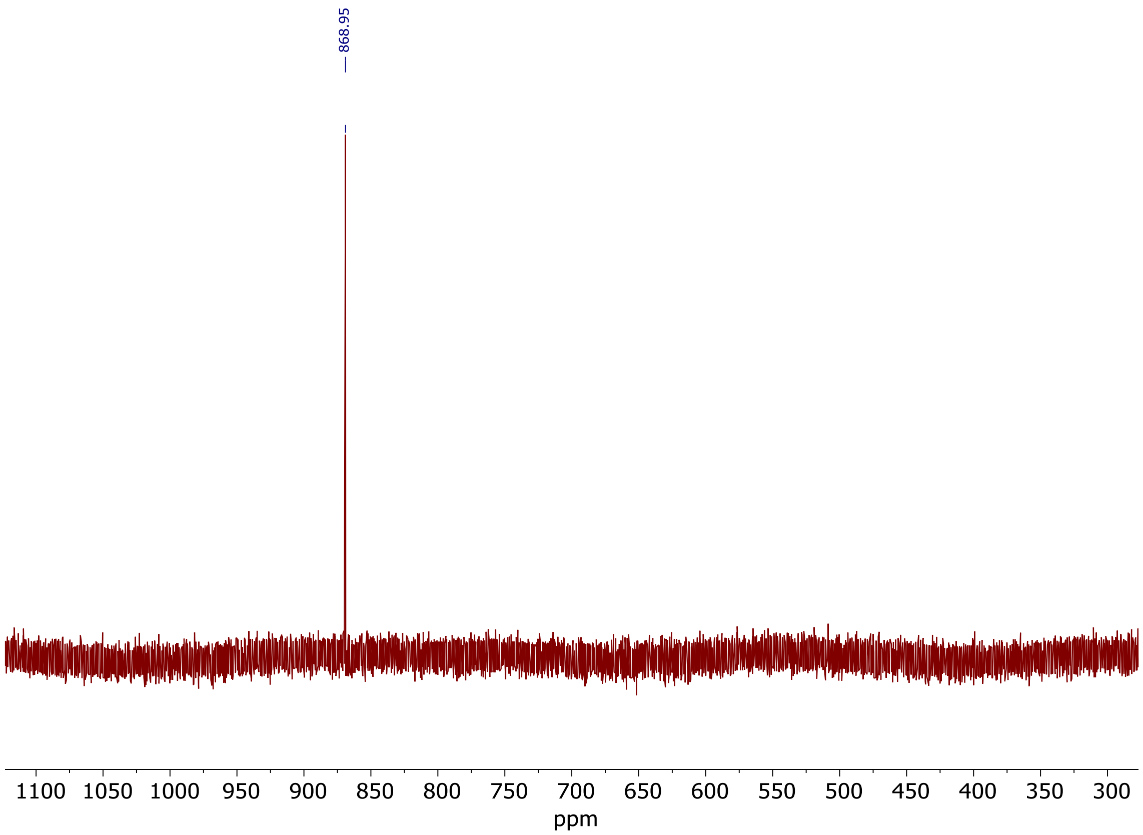

Figure S37. ^{77}Se NMR spectrum of compound **4h**.

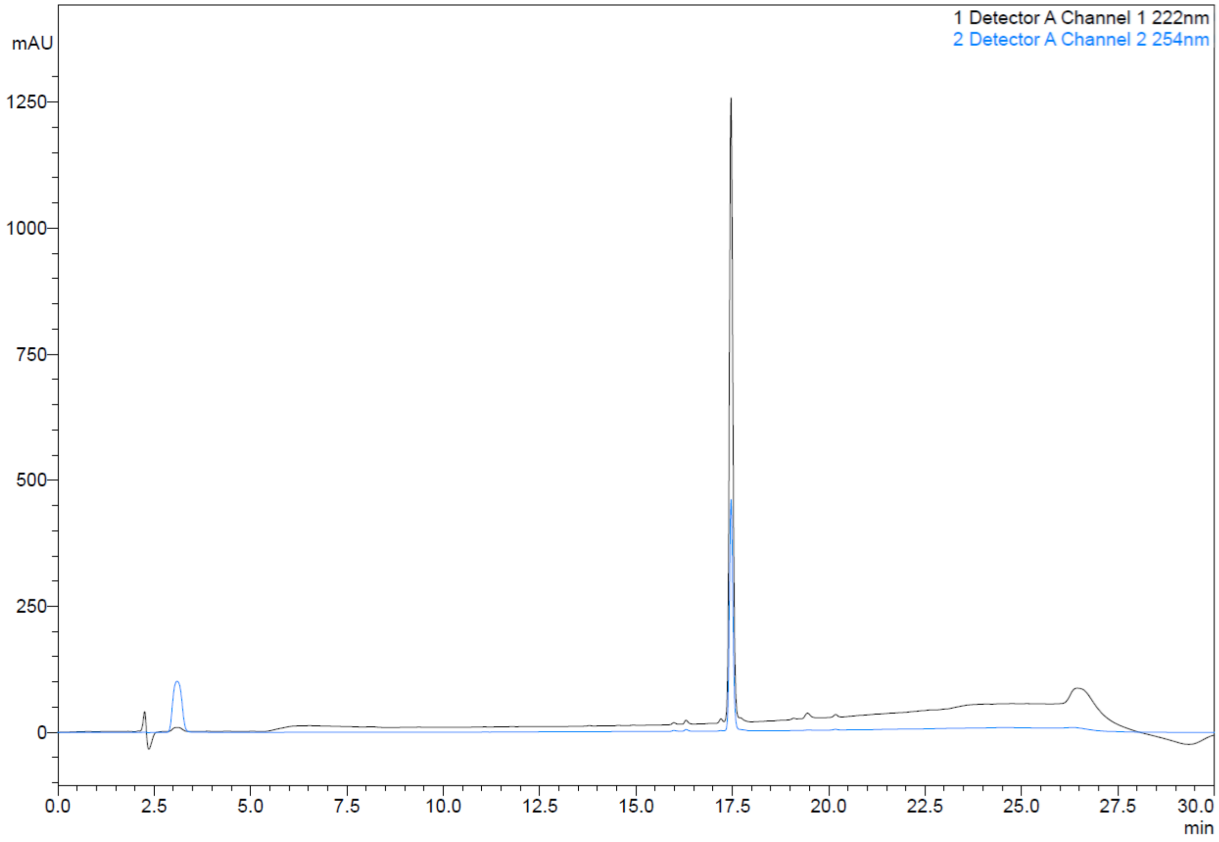

Figure S38. Analytical HPLC analysis of compound **4h**.

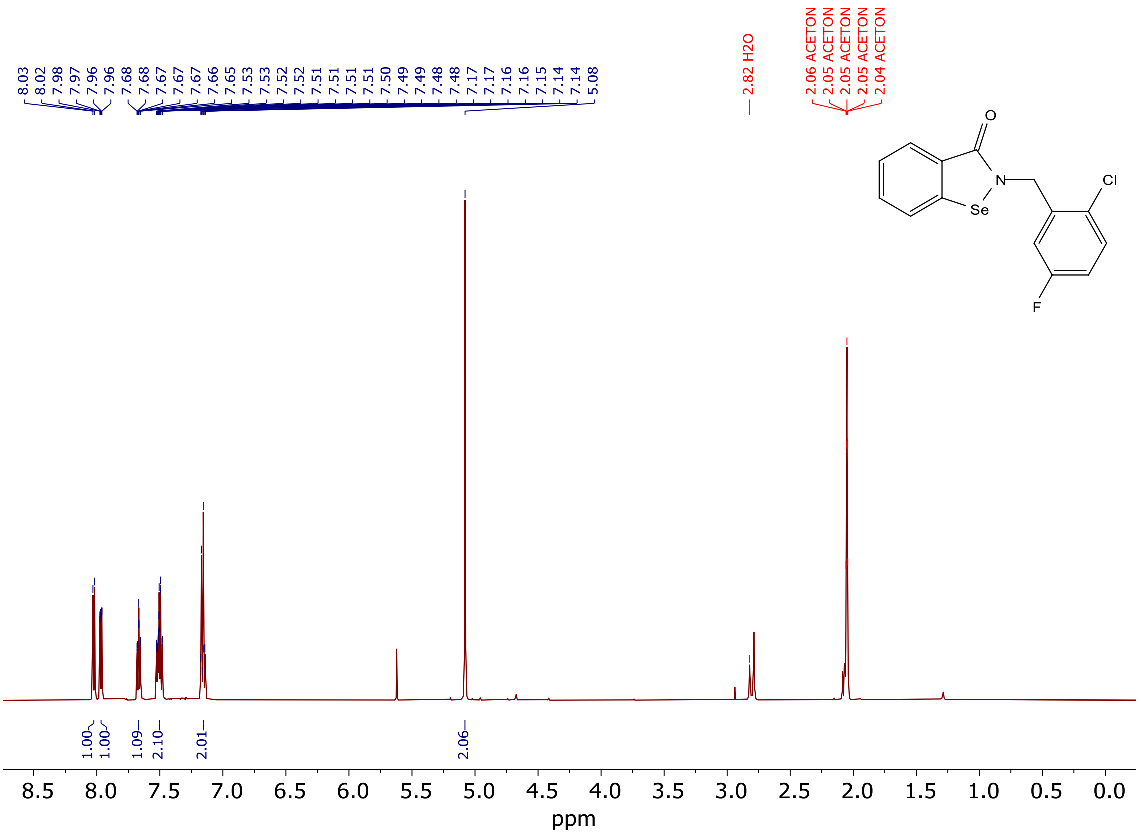

Figure S39. ^{1}H NMR spectrum of compound **4i**.

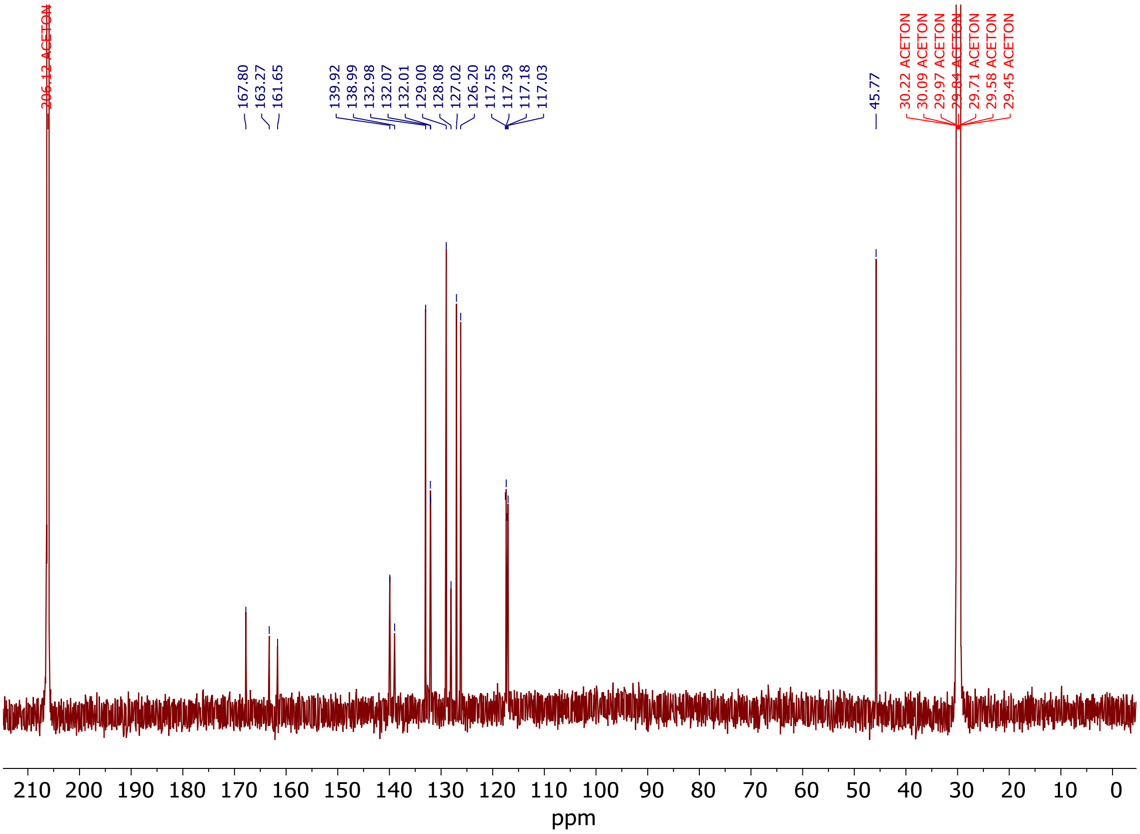

Figure S40. ^{13}C NMR spectrum of compound **4i**.

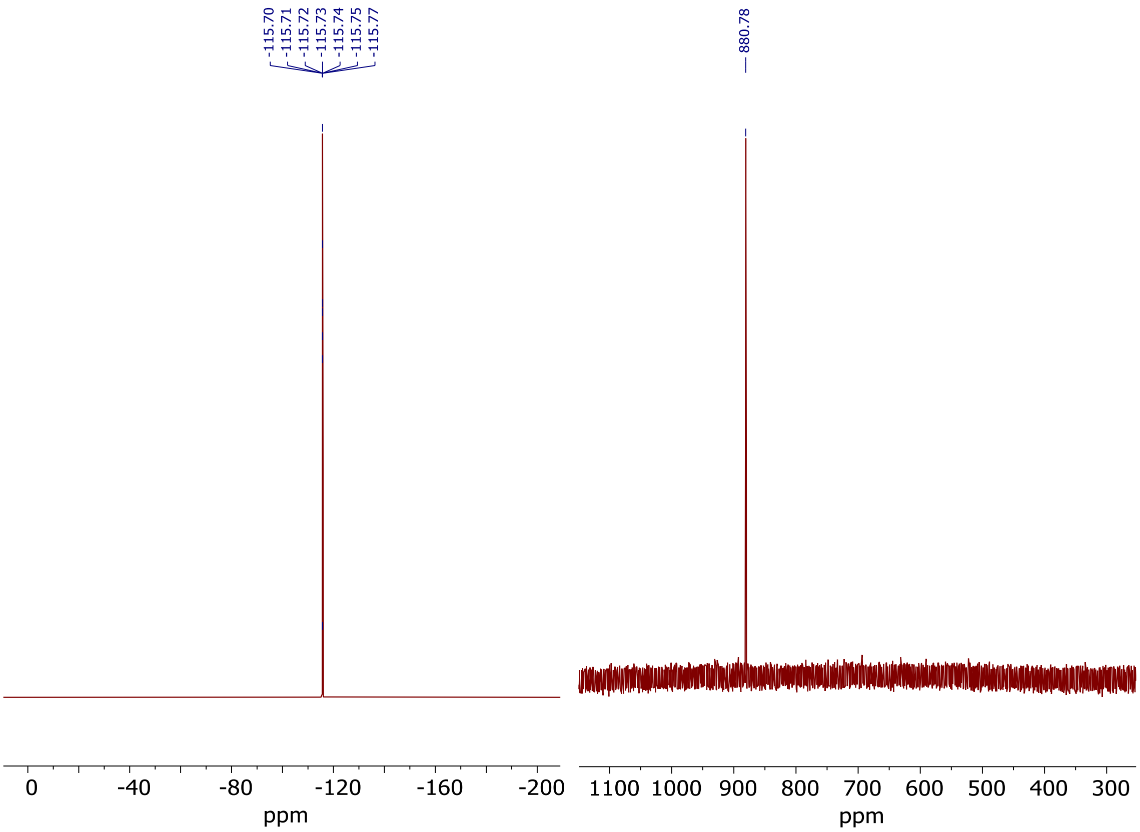

Figure S41. ^{19}F and ^{77}Se NMR spectra of compound **4i**.

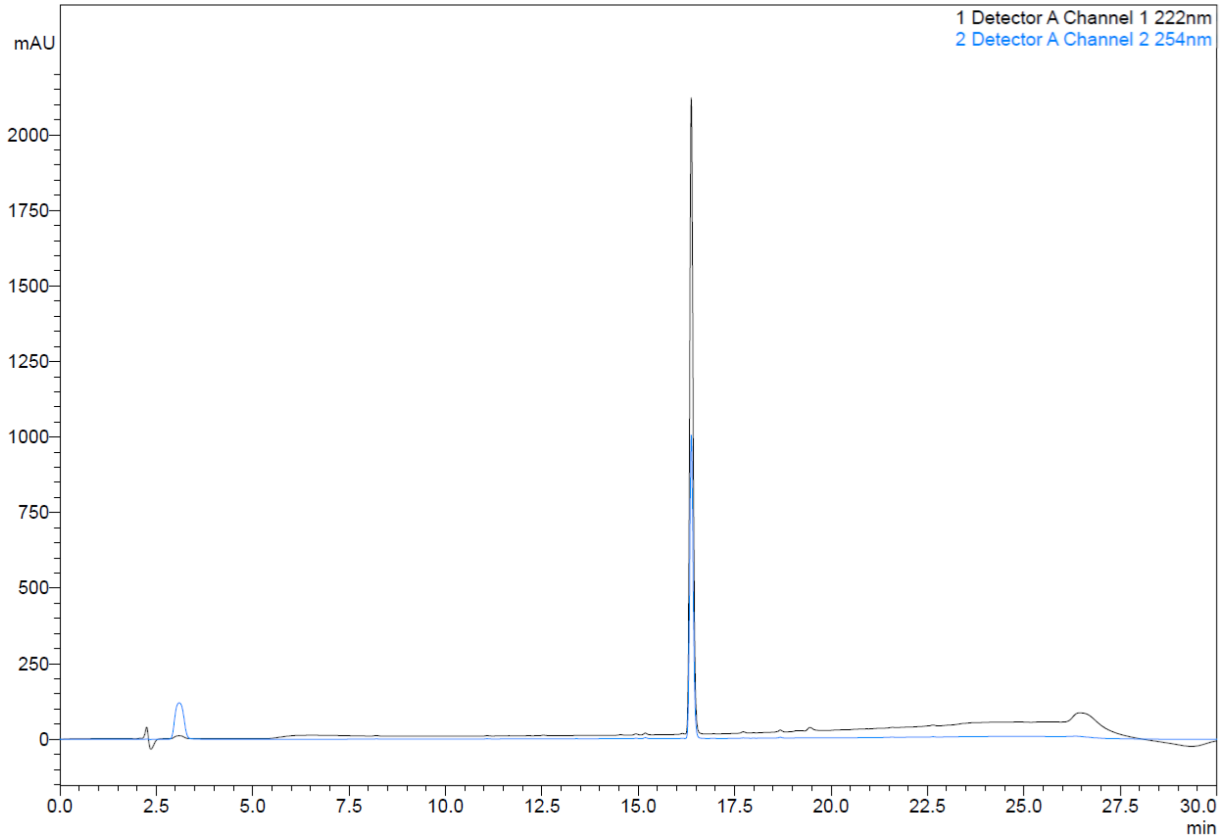

Figure S42. Analytical HPLC analysis of compound **4i**.

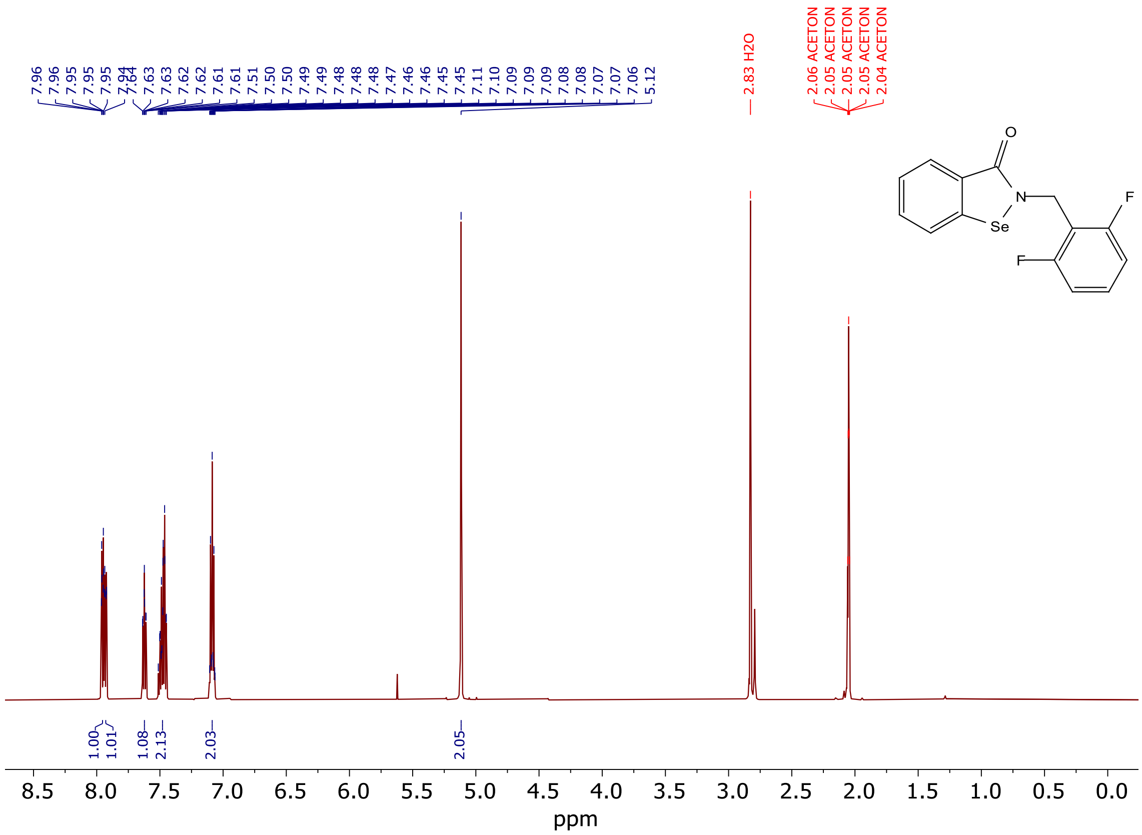

Figure S43. ^{1}H NMR spectrum of compound **4j**.

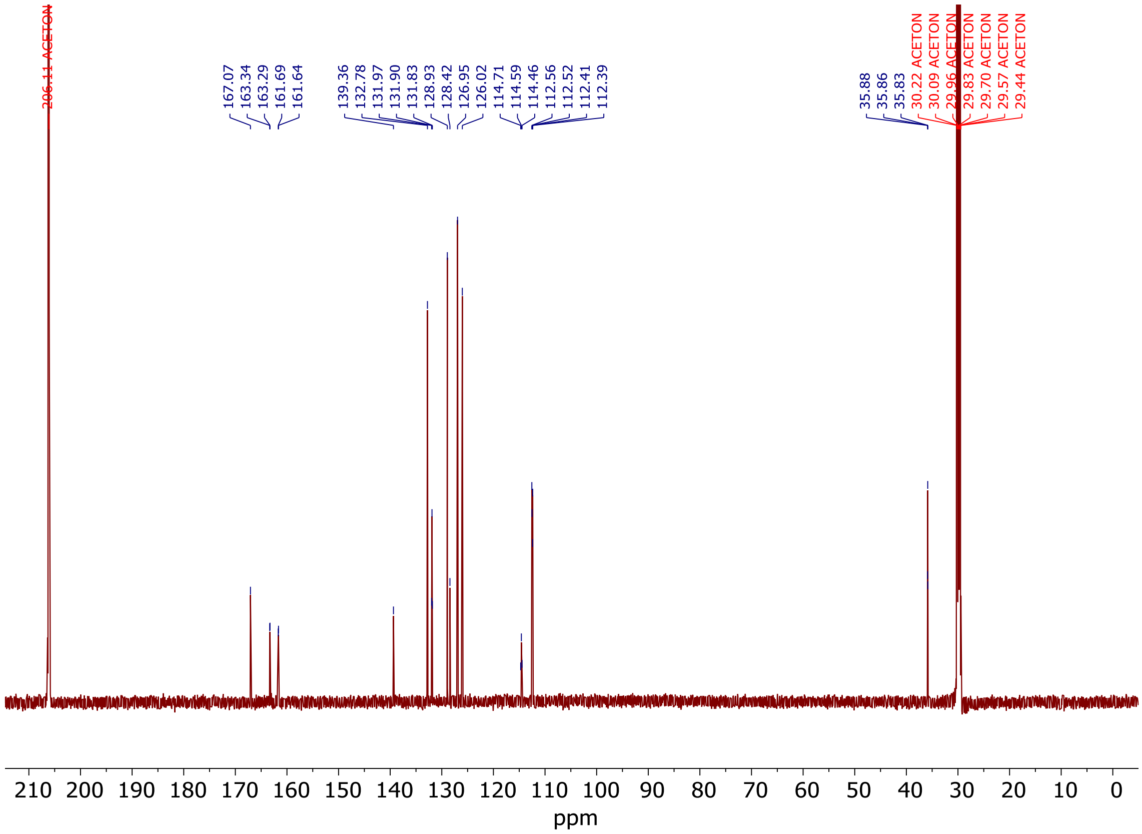

Figure S44. ^{13}C NMR spectrum of compound **4j**.

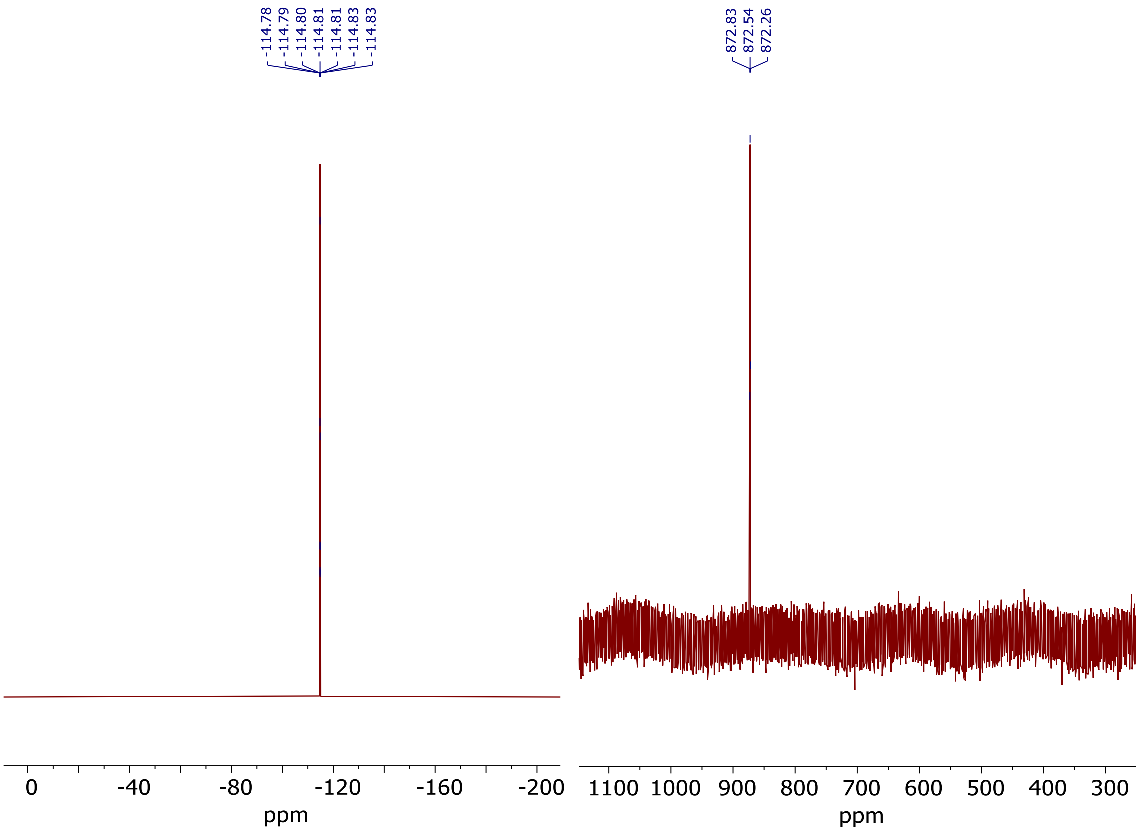

Figure S45. ^{19}F and ^{77}Se NMR spectra of compound **4j**.

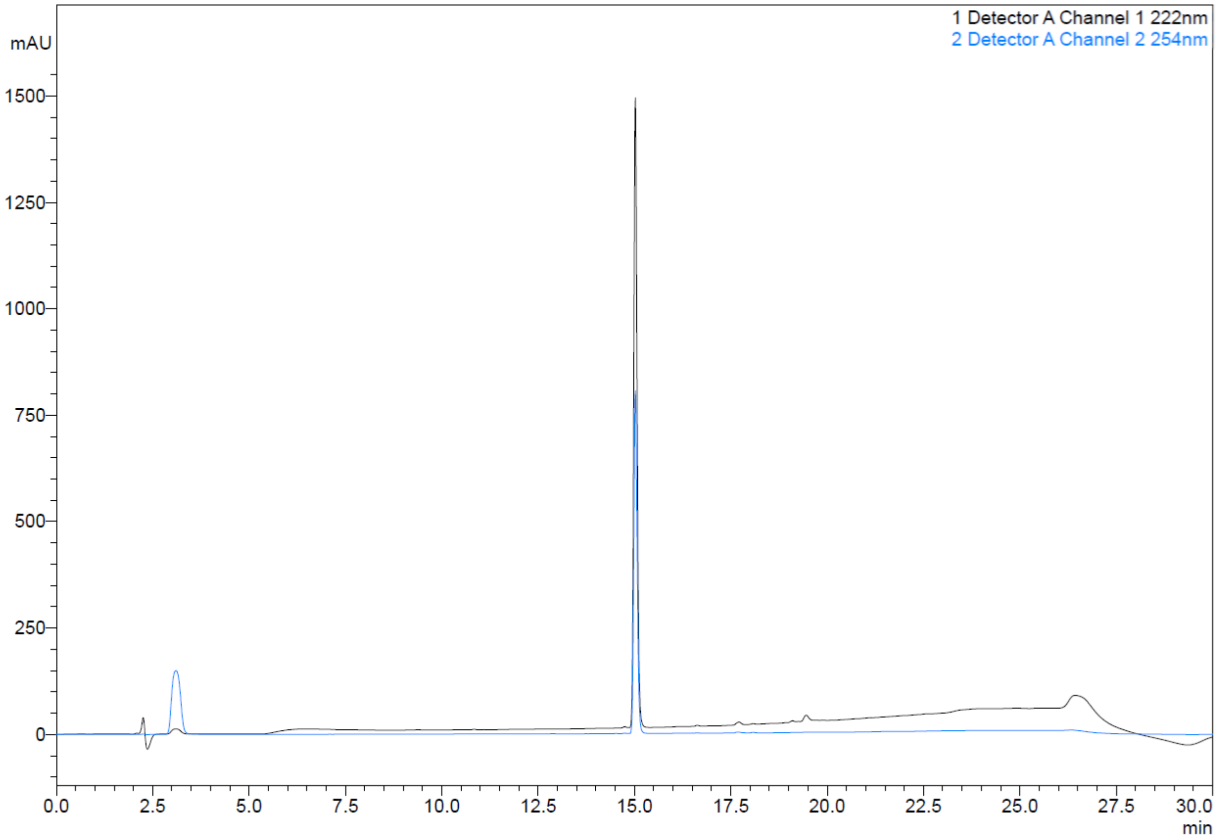

Figure S46. Analytical HPLC analysis of compound **4j**.

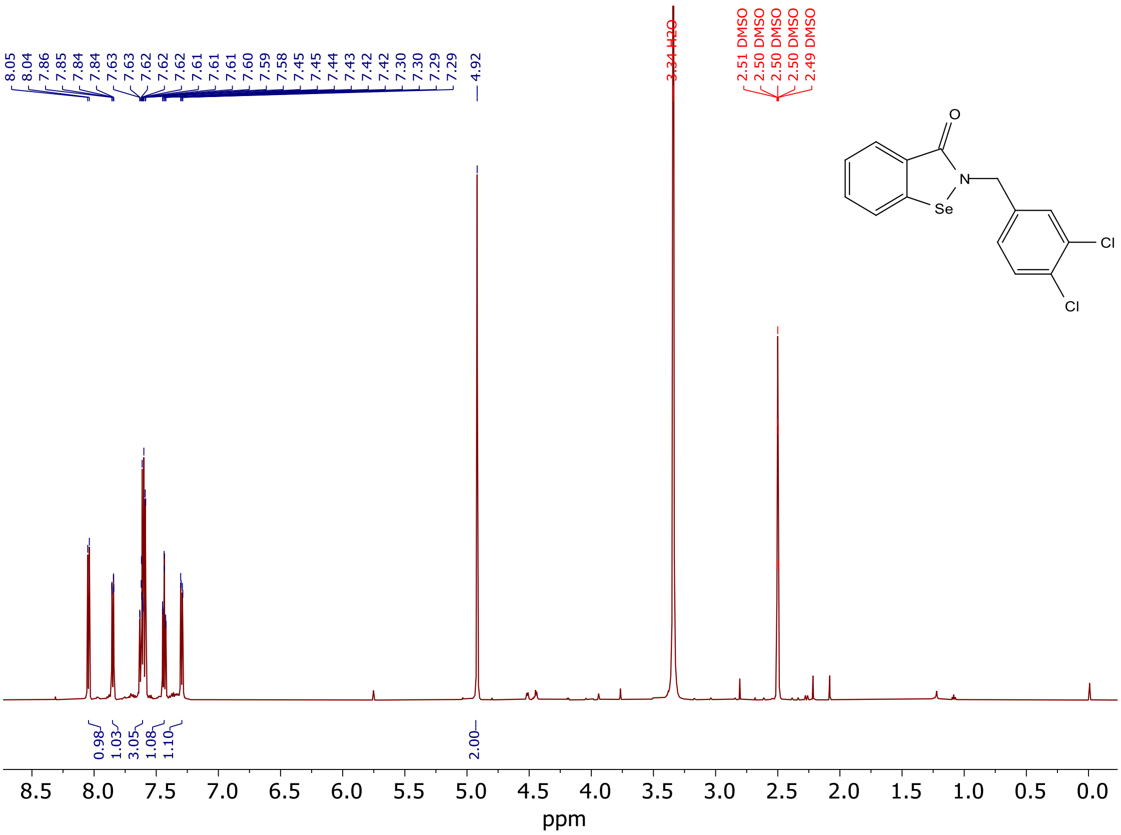

Figure S47. ^{1}H NMR spectrum of compound **4k**.

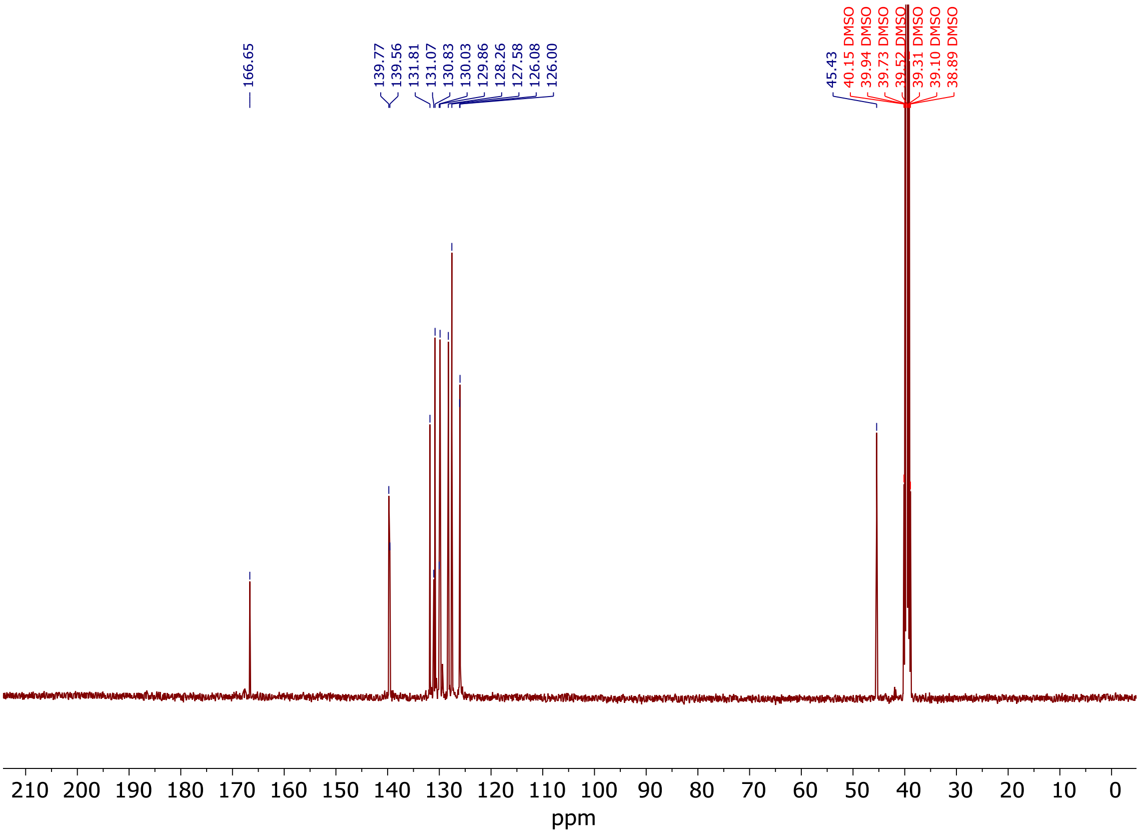

Figure S48. ^{13}C NMR spectrum of compound **4k**.

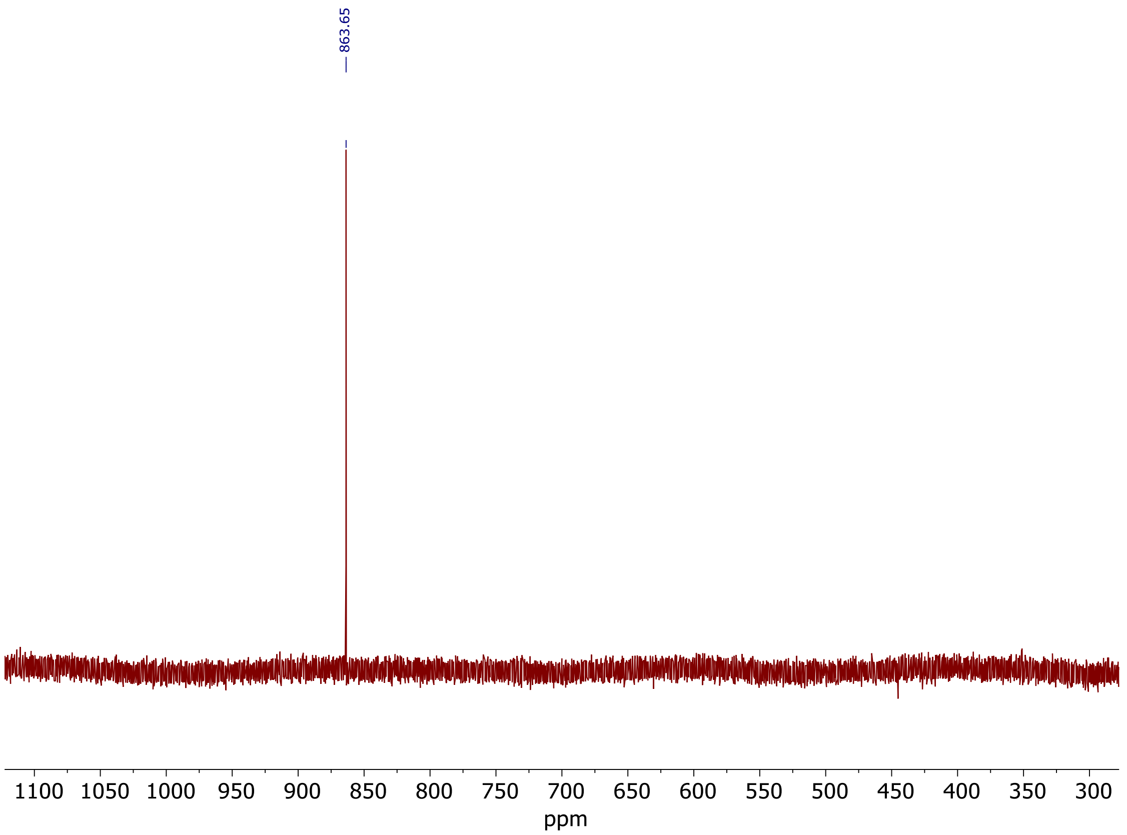

Figure S49. ^{77}Se NMR spectrum of compound **4k**.

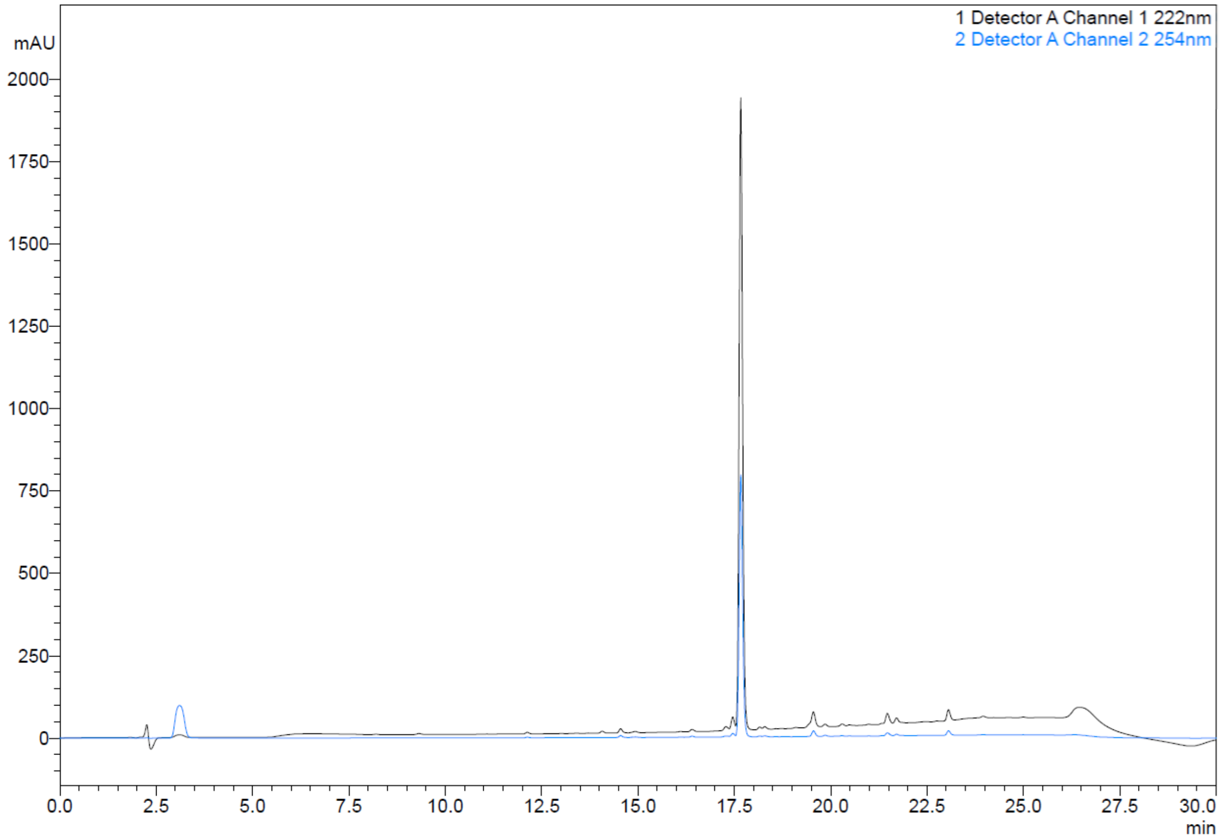

Figure S50. Analytical HPLC analysis of compound **4k**.

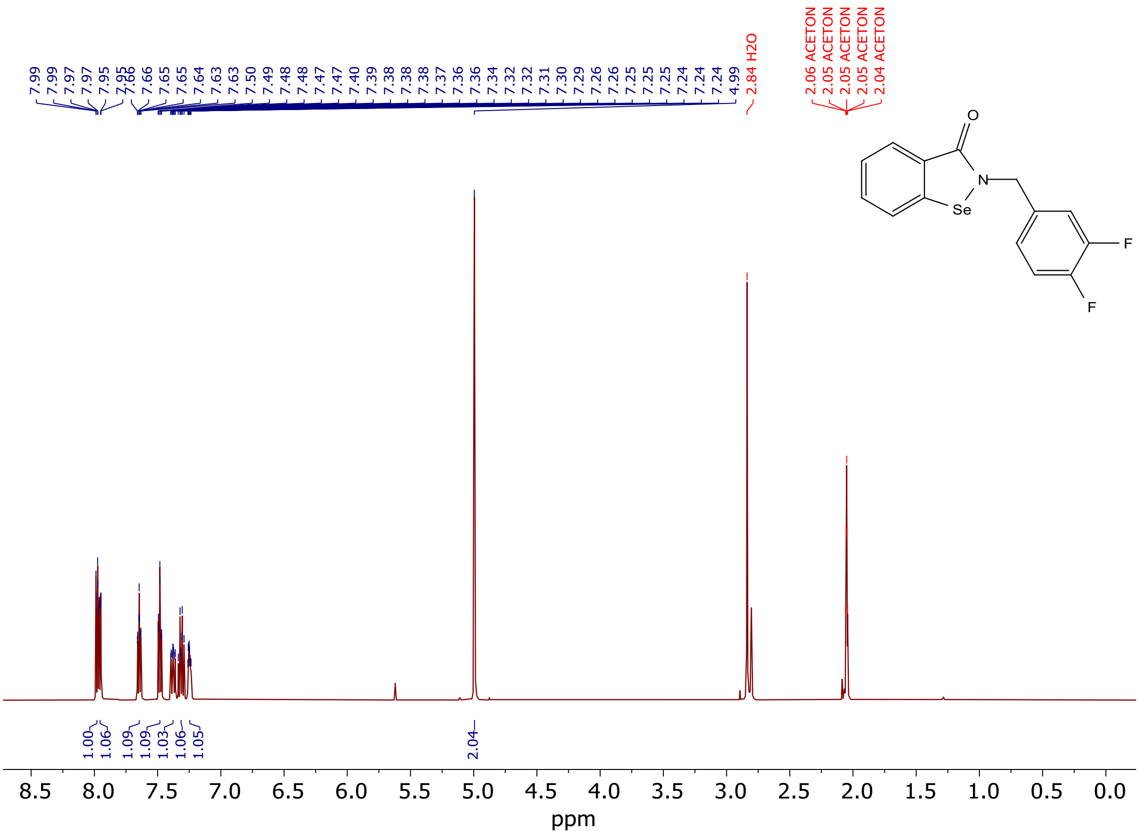

Figure S51. ^{1}H NMR spectrum of compound **4l**.

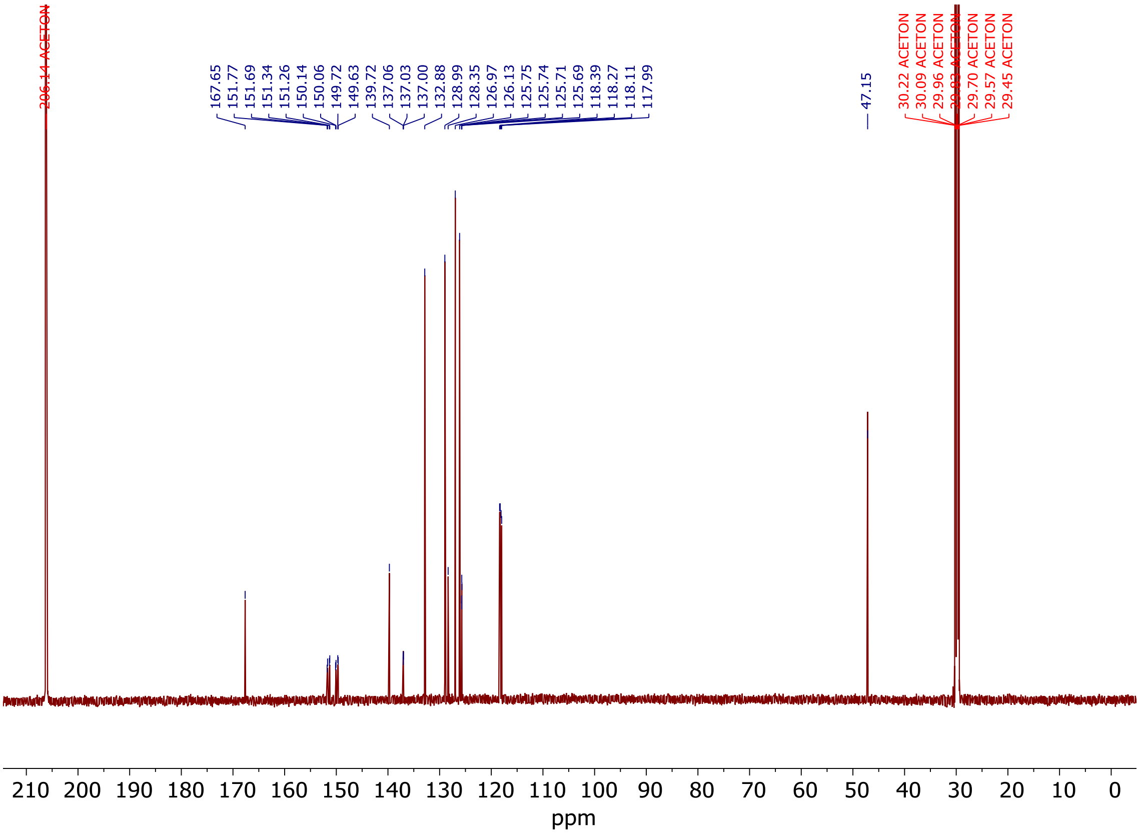

Figure S52. ^{13}C NMR spectrum of compound **4l**.

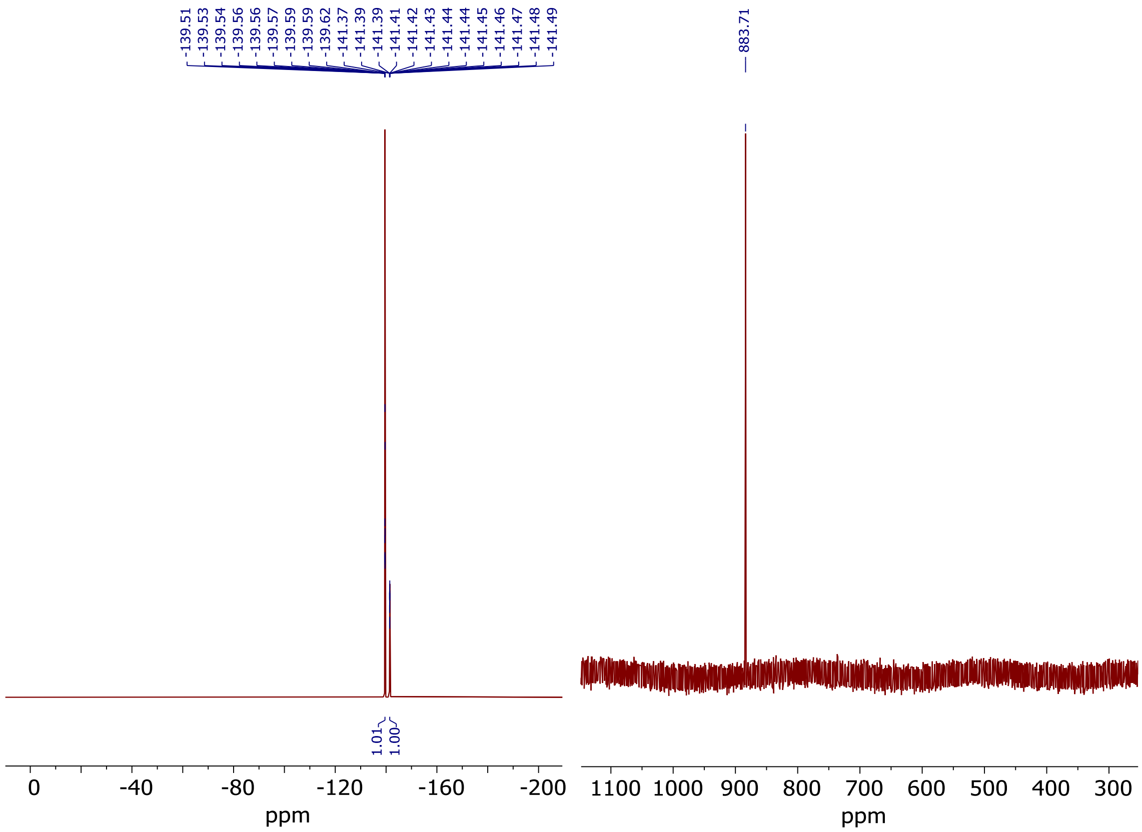

Figure S53. ^{19}F and ^{77}Se NMR spectra of compound **4l**.

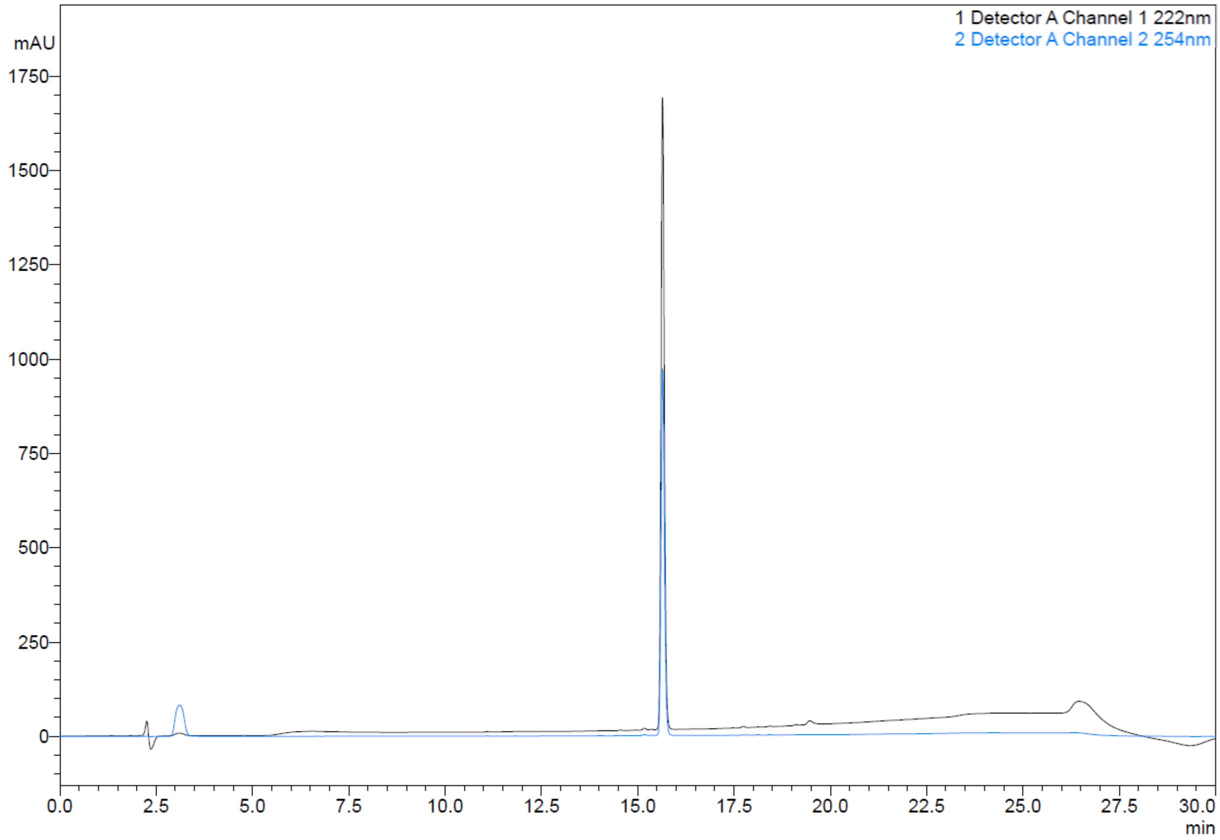

Figure S54. Analytical HPLC analysis of compound **4l**.

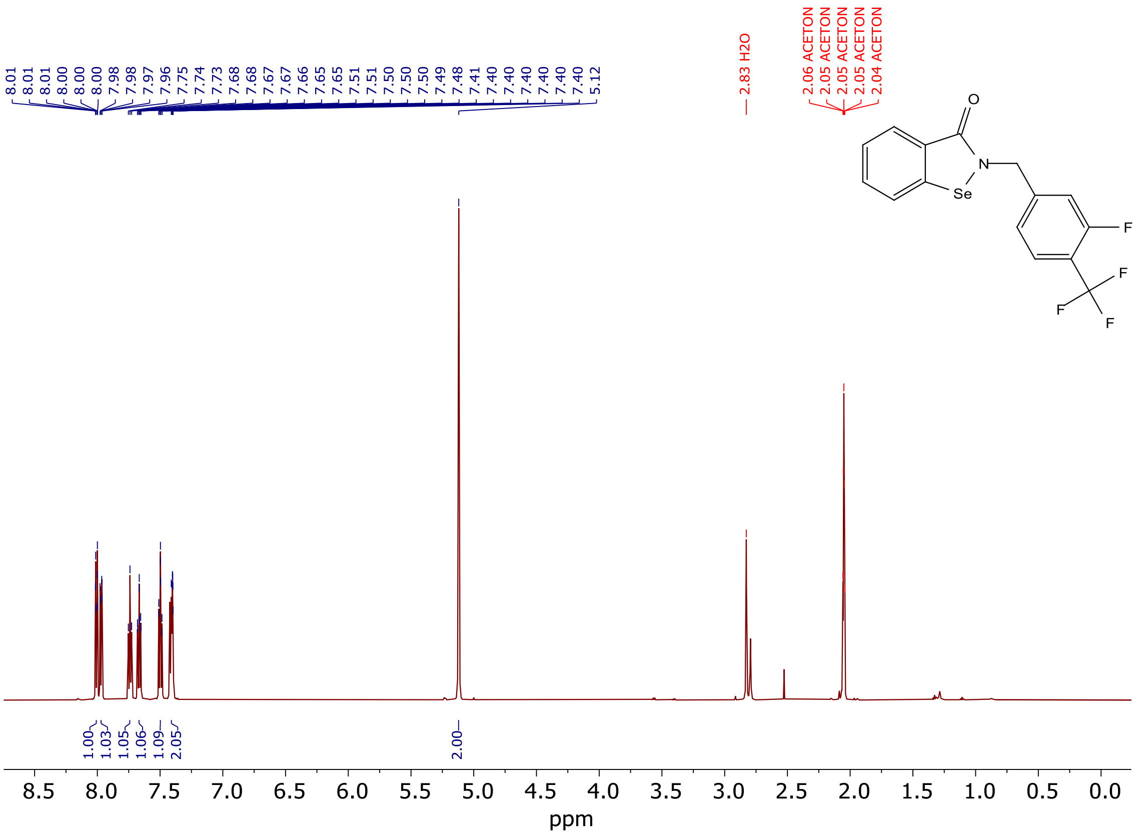

Figure S55. ^{1}H NMR spectrum of compound **4m**.

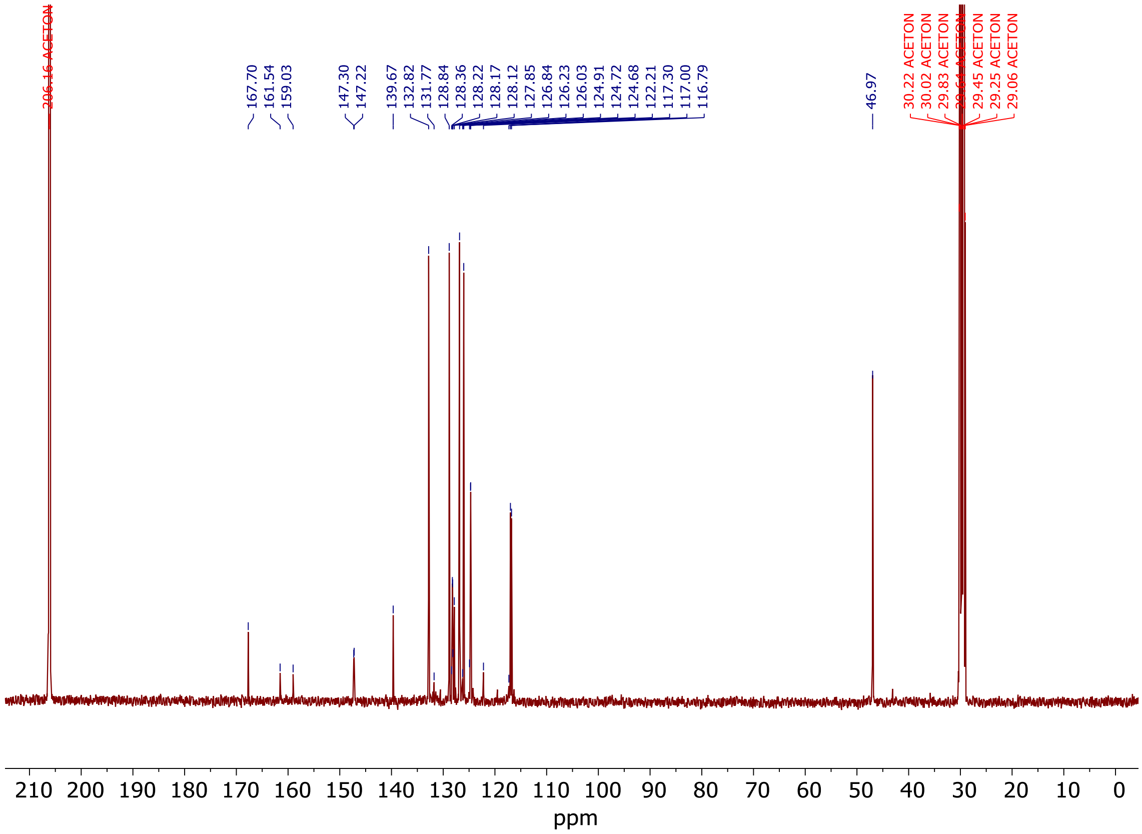

Figure S56. ^{13}C NMR spectrum of compound **4m**.

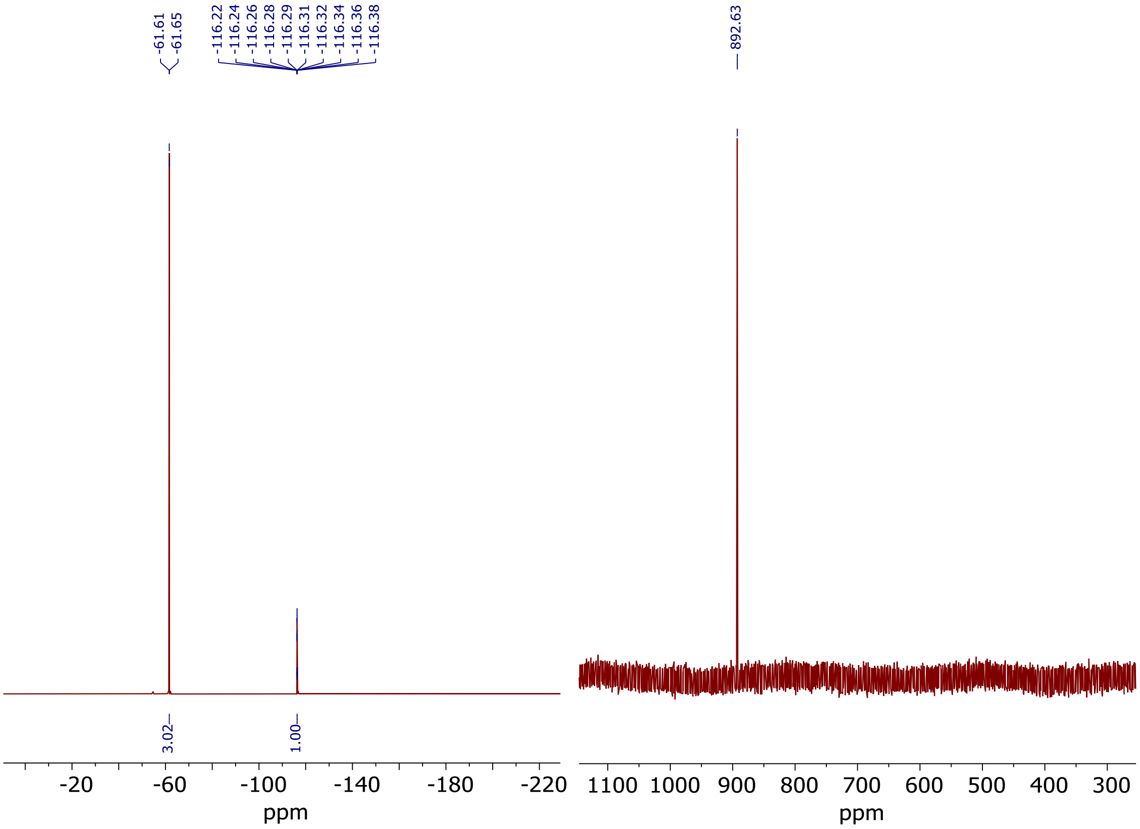

Figure S57. ^{19}F and ^{77}Se NMR spectra of compound **4m**.

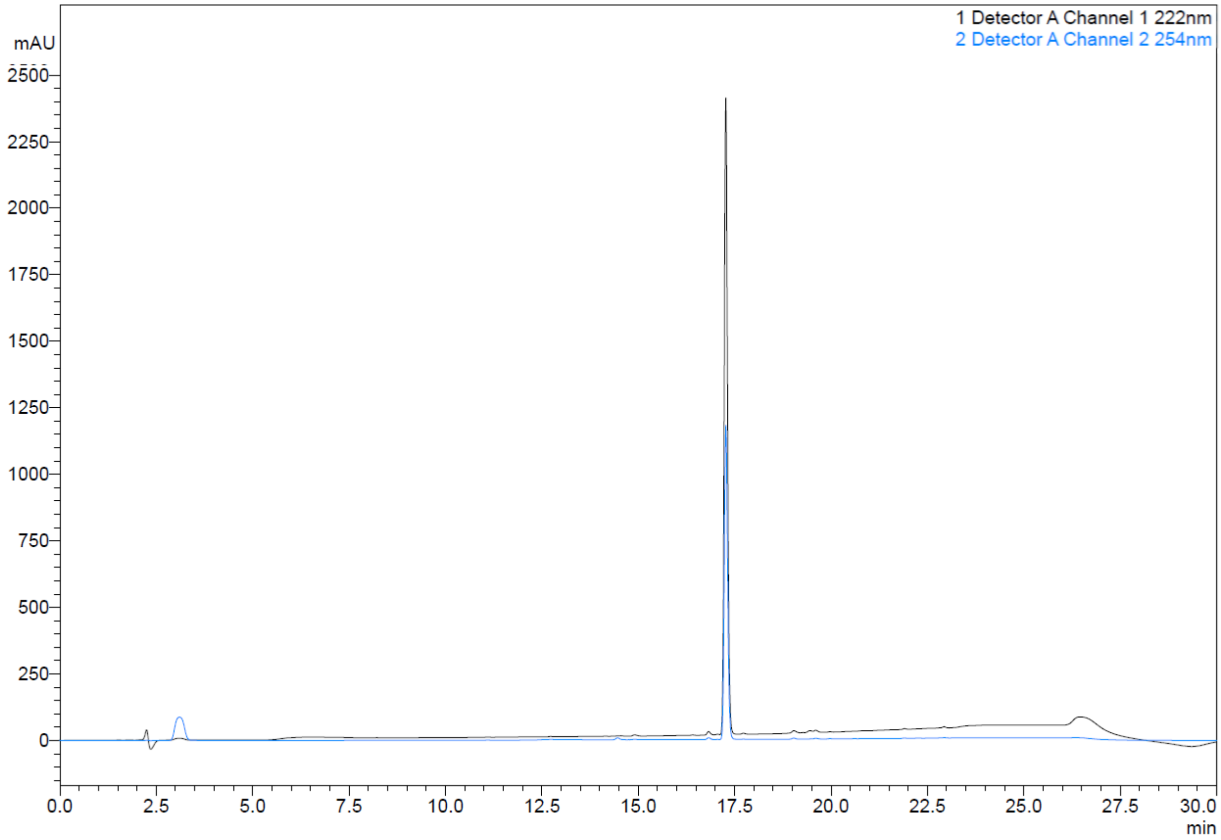

Figure S58 Analytical HPLC analysis of compound **4m**.

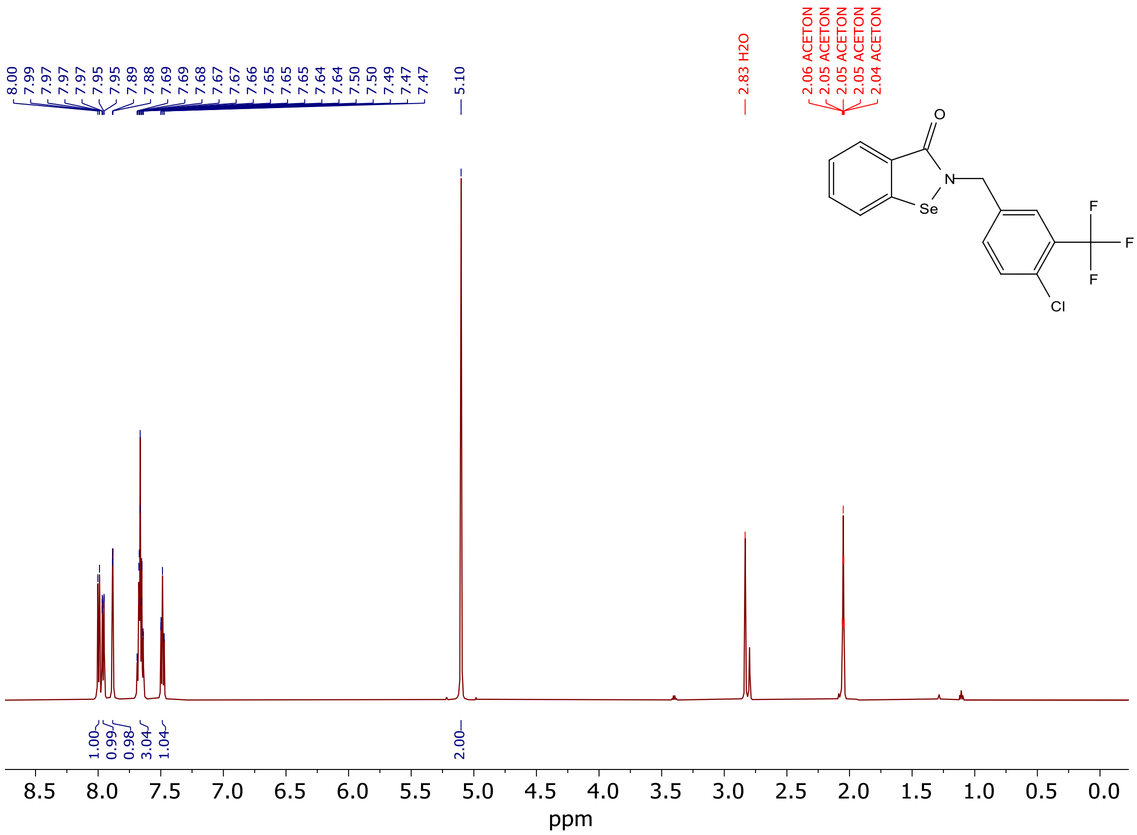

Figure S59. ^{1}H NMR spectrum of compound **4n**.

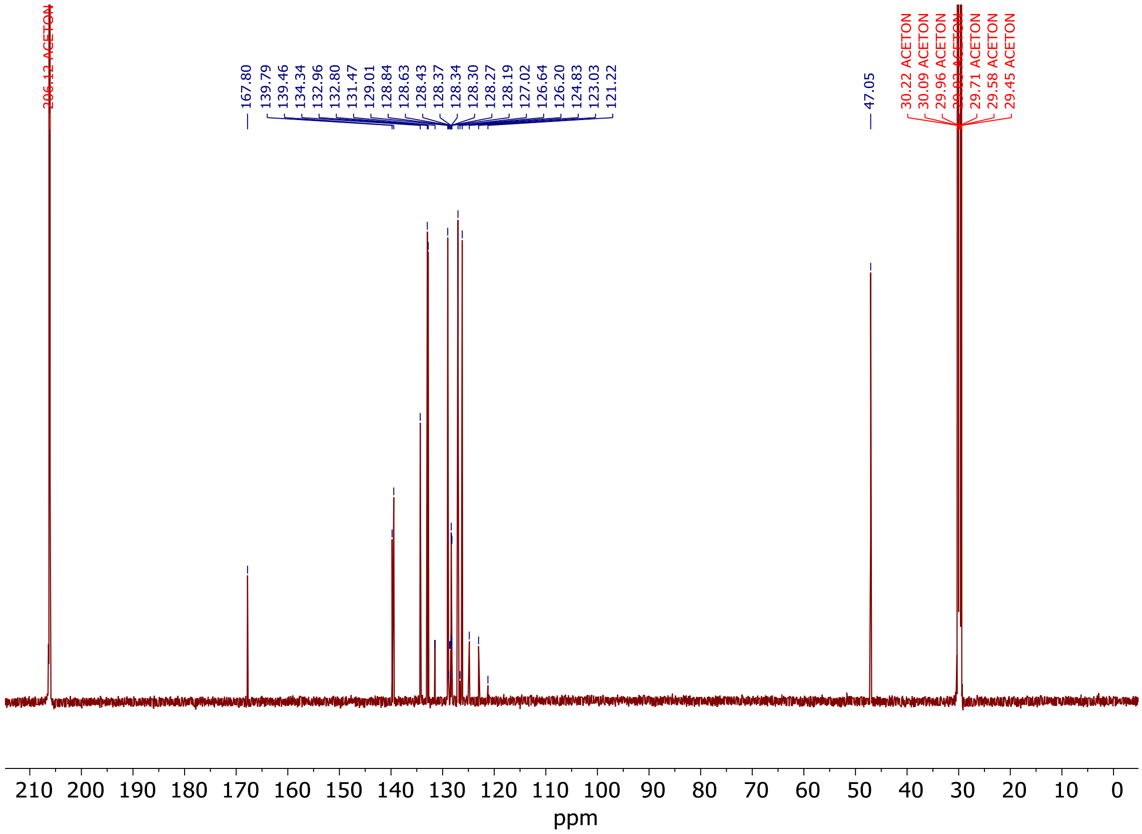

Figure S60. ^{13}C NMR spectrum of compound **4n**.

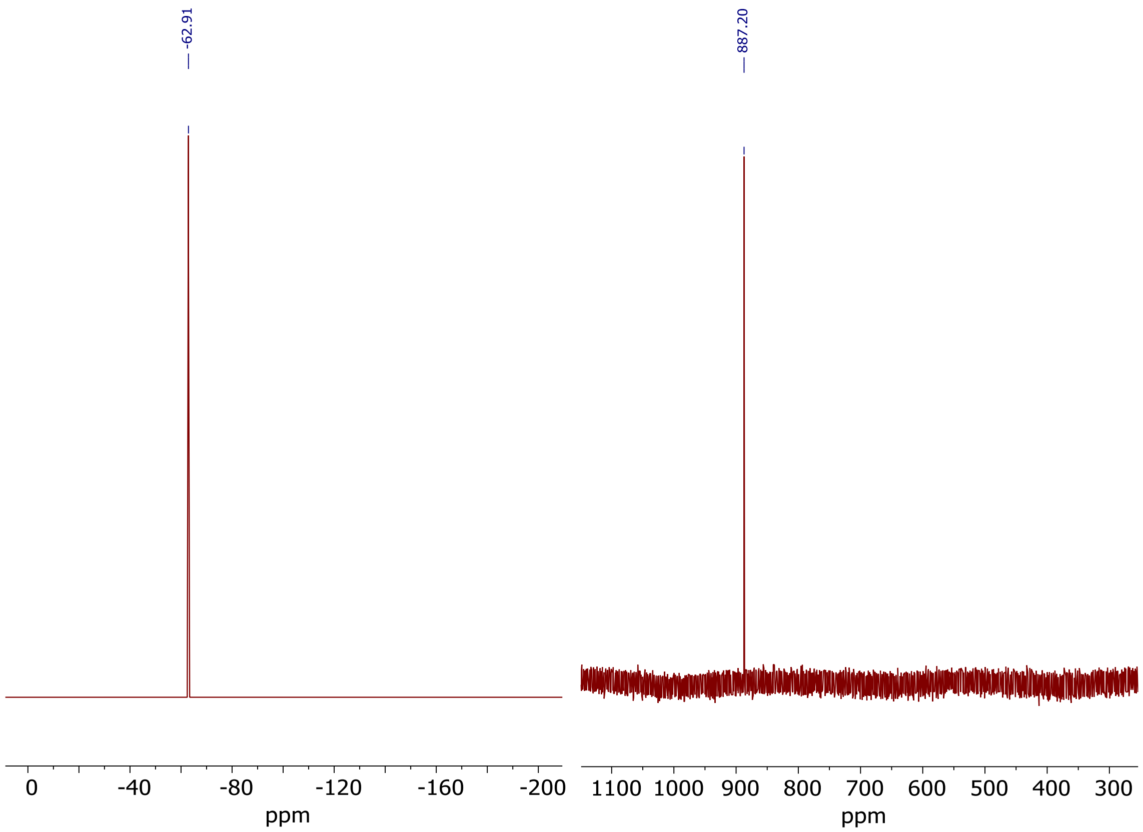

Figure S61. ^{19}F and ^{77}Se NMR spectra of compound **4n**.

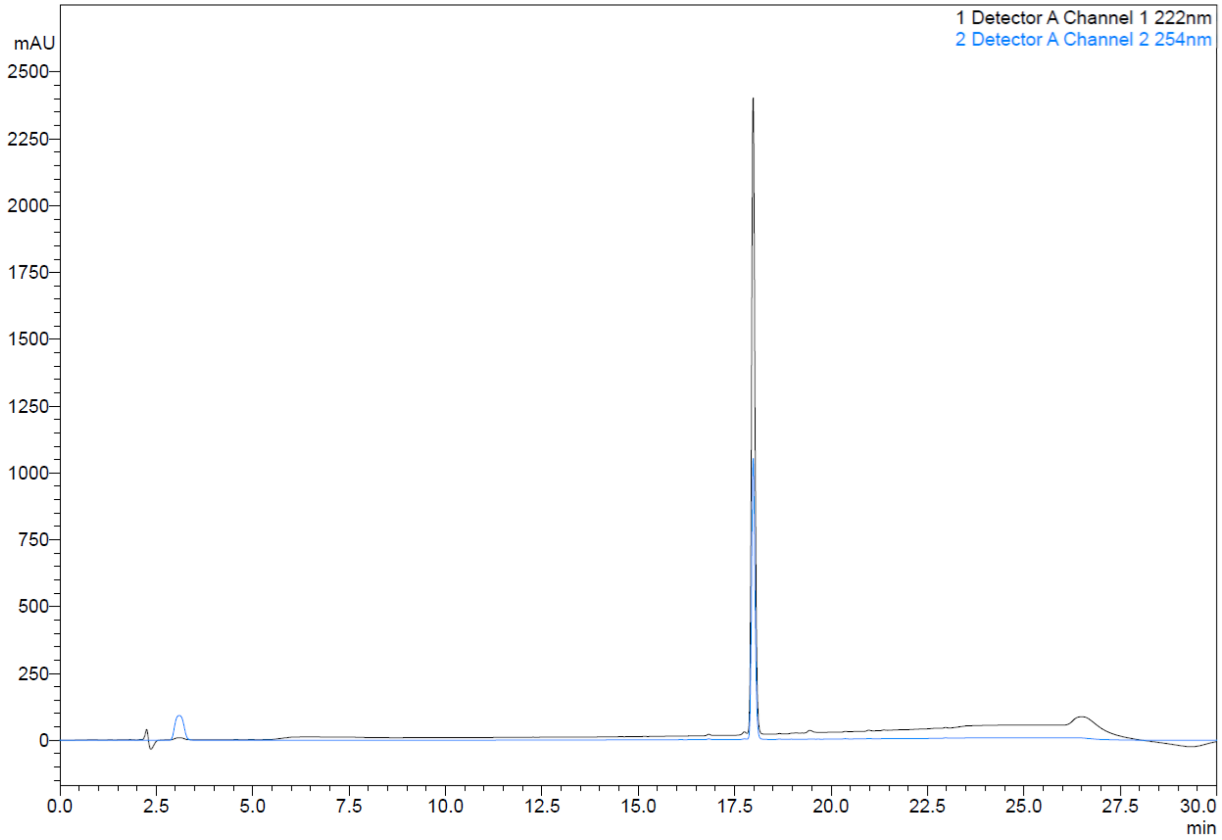

Figure S62. Analytical HPLC analysis of compound **4n**.

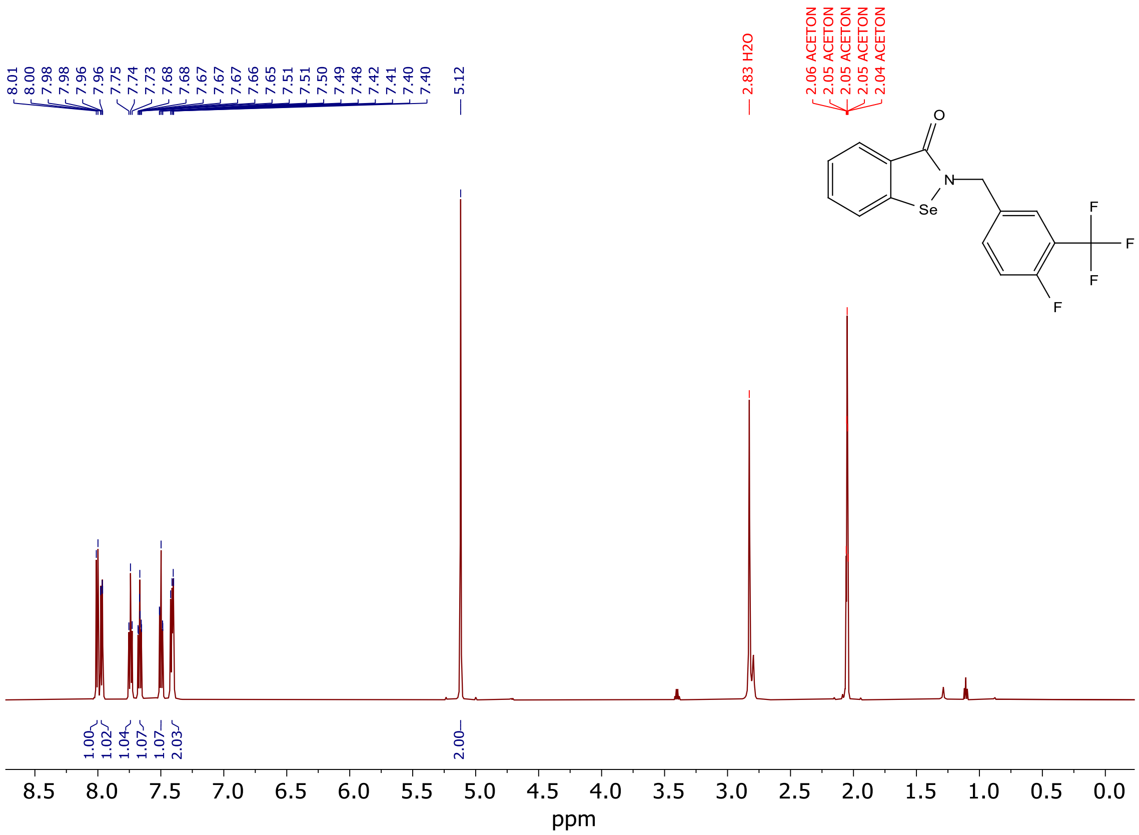

Figure S63. ^{1}H NMR spectrum of compound **4o**.

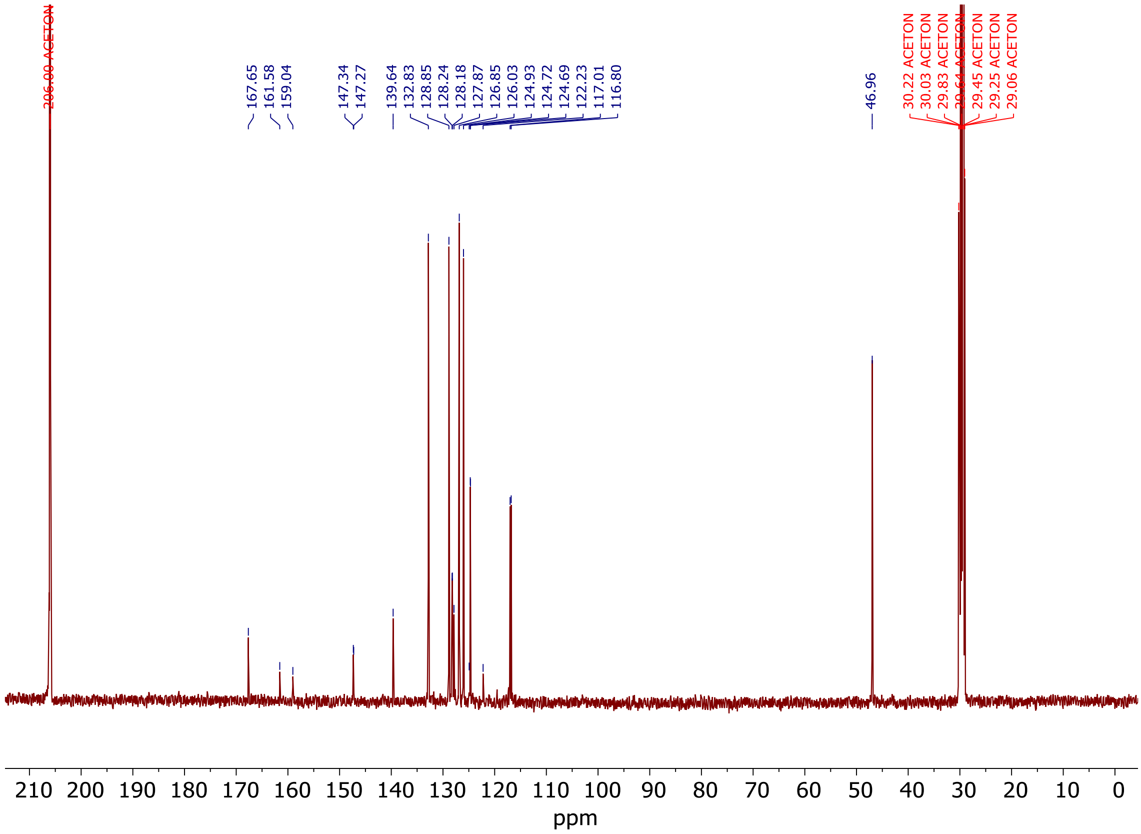

Figure S64. ^{13}C NMR spectrum of compound **4o**.

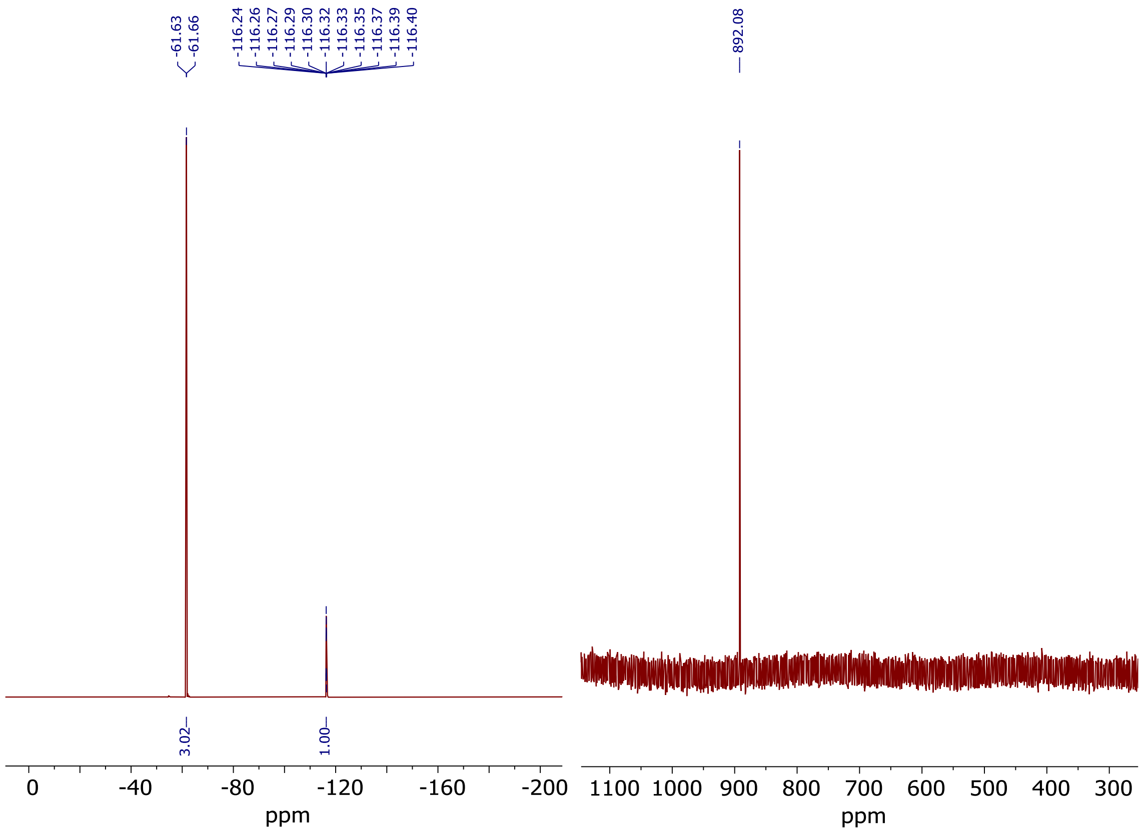

Figure S65. ^{19}F and ^{77}Se NMR spectra of compound **4o**.

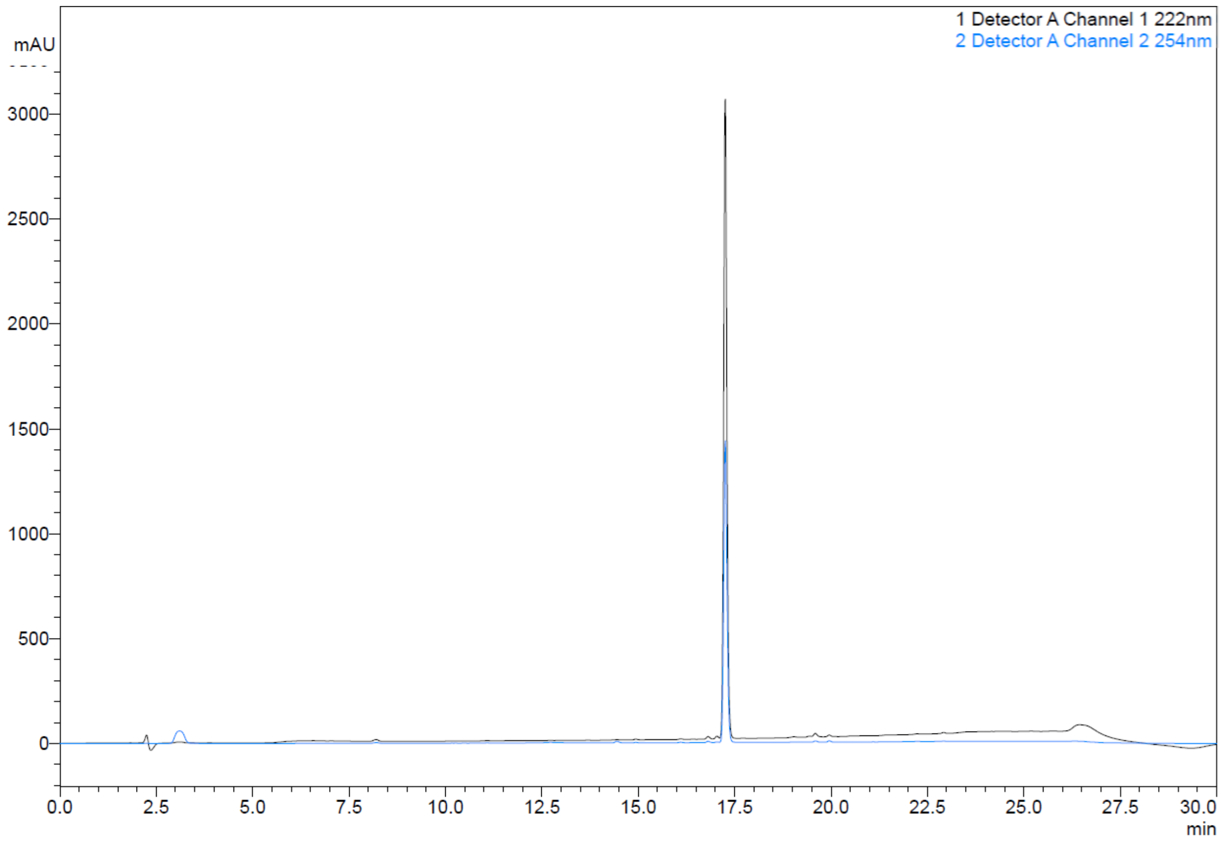

Figure S66. Analytical HPLC analysis of compound **4o**.

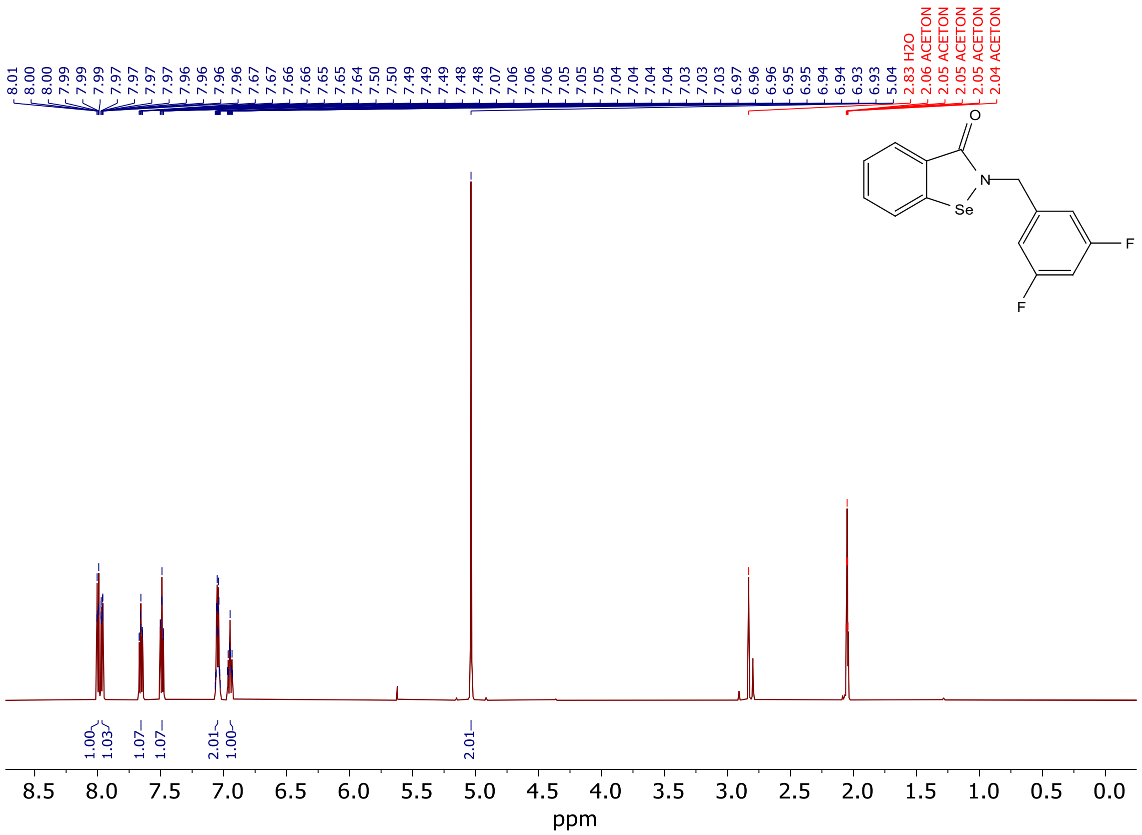

Figure S67. ^{1}H NMR spectrum of compound **4p**.

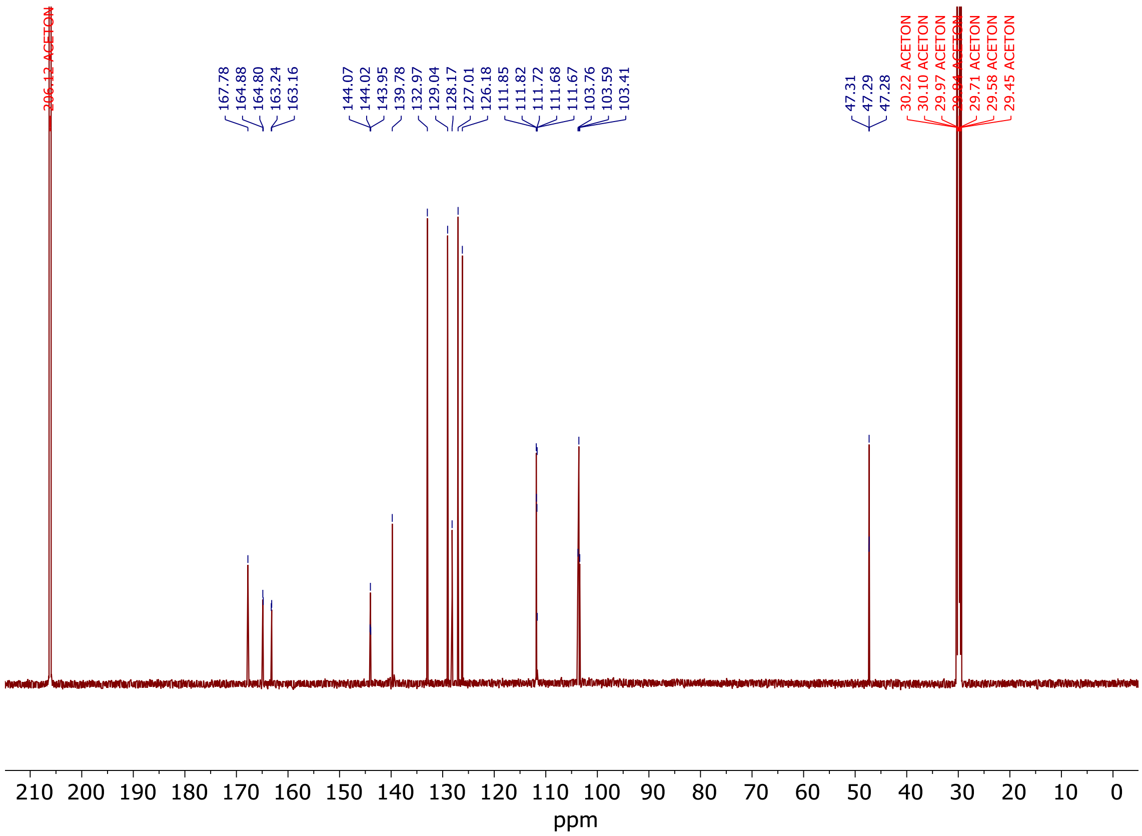

Figure S68. ^{13}C NMR spectrum of compound **4p**.

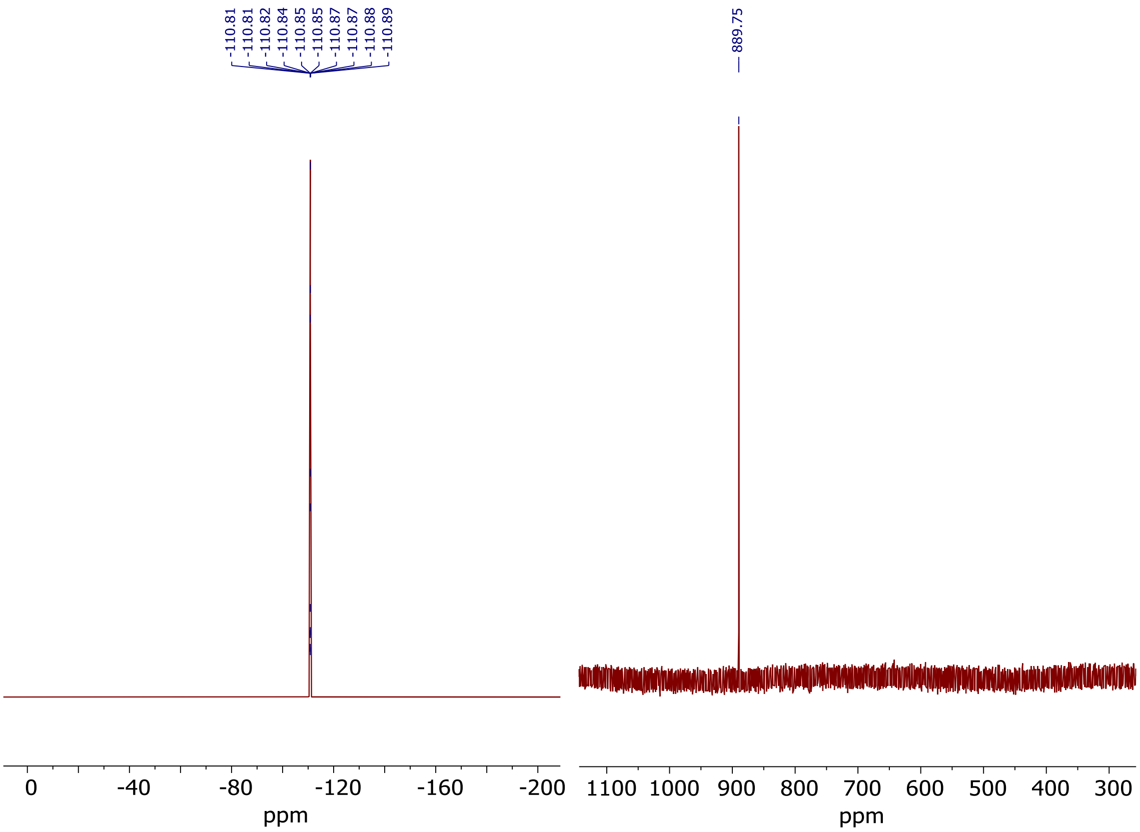

Figure S69. ^{19}F and ^{77}Se NMR spectra of compound **4p**.

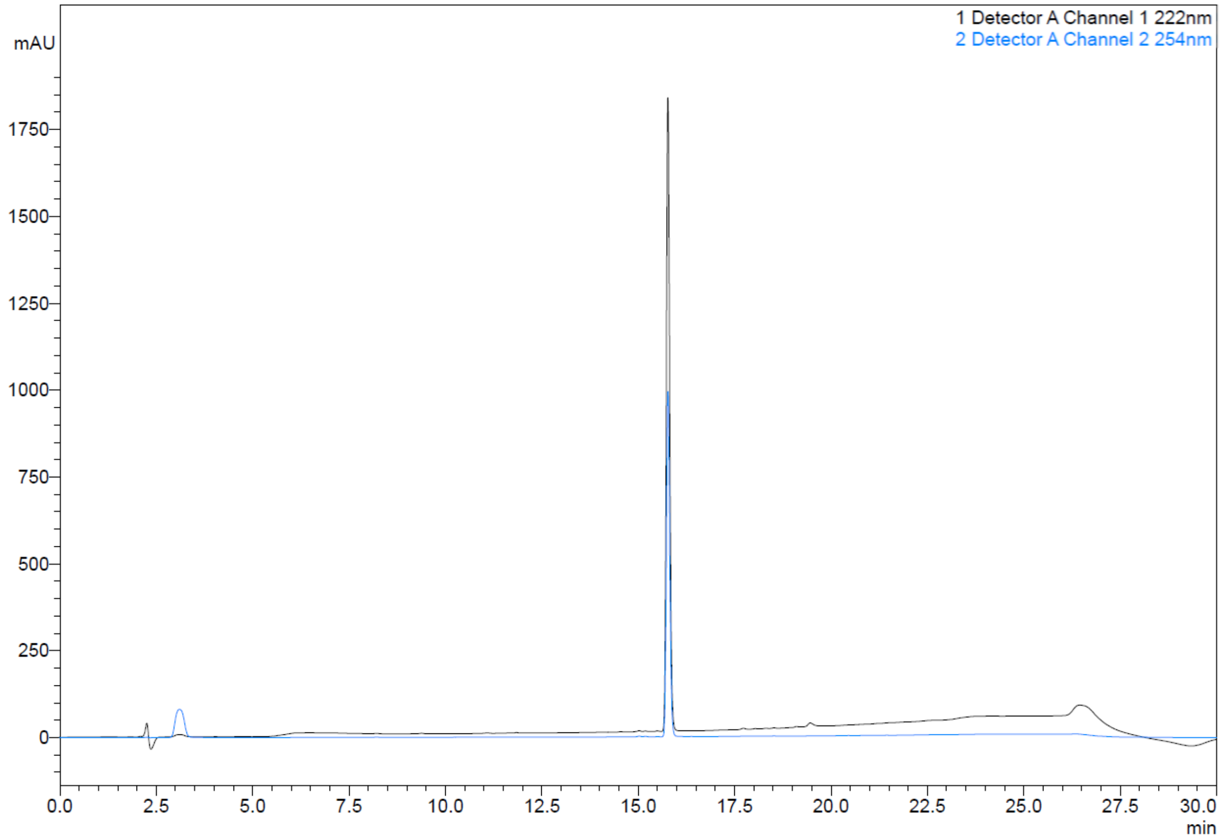

Figure S70. Analytical HPLC analysis of compound **4p**.

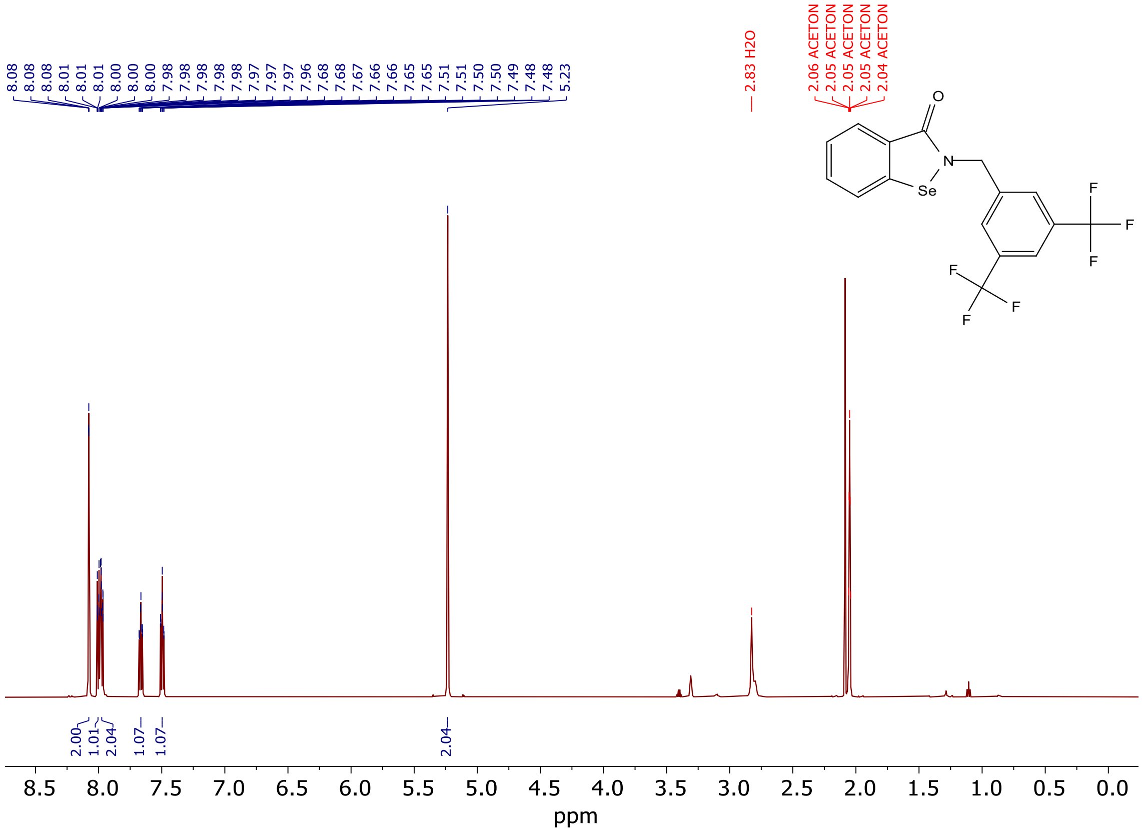

Figure S71. ^{1}H NMR spectrum of compound **4q**.

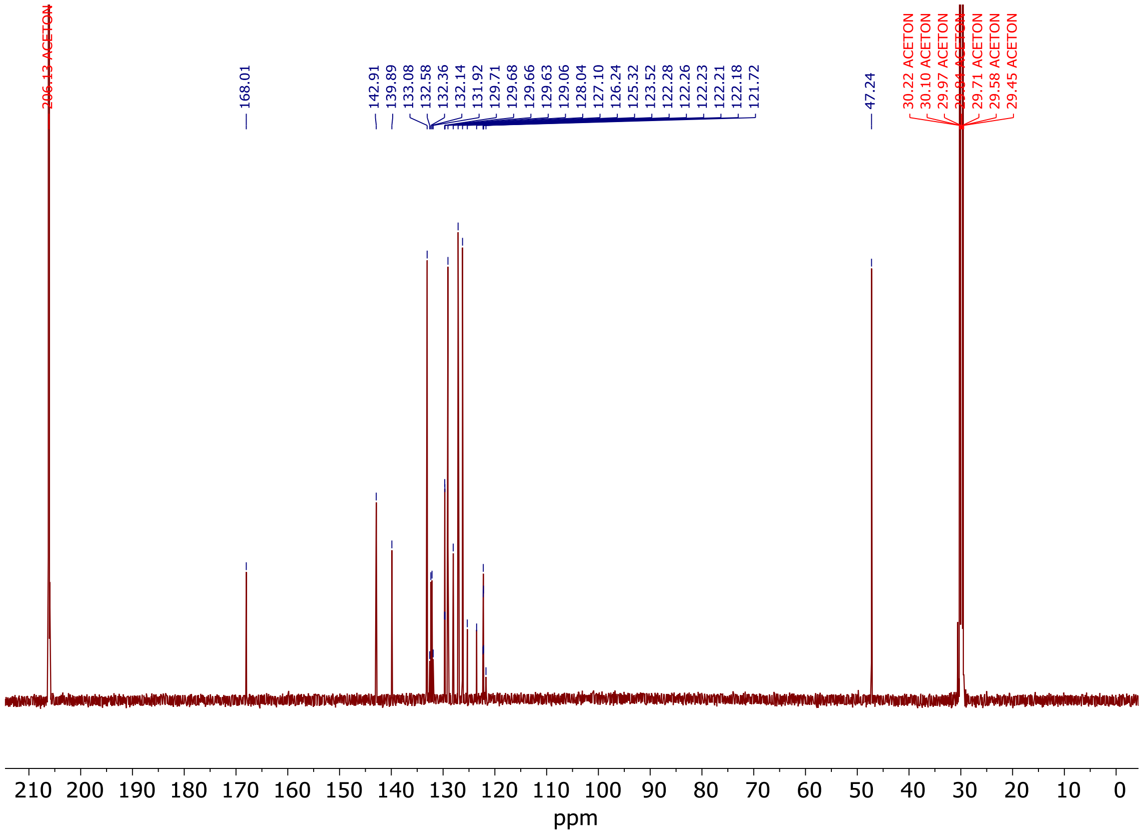

Figure S72. ^{13}C NMR spectrum of compound **4q**.

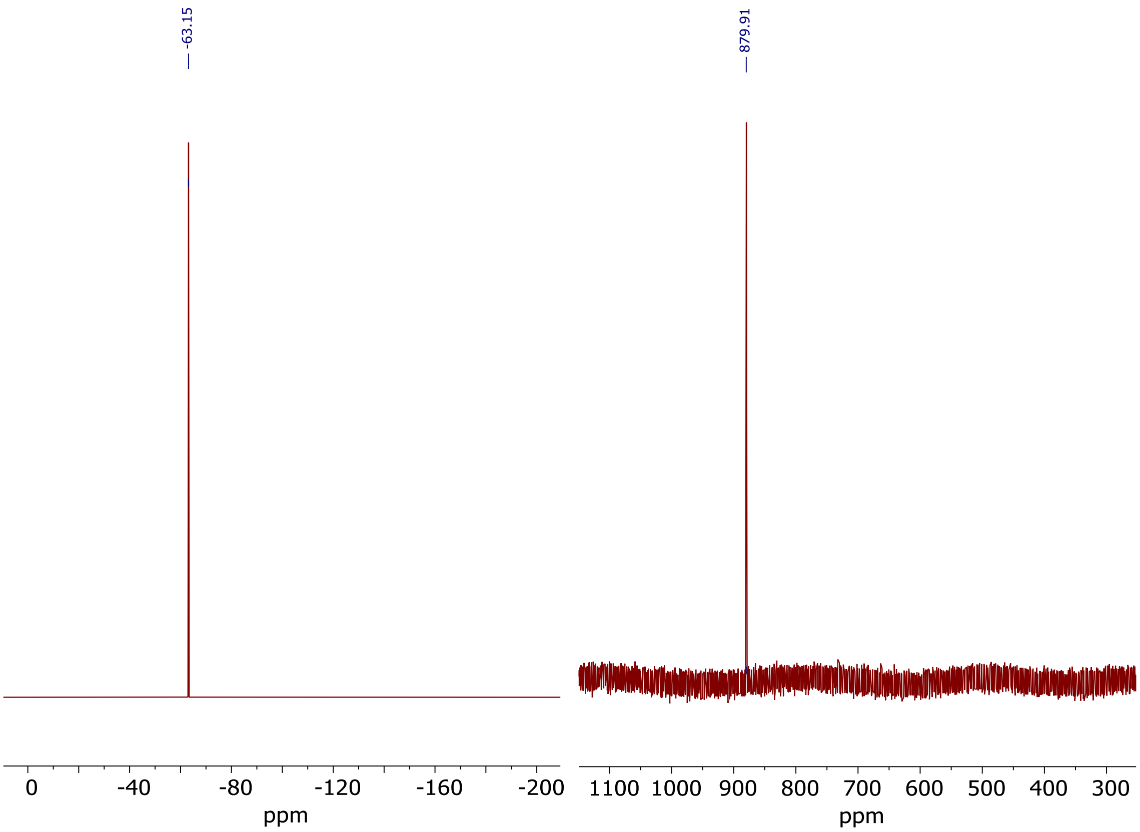

Figure S73. ^{19}F and ^{77}Se NMR spectra of compound **4q**.

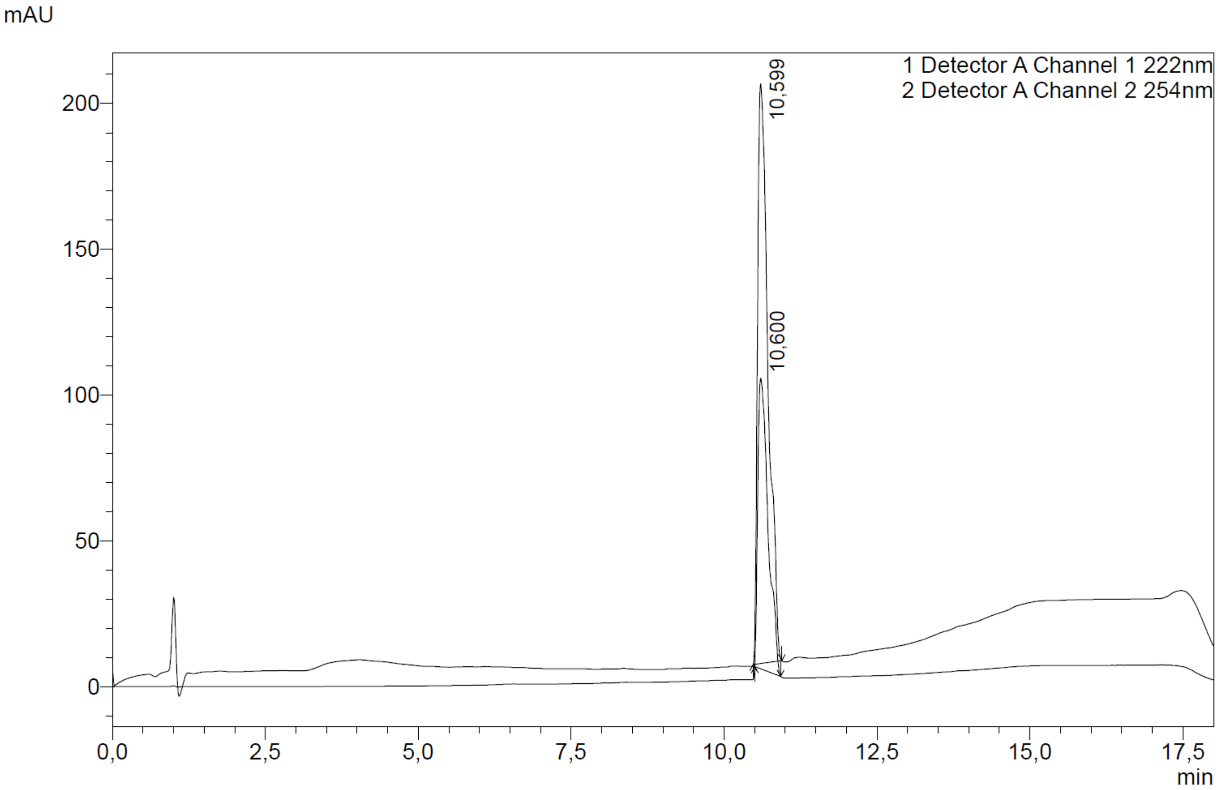

Figure S74. Analytical HPLC analysis of compound **4q** (CHROMSHELL C18-XB HPLC column, 4.6 × 75 mm, (0 min, 10% B → 1 min, 10% B → 12.5 min, 90% B → 15 min, 90% B → 17 min, 10% B, flow 1.0 mL/min).

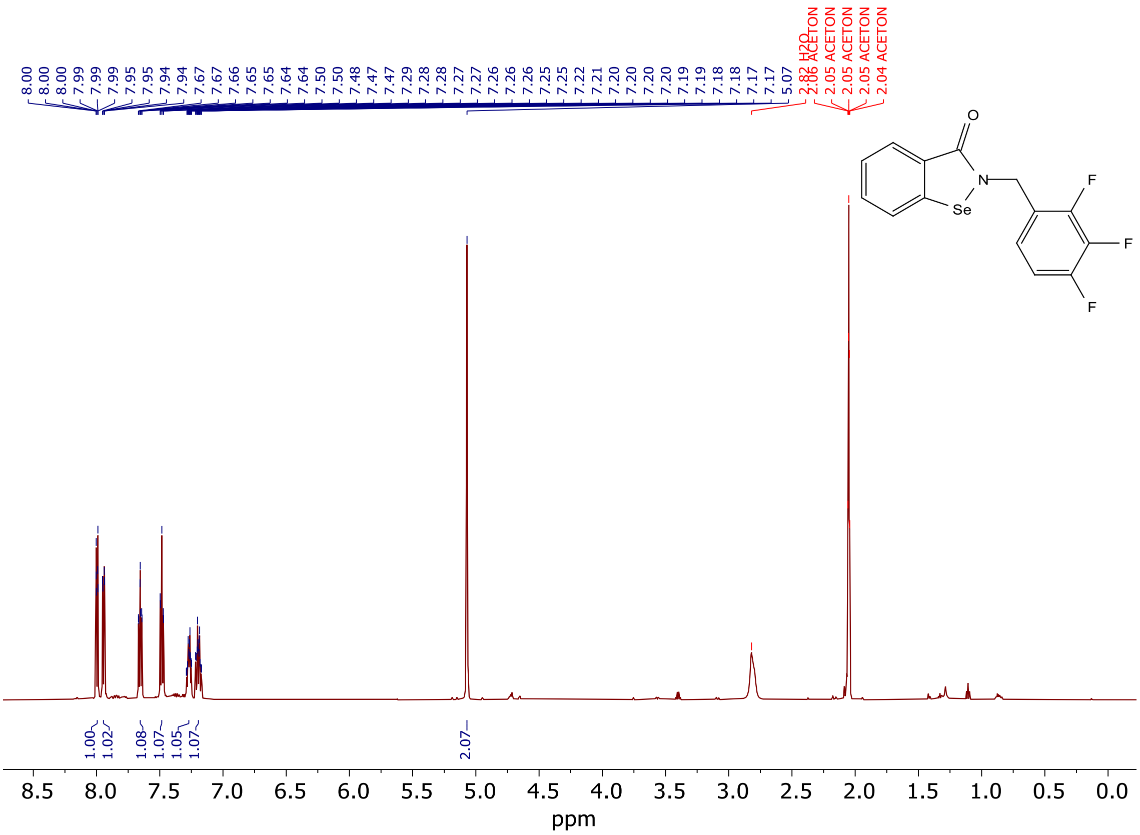

Figure S75. ^{1}H NMR spectrum of compound **4r**.

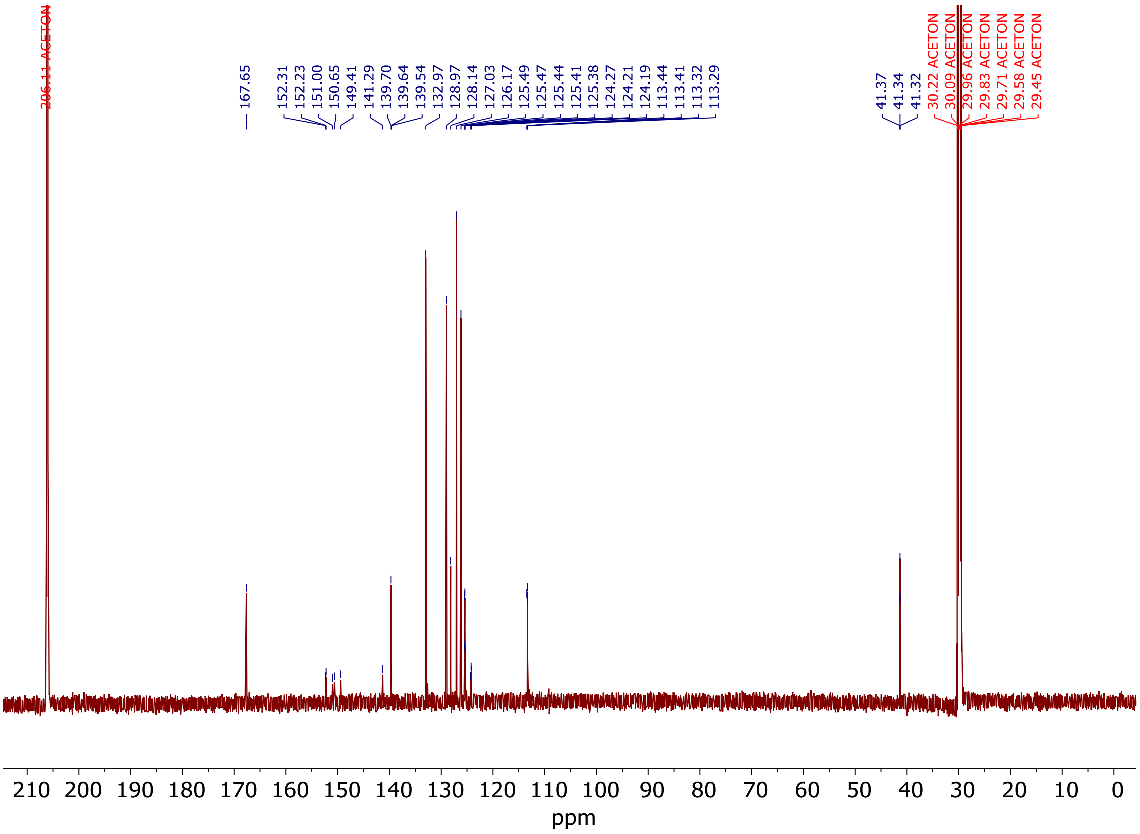

Figure S76. ^{13}C NMR spectrum of compound **4r**.

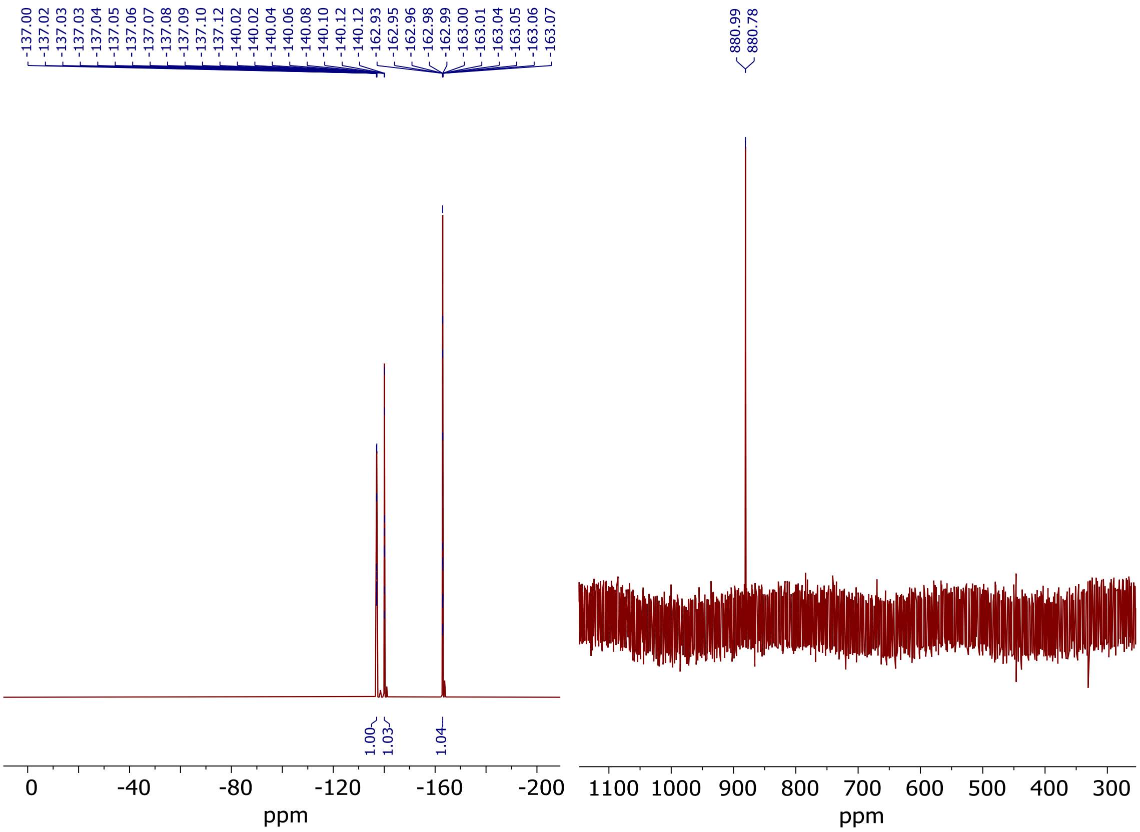

Figure S77. ^{19}F and ^{77}Se NMR spectra of compound **4r**.

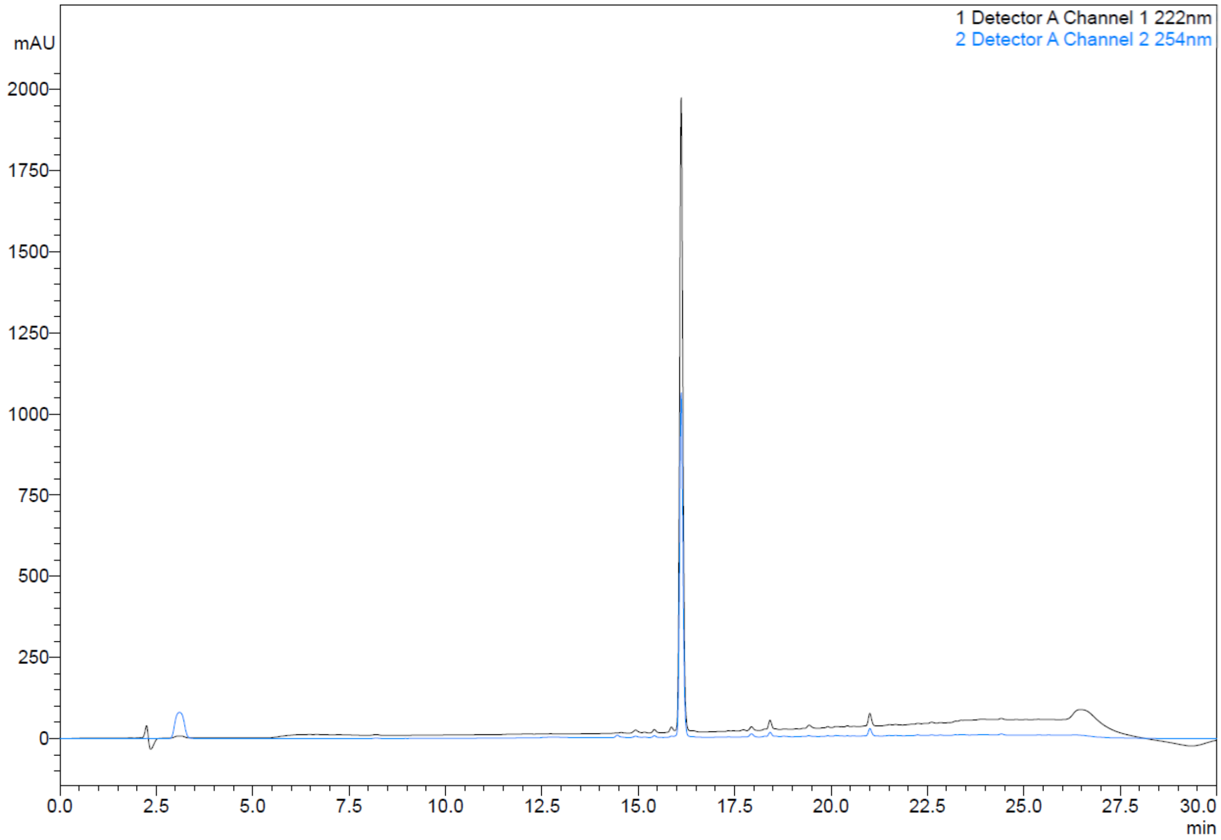

Figure S78. Analytical HPLC analysis of compound **4r**.
